# Low striatal T3 is implicated in inattention and memory impairment in an ADHD mouse model overexpressing thyroid hormone-responsive protein

**DOI:** 10.1038/s42003-021-02633-w

**Published:** 2021-09-20

**Authors:** Raly James Perez Custodio, Mikyung Kim, Leandro Val Sayson, Hyun Jun Lee, Darlene Mae Ortiz, Bung-Nyun Kim, Hee Jin Kim, Jae Hoon Cheong

**Affiliations:** 1 Uimyung Research Institute for Neuroscience, Department of Pharmacy, Sahmyook University, 815 Hwarang-ro, Nowon-gu, Seoul, 01795 Republic of Korea; 2 Department of Chemistry & Life Science, Sahmyook University, 815 Hwarang-ro, Nowon-gu, Seoul, 01795 Republic of Korea; 3 Department of Psychiatry and Behavioral Science, College of Medicine, Seoul National University, 101 Daehakro, Jongno-gu, Seoul, 03080 Republic of Korea; 4 School of Pharmacy, Jeonbuk National University, 567 Baekje-daero, Deokjin-gu, Jeonju-si, Jeollabuk-do 54896 Republic of Korea

**Keywords:** Disease model, Transgenic organisms, Developmental disorders

## Abstract

Attention-deficit/hyperactivity disorder (ADHD) is a neurodevelopmental disorder, potentially with a biological basis; however, its exact cause remains unknown. Thyroid hormone (TH) abnormalities are more prevalent in patients with ADHD than in the general population, indicating a shared pathogenetic mechanism for these conditions. Previously, we identified that overexpression of thyroid hormone-responsive protein (THRSP), a gene highly responsive to TH status, induced inattention in male mice. Herein, we sought to explore whether TH function in THRSP-overexpressing (THRSP OE) mice influences ADHD-like (inattention) behavior. We now confirm that THRSP overexpression in male mice reproduces behavioral features of ADHD, including sustained inattention and memory impairment, accompanied by excessive theta waves that were found normal in both the THRSP-knockout and hetero groups. Physiological characterization revealed low striatal T3 levels in the THRSP OE mice due to reduced striatal T3-specific monocarboxylate transporter 8 (MCT8), indicating brain-specific hypothyroidism in this transgenic mouse strain. TH replacement for seven days rescued inattention and memory impairment and the normalization of theta waves. This study further supports the involvement of the upregulated THRSP gene in ADHD pathology and indicates that THRSP OE mice can serve as an animal model for the predominantly inattentive subtype of ADHD.

## Abstract (web summary)

Custodio et al. characterise a mouse model of ADHD in which thyroid hormone-responsive protein is overexpressed. They demonstrate that reduced levels of striatal triiodothyronine (T3) play a role in inattention and memory impairment in this model and that the model could be used in future studies examining the predominantly inattentive subtype of ADHD.

## Introduction

The discovery of thyroid hormone-responsive protein (THRSP) 40 years ago^1^ has contributed substantially to our current knowledge of its role in lipid metabolism and biosynthesis^2,3^. THRSP, also called Spot 14 (S14), is a 17 kDa nuclear acidic protein expressed primarily in tissues synthesizing fatty acids, such as the breast tissues, liver, and white or brown fat^4,5^. THRSP is also expressed in the motor cortex, striatum, and hypothalamus^6^, demonstrating that THRSP is present in the brain and involved in CNS function. At the transcriptional level, THRSP regulates the expression of genes encoding metabolic enzymes required for *de novo* synthesis of long-chain fatty acids, such as fatty acid synthase (Fasn)^7^. The most striking aspect of THRSP is its strong response to thyroid hormone (TH)^1^. TH activity mediates its effects via its receptors (TRs),;TRα, and TRβ^8–10^. The ubiquitously expressed human THRSP has been reported as a cofactor for TRβ-dependent transcriptional activation of specific gene targets^11^, thus indicating that THRSP may modulate TRβ function downstream of the TH response elements (TRE). In this respect, mutation of TRβ leads to a condition referred to as generalized resistance to TH (GRTH) disorder^12,13^, which results in reduced responsiveness of tissues to TH activity.

Adequate amounts of TH (i.e., thyroxine (T4) and triiodothyronine (T3)) are essential for numerous physiological processes. The brain, being the center of the nervous system, is a critical target of TH which regulates neuronal cell proliferation, migration, and synaptogenesis^14^. Correspondingly, alterations in TH levels (i.e., hypothyroidism and GRTH) during fetal and postnatal periods cause developmental delays^15^. In adulthood, hypothyroidism induces neuronal network abnormalities, resulting in profound behavioral and neurological defects, including inattention and memory impairment^16–18^. Indeed, several psychiatric disorders are associated with TH abnormalities, including attention-deficit/hyperactivity disorder (ADHD)^13^. ADHD is a neurodevelopmental disorder, potentially with a biological basis; however, its exact cause remains unknown. A relationship between TH abnormalities and ADHD is derived from the observation that 46–70% of children with GRTH have ADHD^12^. Additionally, studies show a higher prevalence of thyroid abnormalities in children with ADHD (5.4%) than in the general population (<1%)^13^, thereby suggesting a shared pathogenetic mechanism.

Recent studies have implicated the THRSP gene in ADHD. Microarray analysis identified several differentially expressed genes in cortical samples from inattentive SHR/Ncrl and WKY/Ncrl ADHD animal models; among these genes, upregulated THRSP presented the highest overall association^19^. Correspondingly, functional overexpression of THRSP in mice induced inattention and dysregulated dopaminergic activity in the striatum, which was improved by treatment with methylphenidate^20^, further supporting the role of THRSP in ADHD.

Since THRSP is highly responsive to TH status changes, we sought to explore whether TH function in THRSP-overexpressing (THRSP OE) mice influences its ADHD-like (inattention) behavior. To this end, we utilized behavioral, encephalographic, pharmacological, and molecular tests. For comparison, we evaluated the responses of THRSP Hetero (heterozygous), knockout (KO; homozygous), and wild-type (WT) mice. Here we found that reduction in striatal T3 levels influences the inattention, memory impairment, and theta waves reduction in THRSP OE mice, indicating the role of TH in ADHD pathogenesis, particularly in the predominantly inattentive subtype of ADHD (ADHD-PI).

## Results

### The THRSP transgenic mice

**Fig. 1:**
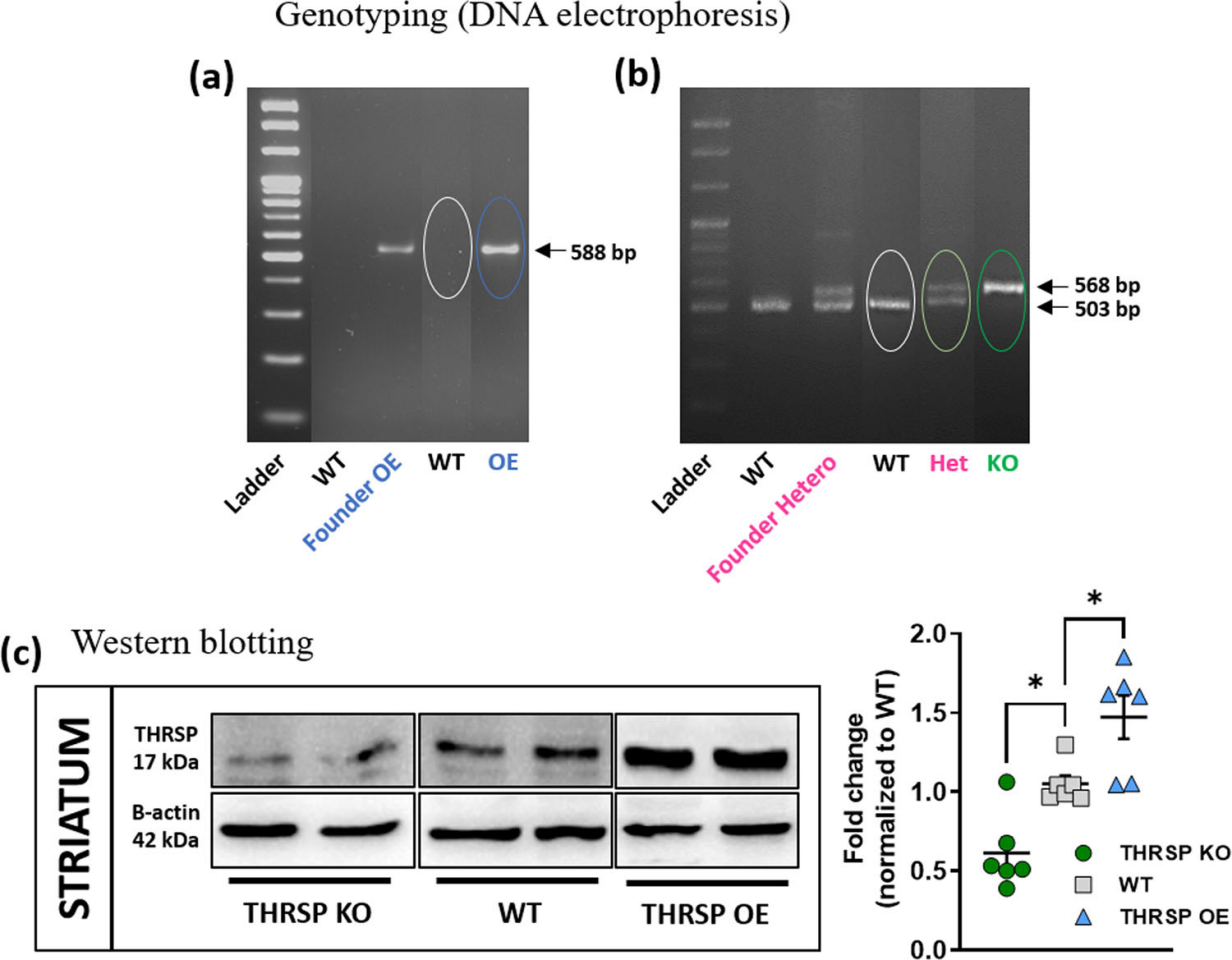
The confirmation of mouse genotype and striatal THRSP levels. Before the start of each experiment, genotyping was conducted in 3-week-old mice using tail samples. **a** The THRSP OE mice show one band  at 588 bp; in contrast, **b** the Hetero mice show two bands (568 and 503 bp), and KO mice show one band (568 bp). **c** Western blot using striatal tissue samples confirm the overexpression of THRSP protein levels in THRSP OE mice and the reduced expression in THRSP KO mice (*n* = 6 mice/group; **c** one-way ANOVA, *F*_(3,20)_ = 15.0, *P* < 0.001). Values are presented as the mean ± S.E.M. *P < 0.05, by one-way ANOVA with Bonferroni’s multiple comparisons relative to WT.

Before each experiment, age-appropriate THRSP OE, Hetero, KO mice were identified by DNA gel electrophoresis using specific polymerase chain reaction (PCR) conditions and primers. The THRSP OE mice display a clear band at 588 bp (Fig. 1a), whereas the THRSP Hetero shows double bands at 568 and 503 bp, and THRSP KO mice show a single band at 568 bp (Fig. 1b). Subsequently, the expression of THRSP was evaluated in mice using western blot analysis (Fig. 1c, Supplementary Fig. 1a). We confirm the overexpression of THRSP in THRSP OE mice and the lack thereof in THRSP KO mice (Fig. 1d, e). Also, we found that the expression of THRSP in THRSP Hetero mice was not different from WT (Supplementary Fig. 1a).

### THRSP OE mice display sustained inattention and other ADHD-like phenotypes

**Fig. 2:**
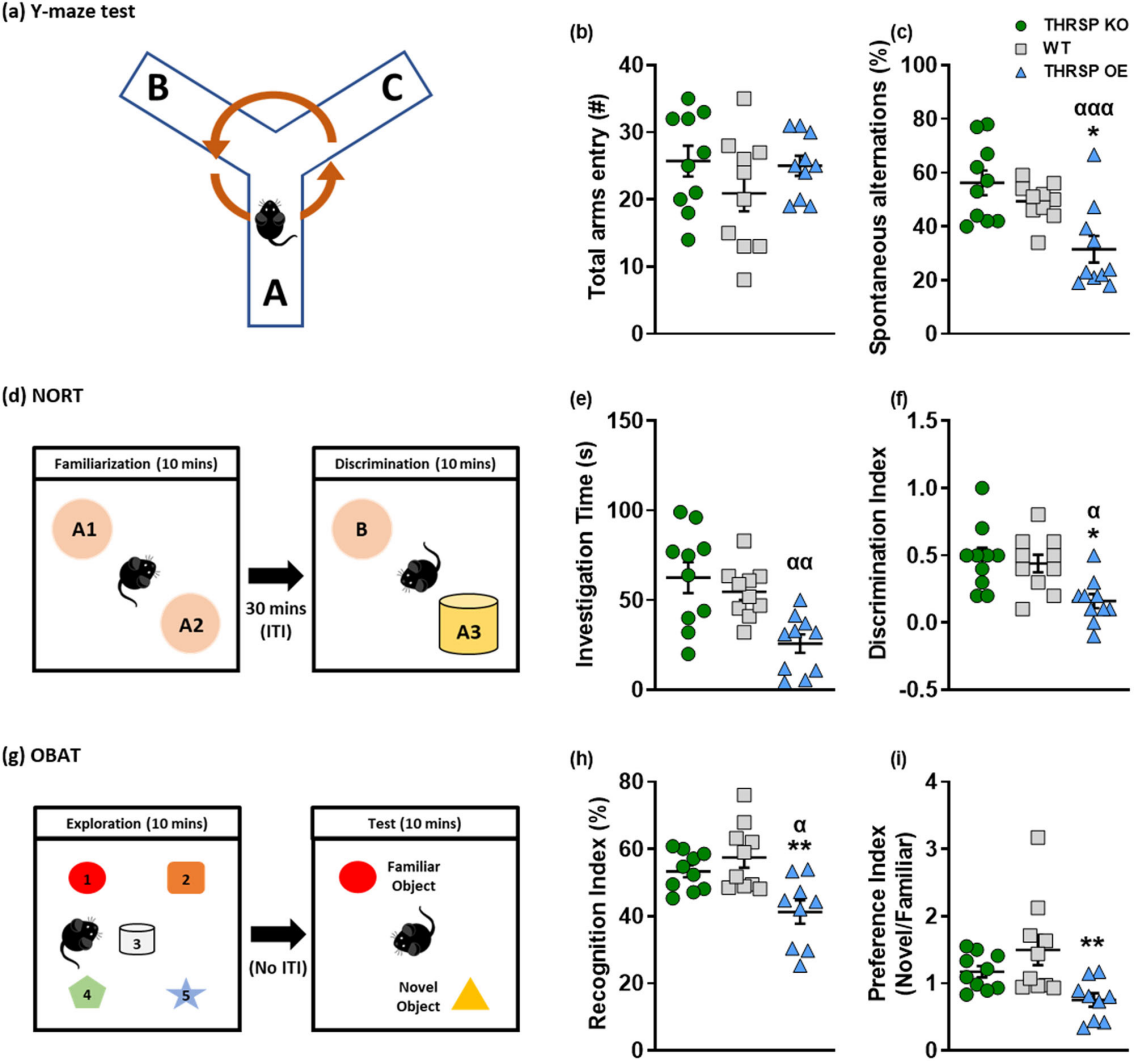
Sustained inattention in THRSP OE mice. Four weeks-old mice were exposed in the **a** Y-maze apparatus for 10 min and the **b** total arm entry and **c** spontaneous alternations were recorded (*n* = 10 mice/group; **b** one-way ANOVA, *F*_(3,36)_ = 1.04, *P* = 0.388; **c** one-way ANOVA, *F*_(3,36)_ = 7.13, *P* < 0.001). Subsequently, mice were subjected to the **d** NORT, where the **e** investigation time and **f** discrimination index were scored (*n* = 10 mice/group; **e** one-way ANOVA, *F*_(3,36)_ = 4.71, *P* = 0.007; **f** one-way ANOVA, *F*_(3,36)_ = 4.44, *P* = 0.009). Then, additional mice were exposed to **g** OBA test and the **h** recognition index and **i** object preference were recorded (*n* = 9–10 mice/group; one-way ANOVA, **h** *F*_(3,34)_ = 6.18, *P* = 0.002; **i** *F*_(3,34)_ = 4.17, *P* = 0.013). The THRSP OE mice showed lower spontaneous alternations, and the discrimination and recognition indices in Y-maze, novel-object recognition, and object-based attention tests, respectively. Moreover, the behaviors observed in the THRSP KO mice in these tests did not differ from that the WT mice. Values are presented as the mean ± S.E.M. *P < 0.05 and **P < 0.01 by one-way ANOVA with Bonferroni’s multiple comparison relative to WT. ^α^*P* < 0.05, ^αα^*P* < 0.01, and ^ααα^*P* < 0.001 by one-way ANOVA with Bonferroni’s multiple comparison relative to THRSP KO. ITI: Inter-trial interval.

The inattention observed previously in THRSP OE mice^20^ was sustained in the present study; the Y-maze test indicates low spontaneous alternation (Fig. 2c), and the novel-object recognition test (NORT) show reduced investigation time and discrimination index (Fig. 2e, f). Inarguably, initially, both the Y-maze and NORT are tools to measure memory^21,22^; However, more recently, studies have utilized these tests to measure attention, particularly those used in modeling ADHD-like behaviors in rodents^19,20,23–25^. Several preclinical ADHD studies have utilized the Y-maze test in modeling inattention, particularly the “spontaneous alternations” behavior. In the NORT, during “novelty,” or when something new to the environment is present, attention and exploration are involved which allow subjects to examine the objects present either closely or distally, depending on the risks. If something familiar is present, it also requires attention and reevaluation of the subjects^26^. Our results found that THRSP OE mice had lower investigation time observed during the familiarization phase of the NORT. A decreased time spent investigating the objects during the familiarization phase is an initial display of inattentive behavior or inattention^23^. Thus, the reduced discrimination index of THRSP OE mice might also result from their inattention to the objects in the familiarization phase.

However, to further confirm the inattention in THRSP OE mice, we utilized the object-based attention test (OBAT), a modification of NORT, used as a measure of object-based attention in rodents^27–29^. Here, we found that the THRSP OE mice have low recognition index (Fig. 2h). The failure to recognize between familiar and novel object observed in THRSP OE mice corroborate our initial findings of their inattention (i.e., Y-maze, NORT). Moreover, we found that these transgenic mice have reduced preference index (Fig. 2i). The principle of this parameter is when new objects are present; attention usually shifts to that object. Also, preference for novel objects indicates that the familiar object’s presentation exists in mice’s memory^30^; however, this was not evident in THRSP OE mice as they did not prefer the novel object, which can then be an indication of inattention. Given the similarity of findings in NORT and OBAT, particularly with the discrimination and recognition or preference indices, respectively, we propose using NORT as a measure of inattention in mice. Overall, these indicate that the inattentive “behavioral phenotype” manifests in newer generations of this transgenic mouse model of ADHD.

Furthermore, the THRSP KO and Hetero (Supplementary Fig. 1b–g) mice did not show inattention compared to the WT mice, corroborating the results from our previous study, which showed that the upregulation of THRSP is involved in inattention^19^. Additionally, we assessed other behaviors in the THRSP KO and Hetero mice. Notably, their motor activity in the open-field test (OFT) (Supplementary Fig. 2a, b) and impulse behavior in the cliff-avoidance tests (Supplementary Fig. 2c, d) did not differ from that of WT, indicating that the THRSP KO and Hetero mice do not demonstrate hyperactivity and impulsivity. Also, the anxiety observed in the elevated-plus maze (EPM) (Supplementary Fig. 2e, f) and motor balance impairment as assessed by performance in the rotarod tests (Supplementary Fig. 2g, h) were absent in these strains, which further confirms that THRSP is an inattention-specific genetic marker.

**Fig. 3:**
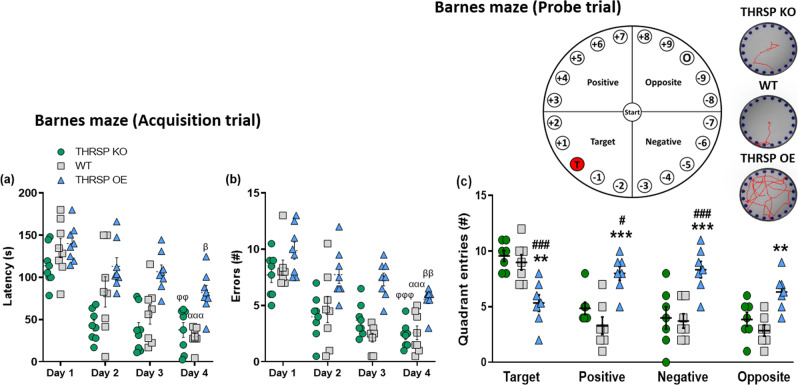
Memory impairment in THRSP OE mice. Mice were subjected to the Barnes maze test for 4 days of acquisition trials to assess the **a** latency and **b** errors in identifying the target hole with the “goal” or escape box (*n* = 8 mice/group; **a** repeated measures two-way ANOVA, *F*_(2,21)_ = 24.6, *P* < 0.001; **b** repeated measures two-way ANOVA, *F*_(2,21)_ = 22.3, *P* < 0.001). Subsequently, short-term memory was assessed on the 5th day in a probe trial, in which individual mice were assessed on the basis of **c** frequency of visits towards each platform quadrant (i.e., target, positive, opposite, and negative) (*n* = 7 mice/group; **c** two-way ANOVA, *F*_(2,72)_ = 11.5, *P* < 0.001). Latency and errors improved throughout the acquisition trials; however, THRSP OE mice exhibited longer latency and higher number of errors when locating the target hole compared with other strains. Additionally, they spent more time in other quadrants during the probe trial on the 5th day, suggesting a memory impairment in THRSP OE mice. Values are presented as the mean ± S.E.M. α (WT) *P* < 0.001, φ (THRSP KO) *P* < 0.01, and β (THRSP OE) *P* < 0.05 according to repeated measures two-way ANOVA with Bonferroni’s multiple comparison tests relative to the performance of individual strains during day 1 vs. 4 of acquisition trials. **P < 0.01, ***P < 0.001 (relative to WT) and ^#^*P* < 0.05, ^###^*P* < 0.001 (relative to THRSP KO) according to two-way ANOVA with Bonferroni’s multiple comparison tests.

Considering that memory impairment is a comorbid disorder in ADHD^31,32^, we assessed spatial memory in mice using the Barnes maze test. As expected, during the first day of trial acquisition, there was no difference between the strains in latency time and number of errors when locating the target hole (escape box location) (Fig. 3a, b; Supplementary Fig. 1h, i). The latency and errors declined; however, the THRSP OE mice continued to exhibit longer latency and higher number of errors when locating the target hole than WT and THRSP KO mice. During the removal of the escape box (probe trial), all strains repeatedly visit and spend more time in the target quadrant (Fig. 3c; Supplementary Fig. 1j), except for the THRSP OE mice. The THRSP OE mice used a random strategy in locating the platform, thereby spending an equal or higher number of visits to other quadrants (i.e., positive, negative, and opposite), indicating an impairment in memory.

In support of the behavioral effects observed in our transgenic mice, we evaluated brain waves using electroencephalography (EEG). The Food and Drug Administration (FDA) of USA has recently authorized the use of Neuropsychiatric EEG-Based ADHD Assessment Aid (NEBA) based on quantitative electroencephalogram (qEEG), which includes brain wave testing as a complementary tool for evaluating ADHD^33^. While the EEG equipment can measure all brain waves, only one parameter was measured: theta waves. Mouse theta waves, the frequency of which is 4.5–8 cycles per second (Hz), reflect the brain in a state of sleep or daydreaming while being awake. Children and adults with ADHD show excessively lower-frequency theta waves, indicating problems with focus, attention, and learning^34^.

**Fig. 4:**
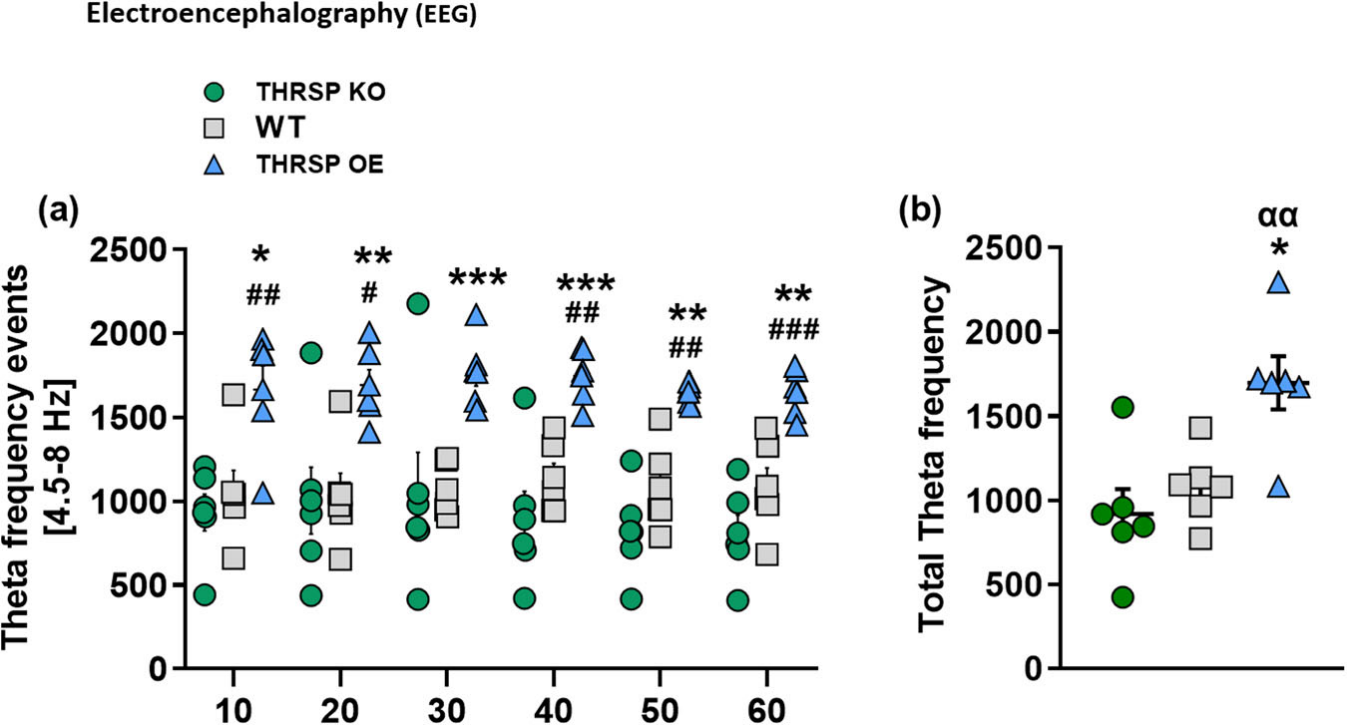
THRSP-overexpression enhances theta waves in mice. Following all behavioral tests, mice underwent EEG evaluation, and the **a** theta waves frequency events and **b** total theta frequencies were analyzed (*n* = 6 mice/group; **a** repeated measures two-way ANOVA, *F*_(2,15)_ = 16.5, *P* < 0.001; **b** one-way ANOVA, *F*_(2,15)_ = 9.30, *P* = 0.002). THRSP-overexpression enhanced the theta waves in mice. Values are presented as the mean ± S.E.M. *P < 0.05, **P < 0.01, ***P < 0.01 (relative to WT) and ^#^*P* < 0.05, ^##^*P* < 0.01, ^###^*P* < 0.001, by repeated measures two-way ANOVA with Bonferroni’s multiple comparison tests (Theta frequency events). *P < 0.05 (relative to WT) and ^αα^*P* < 0.01 (relative to THRSP KO), by one-way ANOVA with the Bonferroni’s multiple comparison tests (Total Theta frequency).

Interestingly, we observed a higher theta frequency event in the THRSP OE mice (Fig. 4a, b). Moreover, the THRSP KO and Hetero (Supplementary Fig. 1k, l) mice presented no changes compared to WT. This result suggests that the changes in theta waves presented by the THRSP OE mice may contribute to the inattention and memory impairment observed in their behavior.

### Overexpression of THRSP induces a reduction in striatal T3 levels in mice

Because the THRSP gene is innately responsive to TH, we evaluated its function in mice and determined its implications for the inattention and memory impairment observed in THRSP OE mice. We aimed to identify the TH levels in the brain, particularly in the striatum, considering the highly expressed THRSP mRNA and protein levels in this brain region of the THRSP OE mice, as shown in our previous study^20^. In addition, the striatum is sensitive to TH^35^, equally crucial in attention and memory^36,37^, and highly implicated in ADHD pathology^38^.

**Fig. 5:**
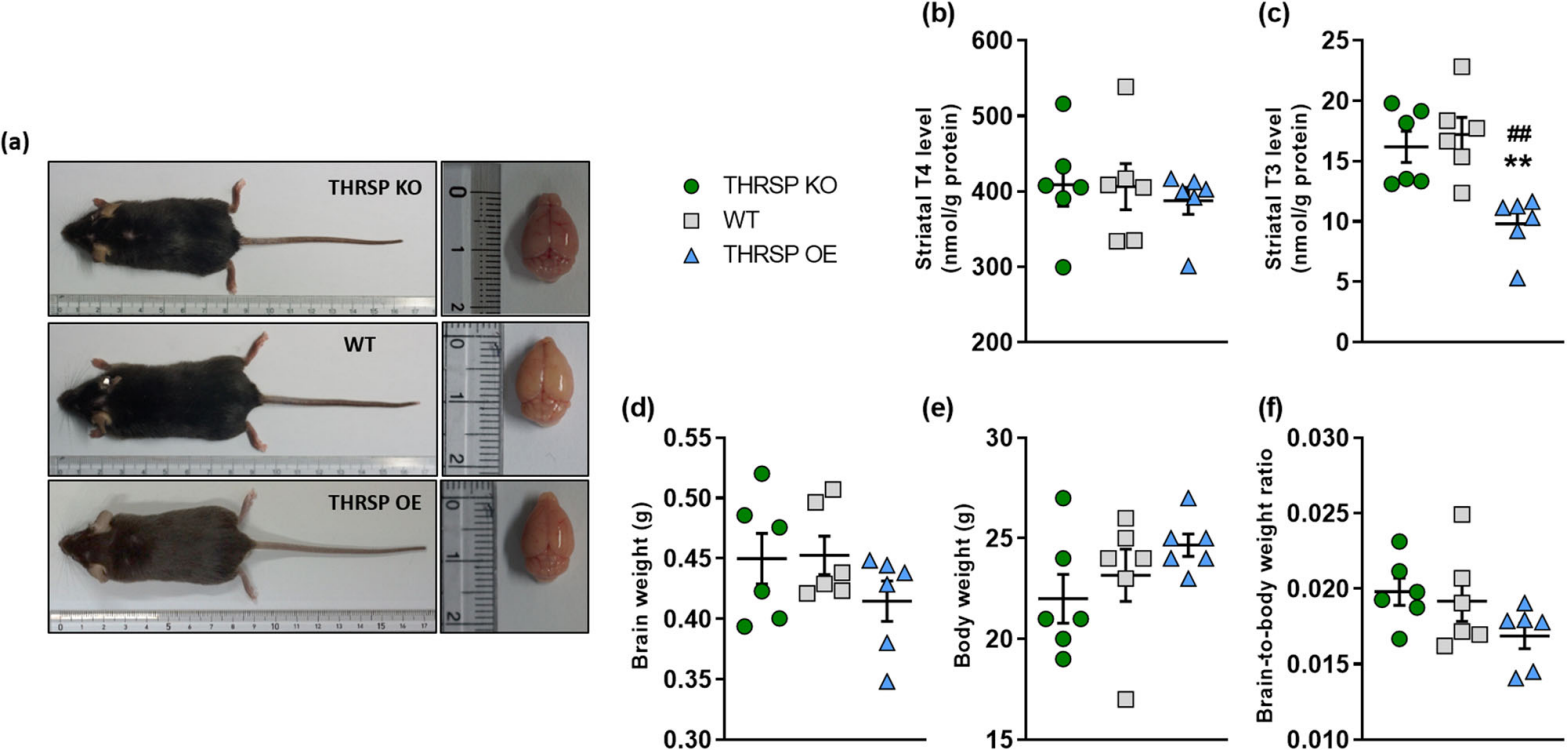
THRSP OE mice have reduced striatal T3 levels with moderately low brain-to-body weight ratio. **a** A snapshot of mice and their brains. **b** T4 and **c** T3 were evaluated (*n* = 6 mice/group; **b** one-way ANOVA, *F*_(2,15)_ = 0.198, *P* = 0.822; **c** one-way ANOVA, *F*_(2,15)_ = 10.4, *P* = 0.001). Also, the **d** brain and **e** body weights, and the corresponding **f** brain-to-body weight ratio in mice were assessed (*n* = 6 mice/group; **d** one-way ANOVA, *F*_(2,15)_ = 1.38, *P* = 0.282; **e** one-way ANOVA, *F*_(2,15)_ = 1.54, *P* = 0.246; **f** one-way ANOVA, *F*_(2,15)_ = 2.17, *P* = 0.148). THRSP overexpression reduced the striatal T3 levels in mice accompanied by moderately low brain-to-body weight ratio. Values are presented as the mean ± S.E.M. **P < 0.01 (relative to WT) and ^##^*P* < 0.01 (relative to THRSP KO), by one-way ANOVA with Bonferroni’s multiple comparison tests.

This study found that the THRSP OE mice exhibited normal T4 concentrations (Fig. 5a) but lacked striatal T3 levels (Fig. 5b). Furthermore, we found that the plasma levels of thyroid-stimulating hormone (TSH), T4, and T3 were normal (Supplementary Fig. 3a–c), indicating a functioning thyroid, at least in the blood or circulation but not in the brain. Also, we found an increased deiodinase 2 (*dio2*) mRNA levels (Supplementary Fig. 4b). Previous findings show that enhanced *dio2* activity^39,40^ increases the proportion of T3 formed locally in *dio2*-expressing tissues (such as the brain) under hypothyroidism conditions to mitigate the decrease of tissue T3 content^41,42^. These findings indicate that TH levels in the blood do not necessarily reflect the concentrations in critical organs such as the brain^43^.

In contrast, we found that the THRSP KO and Hetero mice exhibit normal TH levels (Fig. 5b, c; Supplementary Fig. 1n, o), corroborating the results of a previous study that demonstrated euthyroidism (a state of normal thyroid function) in mice following THRSP (Spot14) deletion^2,3^. We have established the potential effects of THRSP gene overexpression on striatal T3, leading to inattention and memory impairment in mice. In addition, we observed that the brain-to-body weight ratio (Fig. 5f; Supplementary Fig. 1r) (derived from the actual brain weight [Fig. 5d; Supplementary Fig. 1p] relative to the body weight [Fig. 5e; Supplementary Fig. 1q] was slightly lower in the THRSP OE mice. Although this measurement is a rough estimation of intelligence in humans and animals^44^, this may support the inattention and memory impairment observed in the THRSP OE mice and the absence thereof in THRSP KO and Hetero mice. Furthermore, we assessed other major organs (Supplementary Fig. 5b–g), including the heart, lungs, liver, spleen, kidneys, and testes, and found no difference in their weights relative to other strains.

### TH replacement improves ADHD-like behaviors and rescues striatal T3 levels in THRSP OE mice

**Fig. 6:**
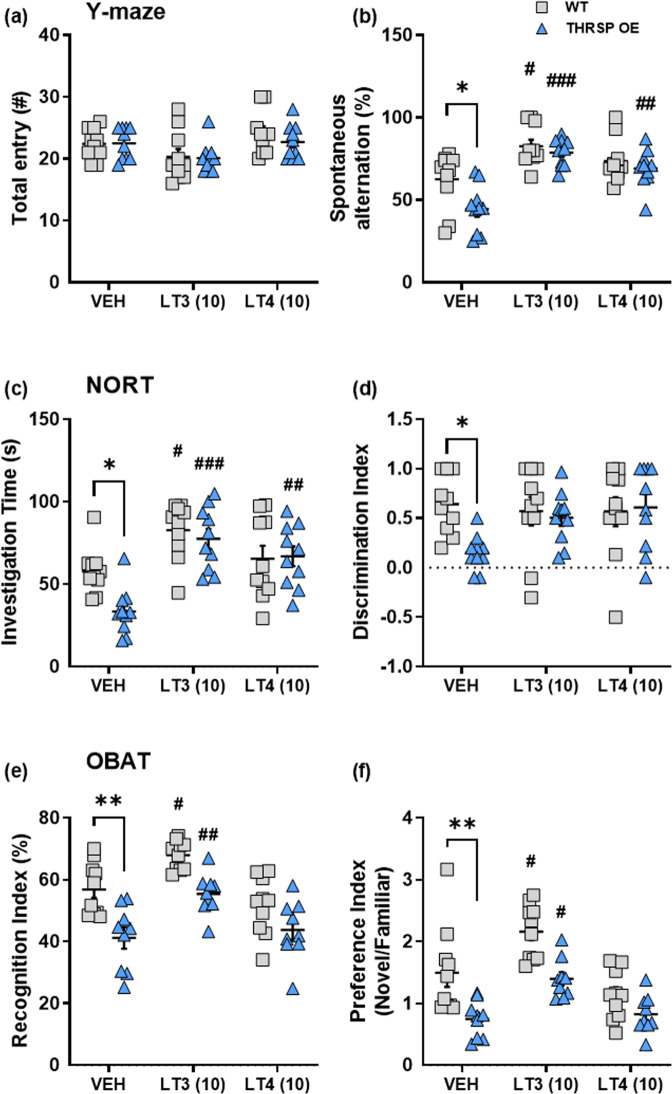
Effects of TH replacement on inattention in THRSP OE mice. Separate cohorts of mice were treated with LT3 and LT4 (10 mg kg^−1^) for seven days. Thirty minutes after the last treatment dose, mice were exposed to the Y-maze test, NORT, and OBAT. The **a** total arm entry, **b** spontaneous alternations, **c** investigation time, **d** discrimination index, **e** recognition index, and **f** preference index were scored. **a** *n* = 10 mice/group; two-way ANOVA; *F*_(1,54)_ = 0.473, *P* = 0.495; **b** *F*_(1,54)_ = 6.89, *P* = 0.011; **c** *F*_(1,54)_ = 3.94, *P* = 0.052; **d** *F*_(1,54)_ = 3.40, *P* = 0.071; **e** *n* = 9–10 mice/group; two-way ANOVA; *F*_(1,51)_ = 29.3, *P* < 0.001; **f** *F*_(1,51)_ = 26.5, *P* < 0.001)). TH replacement improved inattention in THRSP OE mice. Values are presented as the mean ± S.E.M. *P < 0.05 and **P < 0.01 (relative to WT), and ^#^*P* < 0.05, ^##^*P* < 0.01, ^##^*P* < 0.001 (relative to VEH treatment) by two-way ANOVA with Bonferroni’s multiple comparison tests.

We attempted to determine whether TH replacement for seven days can improve ADHD-like behaviors in THRSP OE mice. We observed that LT3 and LT4 at a high dose (10 mg kg^−1^) enhanced the spontaneous alternations in the Y-maze test (Fig. 6b) and the investigation time in the NORT (Fig. 6c), with a slight improvement in the discrimination index (Fig. 6d); however, they were ineffective at lower doses (i.e., 2.5 and 5 mg kg^−1^) (Supplementary Fig. 6b–d), indicating a dose-dependent effect in mice. Furthermore, the dose of LT3 (10 mg kg^−1^) that induced improvements in the Y-maze and NORT was also effective in improving the recognition (Fig. 6e) and preference (Fig. 6f) indices in THRSP OE mice exposed to OBAT. However, LT4 (10 mg kg^−1^) was ineffective, indicating that LT3 is more potent than LT4.

**Fig. 7:**
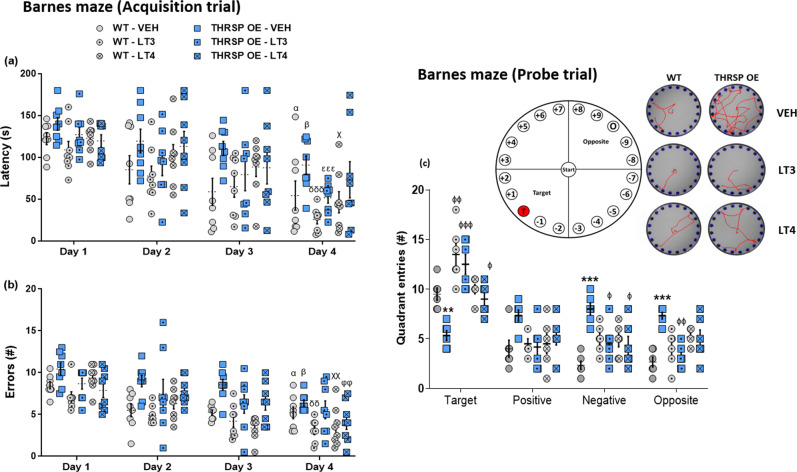
Effects of TH replacement on memory impairment in THRSP OE mice. Another group of mice was treated with LT3 and LT4 (10 mg kg^−1^). Mice started receiving TH two days prior to the start of acquisition trials (or during the habituation stage) and ended on the 5th day when a probe trial was conducted; thus, the total treatment period was seven days. During the acquisition and probe trial stages, the mice were introduced to the task 30 min after the last treatment dose, and the **a** latency and **b** errors in locating the target hole with the “goal” or escape box (*n* = 8 mice/group) and the **c** frequency of visits towards each platform quadrant (i.e., target, positive, opposite, and negative) (*n* = 6 mice/group) were scored; **a** repeated measures two-way ANOVA, *F*_(5,42)_ = 3.50, *P* = 0.010; **b** repeated measures two-way ANOVA, *F*_(5,42)_ = 9.68, *P* < 0.001; **c** two-way ANOVA, *F*_(5,120)_ = 5.42, *P* < 0.001). TH replacement, particularly T3, improved memory impairment in THRSP OE mice. Values are presented as the mean ± S.E.M. ^α(WT-VEH), δ (WT-LT3), χ (WT-LT4), β (THRSP OE), ε (THRSP OE-LT3), φ (THRSP OE-LT4)^*P* < 0.001, *P* < 0.01, by repeated measures two-way ANOVA with Bonferroni’s multiple comparisons relative to the effects of TH on the performance of individual strains (day 1 vs. 4 of acquisition trials). **P < 0.01, ***P < 0.001 (relative to WT) and ^ϕ^*P* < 0.05, ^ϕϕ^*P* < 0.01, ^ϕϕϕ^*P* < 0.05 (relative to VEH treatment) by two-way ANOVA with Bonferroni’s multiple comparison tests.

According to the Barnes maze test, TH replacement improved performance throughout the acquisition trials (Fig. 7a, b), particularly LT3, which effectively reduced the latency time and the number of errors (in locating the escape box), especially in the memory-impaired THRSP OE mice. Notably, LT3 aided the THRSP OE mice in recalling the last location of the escape box, resulting in a higher number of visits to the target quadrant (Fig. 7c) while reducing visitation to the other quadrants (i.e., positive, negative, and opposite).

**Fig. 8:**
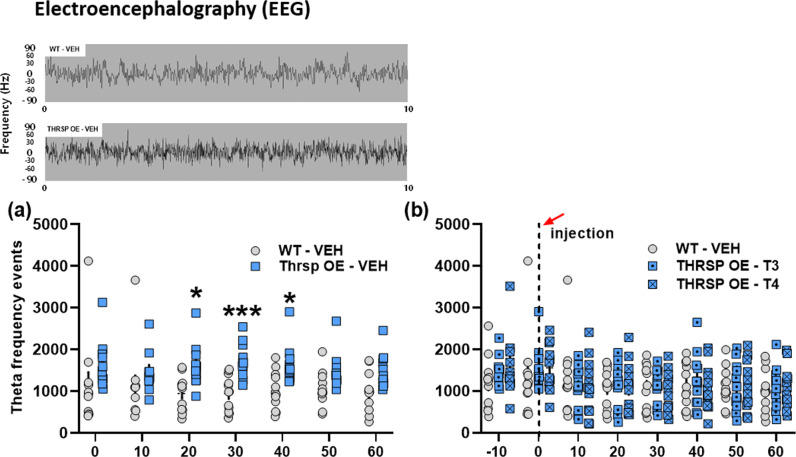
Effects of TH replacement on the excess theta waves in THRSP OE mice. Separate cohorts of mice were treated with LT3 and LT4 (10 mg kg^−1^) for seven days, and the **a** theta waves frequency events and **b** total theta frequencies were recorded and analyzed (*n* = 12 mice/group; **a** repeated measures two-way ANOVA, *F*_(1,22)_ = 11.3, *P* = 0.003; **b** repeated measures two-way ANOVA, *F*_(2,33)_ = 0.105, *P* = 0.901). Values are presented as the mean ± S.E.M. *P < 0.05 and ***P < 0.01 (relative to WT), by repeated measures two-way ANOVA with Bonferroni’s multiple comparison tests. LT3 and LT4 normalized the theta waves in THRSP OE mice similar to WT-VEH.

**Fig. 9:**
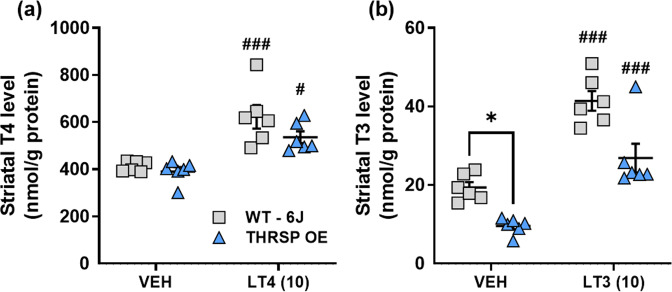
Effects of TH replacement on the striatal T3 levels in THRSP OE mice. **a** T4 and **b** T3 were evaluated following seven days of LT3 and LT4 (10 mg kg^−1^) treatment. On the last day of treatment, the brains were harvested, and the striatum was subjected to TH level analysis (*n* = 6 mice/group; **a** two-way ANOVA, *F*_(1,20)_ = 3.36, *P* = 0.0802; **b** two-way ANOVA, *F*_(1,20)_ = 26.7, *P* < 0.001). TH replacement improved the striatal T3 and T4 levels in THRSP OE mice. Values are presented as the mean ± S.E.M. *P < 0.05 (relative to WT) and ^#^*P* < 0.05. ^###^*P* < 0.001 (relative to VEH treatment), by two-way ANOVA with Bonferroni’s multiple comparison tests.

Furthermore, LT3 and LT4 treatment normalized the theta frequency events in THRSP OE mice similar to WT (Fig. 8b). This result appears to reflect waking theta activity in naive (Fig. 4a) and VEH-treated (Fig. 8a) THRSP OE mice; accounts for the behavioral impairments; and indicates that the enhancement of TH levels (Fig. 9a, b) following TH replacement can normalize this, along with improving ADHD behavior, in these transgenic mice. Overall, we determined that the improvement in attention and other ADHD-like behaviors observed in the THRSP OE mice is associated with the enhancement of TH levels, further supporting the observation that low TH levels are associated with inattention in ADHD^13^.

### TH replacement normalizes altered MCT8 levels in THRSP OE mice

**Fig. 10:**
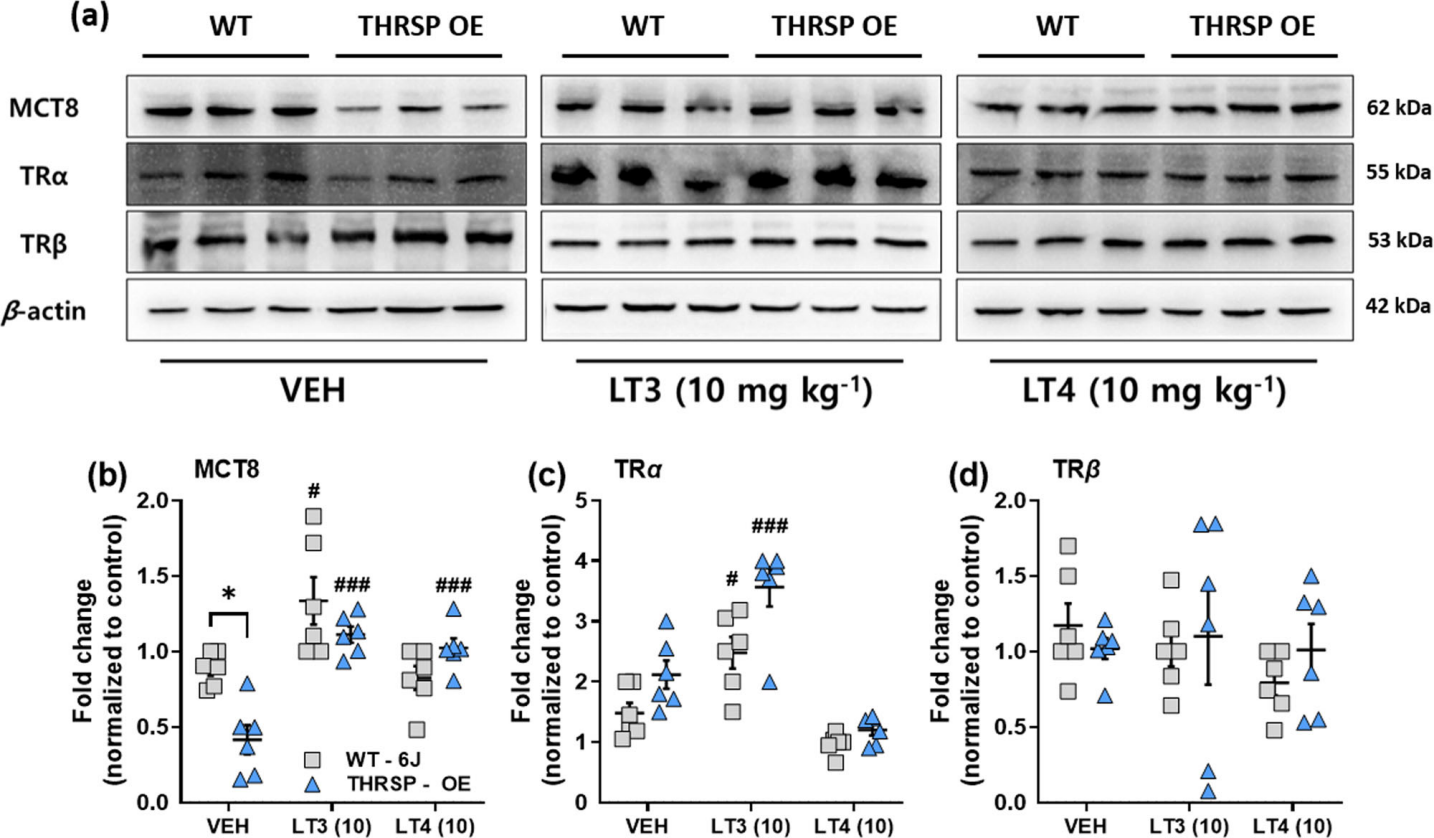
Effects of TH replacement on TH-related proteins in THRSP OE mice. The TH-related proteins were evaluated following 7 days of LT3 and LT4 (10 mg kg^−1^) treatment. On the last day of the treatment, the brains were harvested, and the striatum was subjected to western blot analysis. **a** Representative blots for specific protein targets. **b** MCT8, **c** TRα, and **d** TRβ protein levels (*n* = 6 mice/group; **b** two-way ANOVA, *F*_(1,30)_ = 4.96, *P* = 0.034; **c** two-way ANOVA, *F*_(1,30)_ = 14.4, *P* < 0.001; **d** two-way ANOVA, *F*_(1,30)_ = 0.123, *P* = 0.728). TH replacement improved the striatal MCT8 and TRα levels in THRSP OE mice. Values are presented as the mean ± S.E.M. *P < 0.05 (relative to WT) and ^#^*P* < 0.05. ^##^*P* < 0.01 (relative to VEH treatment), by two-way ANOVA with Bonferroni’s multiple comparison tests.

We evaluated the TH-related factors that may have induced the characteristically low striatal T3 levels in the THRSP OE mice. We observed that the THRSP OE mice exhibited low levels of monocarboxylate transporter 8 (MCT8) (Fig. 10b) and a slightly elevated TRα (Fig. 10c); however, the TRβ levels were normal (Fig. 10d). Interestingly, LT3 and LT4 (10 mg kg^−1^) increased the MCT8 levels in the THRSP OE mice. Also, it improved the MCT8 levels in LT3-treated WT. In addition, LT3 increased the TRα levels in both WT and THRSP OE mice, while no change in the TRβ levels for both strains. MCT8 acts as a brain-specific transporter for TH, particularly the active form of T3^45^. To confirm the role of MCT8 downregulation on attention behavior, we treated the WT mice with a monocarboxylate transporter antagonist “UK-5099”. After 2 weeks of treatment, mice treated with UK-5099 exhibited behavior like that observed in the THRSP OE mice, presenting low spontaneous alternations (Supplementary Fig. 7b) in the Y-maze test and low discrimination index (Supplementary Fig. 7d) in the NORT. According to our findings, the downregulation of MCT8 levels in the striatum may have resulted in the low striatal T3 levels observed in the THRSP OE mice.

## Discussion

In contrast to the ADHD phenotype produced by THRSP overexpression, knockout of THRSP produces no profound effects on mouse behavior. In this regard, Zhu et al.^3^ and Anderson et al.^2^ (2009) previously generated Spot14-knockout and Thrsp-null (Thrsp^tm1cnm^) mice, respectively; however, they did not describe whether these mouse strains showed any neurobehavioral changes^2,3^. Thus, our study is the first to identify that functional deletion of THRSP does not facilitate behavioral alterations, such as those observed in ADHD. These results confirm the role of upregulated THRSP in inattention^19,20^, indicating the potential use of THRSP OE mice as an animal model for the predominantly inattentive subtype of ADHD (ADHD-PI).

Perhaps the most striking observation of our study is the striatal T3 deficiency observed in the THRSP OE mice despite normal levels of circulating TSH, T4, and T3, indicating brain-specific T3 deficiency without hypothyroxinemia (low TH levels in the blood) in this transgenic animal model. It is intriguing how THRSP overexpression induces changes in TH levels. Under normal conditions, the THRSP gene responds directly to changes in TH^1^. However, in this case, we observed the inverse process in which TH levels, particularly striatal T3, was reduced due to  the functional overexpression of THRSP gene in the brain, which suggests that the THRSP gene not only responds to a change in TH status but also induces a change in TH levels, which indicates a synergistic mechanism underlying both processes. In addition, there is a high possibility that changes in TH-related proteins occurred due to THRSP gene overexpression, which may have induced striatal T3 deficiency in the THRSP OE mice.

Moreover, the brain is dependent on TH for normal maturation and function^46^. Conditions such as hypothyroidism may cause stunted brain maturation^47^ along with cognitive impairments^48^. Indeed, our findings show a slightly reduced brain-to-body weight ratio in inattentive and memory-impaired THRSP OE mice. Overall, we observed that THRSP overexpression induces striatal T3 deficiency, inattention, memory impairment, and theta wave alterations in mice.

Interestingly, LT3 or LT4 (10 mg kg^−1^) treatments reversed the ADHD-like behaviors in the THRSP OE mice, demonstrating that TH replacement can ameliorate inattention, as observed in the THRSP OE mice. However, LT3 treatment reduced hyperactivity and impulsivity but not inattention in children with ADHD and GRTH^49^. This discrepancy may result from methodological differences (i.e., subject/sample differences) and thus require further investigation. Also, the effect of the drugs on performances in Y-maze, NORT, OBAT, and Barnes maze demonstrate a long-term therapeutic effect from repeated seven days treatment of THs in mice on the behavioral and biochemical levels. Therefore, the present findings should be considered preliminary evidence of the possible long-term beneficial effects of LT4 and LT3 on inattention and memory impairment. Moreover, TH supplementation has improved the spatial memory and cognitive functions in hypothyroid humans^50^ and rats^51^, which corroborate our findings. Furthermore, the normalization of theta waves in mice following TH replacement corresponds to the normalization of EEG frequencies following TH replacements in humans with hypothyroidism^52^, indicating that TH influences EEG frequencies.

T3 enters neural target cells primarily through MCT8, a brain-specific TH transporter^53^. Mutations in the MCT8 gene in Xq13 induce TH abnormalities and severe neurodevelopmental disorders in humans and rodents, commonly identified as Allan-Herndon-Dudley (AHD) syndrome^53^. However, with our present results, the manifestations observed in THRSP OE mice differ from AHD, which presents muscular spasticity and severe psychomotor delays that were characteristically absent in THRSP OE mice (as observed by similar motor behavior/activity with that of WT). In the present study, we observed low striatal MCT8 levels in the THRSP OE mice, confirming that the reduction of striatal T3 is a consequence of low MCT8, impairs brain TH uptake^54^. Also, functional deletion of MCT8 induces hyperthyroxinemia and upregulates Thrsp (Spot14) mRNA levels in mct8 KO mice^55^, indicating an inverse relationship between these genes. In addition, TH replacement normalized MCT8 levels in the THRSP OE mice, like in the WT mice. These results corroborate the subsequent improvement in inattention following TH treatment.

Furthermore, LT3 increased striatal TRα; however, it did not change TRβ protein levels in THRSP OE and WT mice. In the THRSP OE mice, this event may manifest a state of compensatory hypothyroidism^56^. However, it is intriguing that TRβ was unaffected by this event. This finding may be owing to the overlapping expression and function of these nuclear receptors, as TRβ is responsible for mediating most actions of T3 in the liver^57^, whereas TRα mediates TH effects, particularly in the brain^58,59^. However, further studies are needed to confirm these hypotheses.

The exact mechanism by which the overexpression, but not the knockout of THRSP gene, induces inattention and memory impairment is still unclear. However, since THRSP overexpression induces alteration in T3 levels in the brain and TH replacement improves these, the hormonal system appears to be highly involved. Given this, a previous in situ hybridization (ISH)-based study demonstrated that THRSP (Spot14)-expressing cells constitute neural stem progenitor cells (NSPCs)^6,60^ confined in the TH-dependent neurogenic areas of the adult brain^61^, the subgranular zone (SGZ) of the dentate gyrus (DG) in the hippocampus, and the subventricular zone (SVZ) lining the lateral ventricles (LV) of the cerebral cortex. NSPCs play a pivotal role in ensuring lifelong neurogenesis in the mammalian brain, which is necessary for learning, memory consolidation, and cognitive functions^62,63^. Indeed, alterations in the production, development, and regulation of new neurons in the neurogenic regions of the brain induce neurodegeneration and psychiatric disorders, including ADHD^64,65^.

Interestingly, retrovirus-mediated overexpression of THRSP reduced the proliferation of NSPCs, whereas its knockdown led to proliferative NSPCs, indicating that THRSP is necessary for modulating NSPCs necessary for neurogenesis. We speculate that the functional deletion and overexpression of THRSP in mice influence the production of NSPCs, which are highly responsive to TH, which may be involved in the inattention and memory impairments or the lack thereof in these transgenic mice. Additionally, there may exist other possibilities that could explain our findings.

While this study has strengths, it also has a limitation that needs clarification, as the present study focused only on male mice. Studies show clear sex differences in ADHD prevalence, subtypes, and specific DSM-*5* ADHD symptoms; males are likely to be diagnosed with ADHD than females^66–68^. Nonetheless, it would be valuable to identify sex differences, considering the potential variations in TH between male and female mice.

### Conclusions

**Fig. 11:**
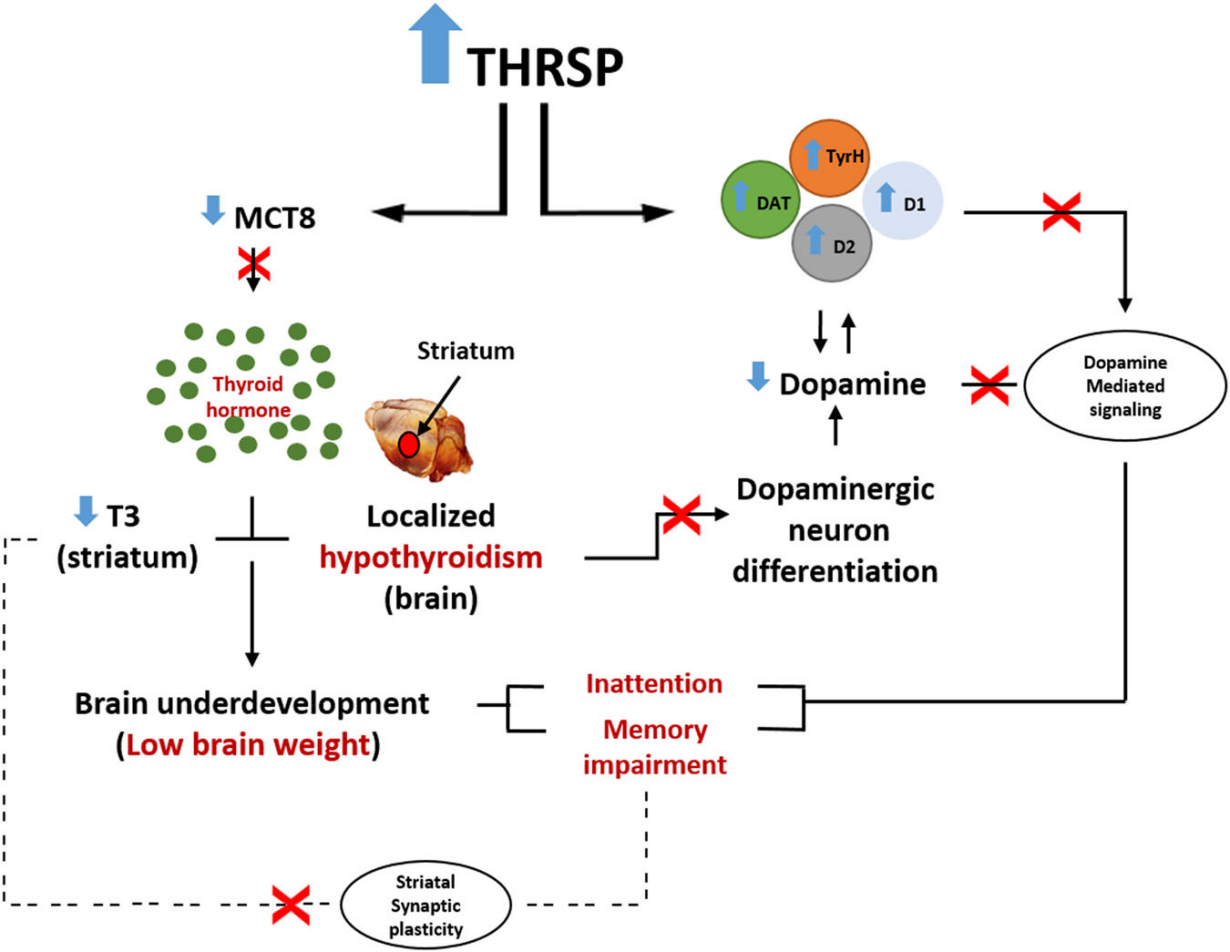
Scheme for the proposed mechanism underlying THRSP overexpression-induced inattention and memory impairment in mice. Our previous and present findings show that a shared dopaminergic and thyroid hormone mechanism, respectively, is involved in the deranged neurobehavioral patterns observed in THRSP OE mice. THRSP (Thyroid hormone-responsive protein); MCT8 (Monocarboxylate transporter 8); T3 (Triiodothyronine); TyrH (Tyrosine Hydroxylase); DAT (Dopamine transporter); D1 (Dopamine D1 receptors); D2 (Dopamine D2 receptors).

The present study revealed that striatal T3 deficiency induces inattention and other ADHD features observed in THRSP OE mice. Our study further supports the involvement of upregulated THRSP in ADHD and indicates that THRSP OE mice can serve as a potential animal model for ADHD-PI. However, although THRSP OE mice characterize the ADHD-PI subtype, the novelty of THRSP in the domain of ADHD research still presents some challenges. Particularly, the role of THRSP in ADHD pathology is not yet fully understood, and the function of this gene and the mechanisms whereby its overexpression causes inattention and other ADHD-like phenotypes remain baffling. Our previous and present findings do, however, tend to indicate that shared dopaminergic and thyroid function alterations (Fig. 11) may be responsible for these behaviors, given that the dopaminergic and thyroid system is directly involved in the regulation of attention and cognitive behaviors. Also, given that THRSP is a TH-related protein and that TH and dopamine have tyrosine as a common basic unit, it is conceivable that the functional overexpression of THRSP influences the dopaminergic system in THRSP OE mice. However, further studies are required. Nonetheless, THRSP OE mice may be used in future studies to advance our understanding of ADHD etiology and screen novel compounds for ADHD treatment, particularly in the ADHD-PI subtype. Overall, our findings show the *face*, *predictive*, and *construct validity* of THRSP OE mice in modeling the ADHD-PI subtype.

## Methods

### Animals

THRSP-overexpressing (THRSP OE) (MGI:6259716; Tg^(Eno2-Thrsp)#Cheo^) mice were produced following standard procedures^20^. In brief, we produced the first-generation THRSP OE mice from an intercross between THRSP OE male founders (Uimyung Research Institute for Neuroscience) and C57BL6 female mice (Hanlim Animal Laboratory Co. (Hwasung, Korea). Currently, we are using the tenth-generation back-crossed C57BL6-THSRP OE mice. Also, we produced the first-generation inbred THRSP Hetero mice from an intercross between heterozygous Thrsp^em1cyagen^ (Serial no.: KOCMP-21835-Thrsp; NCBI ID: 21835) male founders from Cyagen Biosciences (Santa Clara, CA, USA) and C57BL6 female mice from Hanlim Animal Laboratory Co. (Hwasung, Korea). After four breeding cycles, we produced an adequate number of C57BL6-THRSP heteroback-crossed male and female mice, then matched to produce THRSP KO mice. The THRSP transgenic (TG) (i.e., OE, Hetero, and KO) neonates were viable and did not exhibit gross physical deformities or abnormalities. At 3 weeks of age, before the experiment, genotyping (DNA electrophoresis) was conducted using appropriate PCR conditions and primers to identify TG mouse strains and separate them from their non-TG littermates. THRSP OE (Forward primer [F]: 5′-CCATGCAAGTGCTAACGAAA-3′; Reverse primer [R]: 5′-CACTCAGAGGGAGACGGAAG-3′; PCR Conditions: 94 °C, 5 min → (94 °C, 1 min → 58 °C, 1 min → 72 °C, 1 min)  × 35 cycles → 72 °C, 10 min → 4 °C, ∞). THRSP Hetero/KO (F1: 5′-CTGGCGCCTTAATTGATTTTGGTT-3′; R1: 5′-GCTGCTCTATACCATGACCTGTTT-3′; F2: 5′-CACTAATTGTCCAAGTAGGTCTCCA-3′; R1: 5′-GCTGCTCTATACCATGACCTGTTT-3′; Conditions: 94 °C, 3 min → (94 °C, 30 s → 60 °C, 35 s → 72 °C, 35 s) × 35 cycles → 72 °C, 5 min → 4 °C, ∞). To preserve the TG mouse lines and maintain a significant number of samples for our experiments, female mice of each strain followed continuous breeding, and male mice were utilized for all experiments. We maintained a grouping of four mice per cage housed in a temperature- and humidity-controlled environment (temperature, 22 ± 2 °C; relative humidity, 55% ± 5%; 12/12 h light/dark (07:00–19:00 h light) cycle. All efforts were carried out to minimize the number of experimental animals. Behavioral evaluations commenced when the mice reached four weeks of age, based on previous studies evaluating animal models for ADHD behaviors during this period^19,20,24,69,70^. We utilized the male C57BL6 (identified as WT) mice as normal controls for the entire study. We carried out mice handling for 5 min for seven days and placed on the experimental room 1H before the experiment. We performed the standard animal care and procedures following the Principles of Laboratory Animal Care (NIH Publication No. 85-23, revised 1985), the Animal Ethics Review Board of Sahmyook University, South Korea (SYUIACUC2020-010), and in compliance with the ARRIVE guidelines^71^ recommended by *Communications Biology*.

### Drugs

LT3 [T2877; CAS no: 6893-02-3] and LT4 [T2376; CAS no: 51-48-9]) were purchased from Sigma-Aldrich (St. Louis, MO, USA) and dissolved in appropriate doses (2.5, 5, and 10 mg kg^−1^) in 0.7% dimethyl sulfoxide (DMSO) and 1% Tween-80 in normal saline (vehicle; VEH). The doses used in the study were based on previously published data^72^ and followed the drug safety assessment strategies^73^ by establishing a safe starting dose level, maximal tolerable dose, and exposure to or frequency of drug treatment. To assess the involvement of MCT8 in the inattention in THRSP OE mice (Supplementary Fig. 7), MCT antagonist UK 5099 was injected in 3 weeks-old C57BL6 male mice with a dose of 5 mg kg^−1^ for 2 weeks. We then expose mice to Y-maze and NORT thirty minutes following the last treatment. UK 5099 was purchased from Sigma-Aldrich (St. Louis, MO, USA) and dissolved in saline.

### Behavioral tests

#### Y-maze test

We evaluated inattention using the Y-maze test^20^. Each mouse was placed in one of the Y-maze (45 × 10 × 20 cm) arms and allowed to explore freely for 10 min. Ethovison XT (RRID: SCR_000441; Noldus, Netherlands) recorded the movement in mice. The study defines an “arm entry” as the entry of all four paws (mice) into an arm and the “alternation behavior” (actual alternations) as a consecutive entry into three arms. The percentage of spontaneous alternation is calculated as the ratio of actual alternations to the maximum number of alternations (total number of arm entries minus two) multiplied by 100 (% alternation = [(number of alternations)/(total arm entries − 2)] × 100). A separate group of mice (i.e., THRSP OE, WT) was injected with LT3 and LT4 (2.5, 5, and 10 mg kg^−1^) 30 min before the start of each experiment for assessing the pharmacological effects of these drugs on behavior.

#### Novel-object recognition test (NORT)

The novel-object recognition test (NORT) was performed to evaluate attention in mice^20^. The test consisted of three phases: habituation, familiarization, and discrimination. Each mouse was placed in an open-field box for 10 min/day for two consecutive days (habituation phase). The movement was recorded using Ethovison XT (RRID: SCR_000441; Noldus, Netherlands). Following the last day of habituation, two identical objects (wooden boxes) (A1 and A2) were placed in opposite and equidistant locations, and each mouse was allowed to explore and familiarize with the objects for 10 min (familiarization phase). The mice were subsequently returned to their home cage and allowed to stay for 30 min before being reintroduced into the arena for the discrimination phase. In this phase, objects A1 and A2 were replaced by an object identical to the familiar object (A3) and a novel object (B). We determined the total investigation time (A1 + A2) in the familiarization phase and the discrimination index (B-A3/B + A3) during the discrimination phase. The discrimination index ranged between +1 and −1: a positive score indicated more time spent with the novel object, while a negative score indicated more time spent with the familiar object; zero indicated a null preference. A separate cohort of mice (i.e., THRSP OE, WT) was injected with LT3 and LT4 (10 mg·kg^−1^) 30 min before the experiment.

#### Object-based attention test (OBAT)

We utilized the OBAT as a valid attention test previously used in an ADHD model^27–29^. Each mouse was habituated in two empty open-field boxes (labeled as exploration chamber [EC] and test chamber [TC]) for 10 min/day for two consecutive days. Correspondingly, mice were also handled for 5 min following each habituation session. Each mouse was placed in the EC containing five objects of the same size but of different shapes during acquisition. The five objects were of the following shapes: circle, square, cylindrical, pentagon, and star-shaped. Then, each mouse was placed in the TC containing a familiar (circle) and a novel (triangle) object and allowed for another 10 min of object exploration. The positions for each object in the EC and TC were fixed throughout the study. We used the formula: TN/(TF + TN) × 100 to calculate the recognition index in mice, where TF and TN are the time spent exploring the objects in the TC. Also, we have derived another parameter identified as the ‘preference index’ calculated using the formula: TN/TF, where time spent exploring the novel object is divided into the time spent in the familiar object. Object exploration was recorded using Ethovison XT (RRID: SCR_000441; Noldus, Netherlands). Furthermore, separate cohorts were treated with LT3 and LT4 (10 mg kg^−1^) or VEH for seven days and then exposed to the OBAT following 30 min from the last treatment.

#### Barnes maze test

The procedure was a modification of a previously reported method^74,75^, comprising acquisition and probe trials. The maze consisted of a white Plexiglas circular platform (∅: 92 cm, height: 105 cm) with 20 equally spaced holes (∅: 5 cm) located 3 cm from the border. In this open environment, mice naturally seek a dark enclosed surrounding place, provided by a black goal box (6 × 12 × 6 cm) located beneath one of the holes. Visual cues are placed around the maze, which acts as spatial cues. Each trial began by placing the mouse in a black starting cylinder (∅: 8 cm, height: 12.5 cm) at the center of the platform, removed after 30 s, allowing mice to explore the maze freely. Mice were habituated to the platform two days before the start of the acquisition trials. The spatial acquisition was organized in 2 trials (Days 1–4). Each trial consisted of two 3 min trials, with 30 min inter-trial intervals during which animals were allowed to return to their home cage for rest. Mice that failed to find the target box within 3 min were gently guided to their location. For these mice, 180 s was recorded as the escape latency. All animals remained in the target box for 60 s after entry. The study defines ‘latency’ as the time taken for the mice to enter the goal box. Errors were identified as the number of visits to other holes that did not contain the goal box. Ethovison XT (RRID: SCR_000441; Noldus, Netherlands) was used to record the latency and movement of each mouse. On Day 5, short-term reference memory was evaluated in a probe trial (180 s), during which the target box was removed. The mice were allowed to explore the maze and visit the target hole. The adjacent holes were divided into four quadrants (e.g., target, positive, negative, and opposite). The distribution of visits among all holes and the time spent in each quadrant were recorded. Separate cohorts of mice (i.e., THRSP OE, WT) were injected with LT3 and LT4 (10 mg kg^−1^) for seven days before the experiment.

#### Open field test

The open-field test was used to evaluate the locomotor activity of mice^24^. Mice locomotor activity was assessed in a square black Plexiglas container with an open-field arena (42 × 42 × 42 cm^3^). A computer system, Ethovison XT (RRID: SCR_000441; Noldus, Netherlands), was utilized to record the distance moved (cm) and movement duration (s) for each mouse. Mice (*n* = 10) were gently placed in the center of the open-field arena and allowed to explore for 30 min for five consecutive days. The first two days served as a habituation period that eliminated the bias of novelty in the “open-field” (unrecorded). On the 3rd (D1), 4th (D2), and 5th (D3) days, locomotor activity for each strain was recorded.

#### Cliff-avoidance test

The cliff-avoidance test was utilized to assess impulsivity in mice^76^. A round Plexiglas platform (diameter, 20 cm; thickness, 1 cm) on both ends, supported by a cylindrical-shaped Plexiglas rod (height, 50 cm) in between, was used. During each test, mice (*n* = 10) were placed at the center of the platform and were observed for 10 min. The latency of the first fall (the actual time the subject fell from the platform) and falling frequencies (number of falls) were recorded using a computer system Ethovison XT (RRID: SCR_000441; Noldus, Netherlands).

#### Elevated-plus maze test

This test was used to assess anxiety in mice^77,78^. The plus-maze consisted of four arms: two open arms (30 × 6 cm^2^) and two closed arms (30 × 6 cm^2^) enclosed by 20-cm-high walls with a delimited central area of 6 × 6 cm. The entire maze was elevated to a height of 50 cm above the floor. We utilized indirect lighting to prevent the formation of hard shadows, which can be an area of preference for mice. We placed each mouse (*n* = 10) at the center of the maze facing one of the open arms during the test. Entry into an arm was marked when the mouse places all four paws over the line marking on that area. The number of entries and the time spent in the open arms were recorded using Ethovison XT (RRID: SCR_000441; Noldus, Netherlands) during a 10-min test period. We calculated the percentage of open arm entries (100 × open/total entries) and the percentage of time spent in the open arms of the maze for each mouse.

#### Rota-rod test

The ability to maintain motor balance and coordination^79^ were assessed using a rotating rod (Ugo Basile, Varese, Italy) at 36 rotations per minute (rpm). The experiment was conducted for five consecutive days. Two days prior to the actual recording of the experiment, mice were trained to run in the rotating rod for 5 min. Then, on the actual day of the experiment, on the 3rd (D1), 4th (D2), and 5th (D3) day, mice (*n* = 10) were placed on the rotating rod for 10 min. The latency to first fall and falling frequencies were recorded.

### Electroencephalography (EEG)

EEG was performed to assess theta waves in mice according to our previous study^80,81^. Briefly, a tethered, three-channel system head-mount (8200-K3-iS/iSE; Pinnacle Technology, Inc., Lawrence, KS) was implanted, secured with two stainless steel screws positioned frontally (A/P: −1.0 mm; M/L, −1.5 mm) and posteriorly (A/P: −1.0 mm; M/L, ±1.5 mm), and fixed with cement. All efforts were made to minimize animal suffering. Mice were given a 7-day recovery period before the EEG recording, with available water and food *ad libitum*. Mice were habituated to the EEG apparatus for 2 h over two consecutive days (unrecorded). On the day of the experiment, a 1H EEG recording was performed. In addition, a separate EEG recording was conducted in THRSP OE and WT mice after seven days of treatment with either LT3 or LT4 (10 mg kg^−1^) for assessing the effects of TH replacement on brain wave activity in the mice.

### Blood and organ tissue collection

Mice were sacrificed following all behavioral tests. Whole blood was extracted from naive, VEH-treated, and drug-treated mice through cardiac puncture^82^; collected in a 1.5 ml Eppendorf tube; and centrifuged for 15 min at 2200–2500 rpm. The plasma was then carefully pipetted and stored at −80 °C until the assay. Body organs (i.e., the brain, heart, lungs, liver, spleen, kidneys, and testes) were isolated and weighed. The brains were rapidly harvested and placed in an ice-cold saline buffer. Brain extraction was performed to prevent brain damage. The striatum, a region of interest, was isolated and stored at −80 °C until analysis.

### Enzyme-linked immunosorbent assay (ELISA)

ELISA was performed for measuring important targets for evaluating TH function in mice, including TSH, T4, and T3.

#### TSH, T4, and T3 ELISA

TSH, T4, and T3 (#LS-F5125, #LS-F10014, #LS-F10016; LSBio, Seattle, WA) were measured by ELISA using either plasma or brain (striatum) samples from mice. In brief, standards or samples (protein lysates used in western blotting) were added to the designated wells (in duplicates). A biotinylated standard was added to each well and incubated for 60 min at RT. After washing three times, horseradish peroxidase (HRP)-avidin conjugate was added and incubated for 30 min at RT, followed by three washes. Substrates A and B were added to each well (including blank) and incubated for 15 min at RT.

#### Reading/Measurement

Stop solutions for TSH, T4, and T3 ELISA were added, and the absorbance was measured at 450 nm (SoftMax Pro Data Acquisition and Analysis Software, RRID: SCR_014240; Molecular Devices, San Jose, CA, USA). All measurements were performed in duplicates.

### Western blotting

Western blotting was performed to identify the THRSP and the TH-related proteins (i.e., MCT8, TRα, and TRβ) levels to assess their involvement in the changes observed in THRSP OE mice. The protocols for western blotting were in accordance with those used in our previous studies^20,80^. Briefly, 30 μg of protein lysates were loaded and electrophoresed on 10% sodium dodecyl sulfate and polyacrylamide gels before transferring onto nitrocellulose membranes. Membranes were blocked with 5% skimmed milk in Tris-buffered saline with Tween-20 (TBST) for 1 h and incubated overnight with the following primary antibodies: rabbit polyclonal anti-THRSP 1/1000 (Thermo Fisher Scientific Cat# PA5-77177, RRID: AB_2720904), rabbit polyclonal anti-MCT8 1/1000 (Fitzgerald Cat#70R-50398), rabbit polyclonal anti-TRα 1/250 (Bioss Cat# bs-6221R, RRID: AB_11072581), and mouse monoclonal anti-TRβ 1/1000 (Thermo Fisher Scientific Cat# MA1-216, RRID: AB_2287303). The standard loading control was included using a mouse monoclonal anti-β-actin 1/5000 antibody (Sigma-Aldrich; A5441). Subsequently, the blots were washed in TBST and incubated with appropriate HRP-conjugated secondary antibodies (RRID: AB_11125142 and RRID: AB_11125547; Bio-Rad Laboratories, Hercules, California, United States) for 1 h. After three final washes with TBST, the blots were visualized using enhanced chemiluminescence (Clarity Western ECL; Bio-Rad Laboratories) in a ChemiDoc Imaging System (Image Lab Software, RRID: SCR_014210; Bio-Rad Laboratories). Total protein levels were normalized to β-actin 1/5000 (mouse monoclonal; Sigma-Aldrich Cat# A5441, RRID: AB_476744).

### RNA extraction and quantitative reverse transcription-polymerase chain reaction (qRT-PCR)

Total RNA was isolated using Trizol reagent (Invitrogen, Carlsbad, CA, USA). A Hybrid-RTM Kit (Geneall Biotechnology, Seoul, Korea) was used for further RNA purification. The total RNA concentration was determined with a Colibri Microvolume Spectrometer (Titertek-Berthold, Pforzheim, Germany).

The qRT-PCR was utilized to measure the mRNA expression levels of iodothyronine deiodinases (e.g., *dio1*, *dio2*, *dio3*) and the thyroid hormone transporter organic anion transporting polypeptide 1C1 (*oatp1c1*), following our previous published paper^83^. One microgram (ug) of total RNA was reversely transcribed into cDNA using AccuPower CycleScript RT Premix (Bioneer, Seoul, Korea). The cDNA amplification was performed with custom-made sequence-specific primers (Cosmogenetech, Seoul, Korea) (*dio1*, F: 5′-GCTGAAGCGGCTTGTGATATT-3′, R: 5′-GTTGTCAGGGGCGAATCGG-3′; *dio2*, F: 5′-AATTATGCCTCGGAGAAGACCG-3′, R: 5′-GGCAGTTGCCTAGTGAAAGGT-3′; *dio3*, F: 5′-GTTTTTGGCTTGCTCTCAGG-3′, R: 5′-CAACAAGTCCGAGCTGTGAA-3′; *oatp1c1*, F: 5′-GCAAATGTTCAGACTCAAAATGGG-3′, R: 5′-ATATAATGTTCTTTCCACTCCGGC-3′) and was detected with SYBR Green (Solgent, Korea). The qRT-PCR analysis was performed in triplicate, and the values were normalized to the mRNA levels of the housekeeping gene glyceraldehyde 3-phosphate dehydrogenase (GAPDH), F: 5′-AGGTCGGTGTGAACGGATTTG-3′, R: 5′-TGTAGACCATGTAGTTGAGGTCA-3′]. Relative expression levels were calculated using the 2^−ΔΔCt^ method.

### Statistics and reproducibility

Statistical analyses were performed using GraphPad Prism v9 (RRID: SCR_002798). For graphical purposes, data are presented as the mean ± standard error (SEM), and all statistical analyses were conducted on raw data tested for normal (Gaussian) distribution using D’Agostino-Pearson omnibus. In all available figures, the animal numbers and recorded data points were indicated. Results were analyzed using either a *t*-test, one-way or two-way analysis of variance (ANOVA) with or without repeated measures (RM), followed by Dunnett’s multiple comparisons test. A level of probability of *P* ≤ 0.05 was defined as the threshold for statistical significance. Experiments were replicated at least three times. Detailed statistical analysis (e.g., genotype, treatment, and interaction effects) and raw datasets are provided in Supplementary data 1.

### Reporting summary

Further information on research design is available in the Nature Research Reporting Summary linked to this article.

## Supplementary information

Peer Review File

Supplementary Information

Description of Additional Supplementary Files

Supplementary Data 1

Reporting summary

## Data Availability

The authors declare that the data supporting the findings of this study are available within the paper and its Supplementary information files.
