# Supplementary material for: Low striatal T3 is implicated in inattention and memory impairment in an ADHD mouse model overexpressing thyroid hormone-responsive protein
Source: Commun Biol. 2021 Sep 20;4:1101. doi: 10.1038/s42003-021-02633-w (PMC8452653; doi:10.1038/s42003-021-02633-w)
Supplement: Supplementary file 2 — Supplementary Information [file 42003_2021_2633_MOESM2_ESM.pdf]

Low striatal T3 is implicated in inattention and memory impairment in an ADHD model overexpressing thyroid hormone-responsive protein

Raly James Perez Custodio, Mikyung Kim, Leandro Val Sayson, Hyun Jun Lee,
Darlene Mae Ortiz, Bung Nyun Kim, Hee Jin Kim and Jae Hoon Cheong

Supplementary information (available in this document)

- Supplementary Figures 1-7 (and corresponding figure legends)
- Untruncated western blots used in Figure 1c, Figure 10a, and Supplementary Figure 1a

Supplementary Figure 1

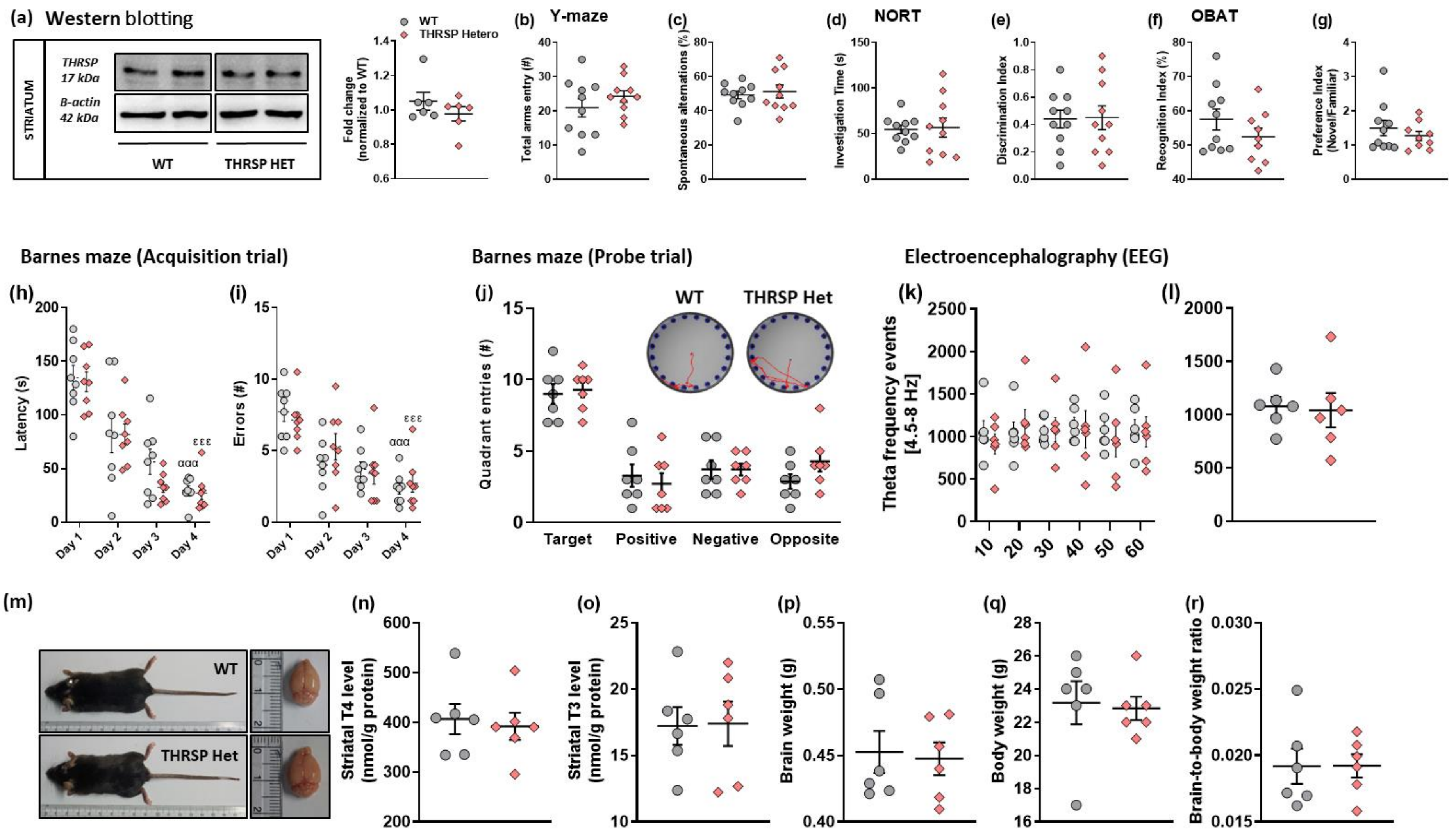

Supplementary Figure 1. The THRSP Hetero mice. (**a**) Western blot using striatal tissue samples show the expression of THRSP protein levels in THRSP Hetero mice (*n* = 6 mice/group; (**a**) two-tailed student *t*-test [t=1.16, df=5]). No significant difference in the expression striatal THRSP protein levels compared to WT. Subsequently, behaviors were evaluated. THRSP Hetero mice were exposed in the Y-maze apparatus for 10 min and the **(b)** total arm entry and **(c)** spontaneous alternations were recorded (*n* = 10 mice/group, two-tailed student *t*-test; **(b)** t=1.71, df=9; **(c)** t=0.502, df=9). Afterwards, mice were subjected to the NORT, where the **(d)** investigation time and **(e)** discrimination index were scored (*n* = 10 mice/group, two-tailed student *t*-test; **(d)** t=0.158, df=9; **(e)** t=0.0836, df=9). No significant difference between groups. Then, another cohort were evaluated in OBA test, where the **(f)** recognition index and **(g)** object preference were scored (*n* = 9-10 mice/group, two-tailed student *t*-test; **(f)** [t=1.25, df=17]; **(g)** [t=0.842, df=17]). No significant difference between groups. Moreover, mice were subjected to the Barnes maze test for 4 days of acquisition trials for assessing the (**h**) latency and (**i**) errors in identifying the target hole with the "goal" or escape box (*n* = 8 mice/group, repeated measures two-way ANOVA; **(h)** $F_{(1.89, 26.4)} = 44.7$, $P < 0.001$; **(i)** $F_{(2.00, 28.0)} = 24.9$, $P < 0.001$). Then, the short-term memory was assessed on the 5th day in a probe trial, in which individual mice were assessed based on (**j**) frequency of visits towards each platform quadrant (i.e., target, positive, opposite, and negative) (*n* = 7 mice/group; **(j)** two-way ANOVA, $F_{(1, 48)} = 0.399$, $P = 0.531$). Latency and errors improved throughout the acquisition trials for both groups. Additionally, they spent more time in the target quadrant with few visitations to other quadrants suggesting an intact memory in THRSP Hetero mice. Values are presented as the mean ± S.E.M. $^{\alpha\,(WT),\,\varepsilon\,(THRSP\,Hetero)}$ $P < 0.001$ according to repeated measures two-way ANOVA with Bonferroni's multiple comparison tests relative to the performance of individual strains during day 1 vs 4 of acquisition trials. Following all behavioral tests, mice were subjected to EEG, and the (**k**) theta waves frequency events and (**l**) total theta frequencies were analyzed (*n* = 6 mice/group; **(k)** repeated measures two-way ANOVA, $F_{(1, 10)} = 0.0395$, $P = 0.846$; **(l)** two-tailed student *t*-test [t=0.234, df=5]). No significant difference in the theta waves between groups. Furthermore, thyroid hormone levels were evaluated in THRSP Hetero mice. (**m**) A snapshot of mice and their brains. The striatal **t**hyroid hormones **(n)** T4 and (**o**) T3 and the (**p**) brain weight, (**q**) body weight, and (**r**) brain-to-body weight ratio were measured (*n* = 6 mice/group, two-tailed student *t*-test; **(n)** t=0.339, df=5; **(o)** t=0.0907, df=5; **(p)** t=0.354, df=5; **(q)** t=0.241, df=5; **(r)** t=0.0228, df=5). No difference between groups were observed. Values are presented as the mean ± S.E.M.

Supplementary Figure 2

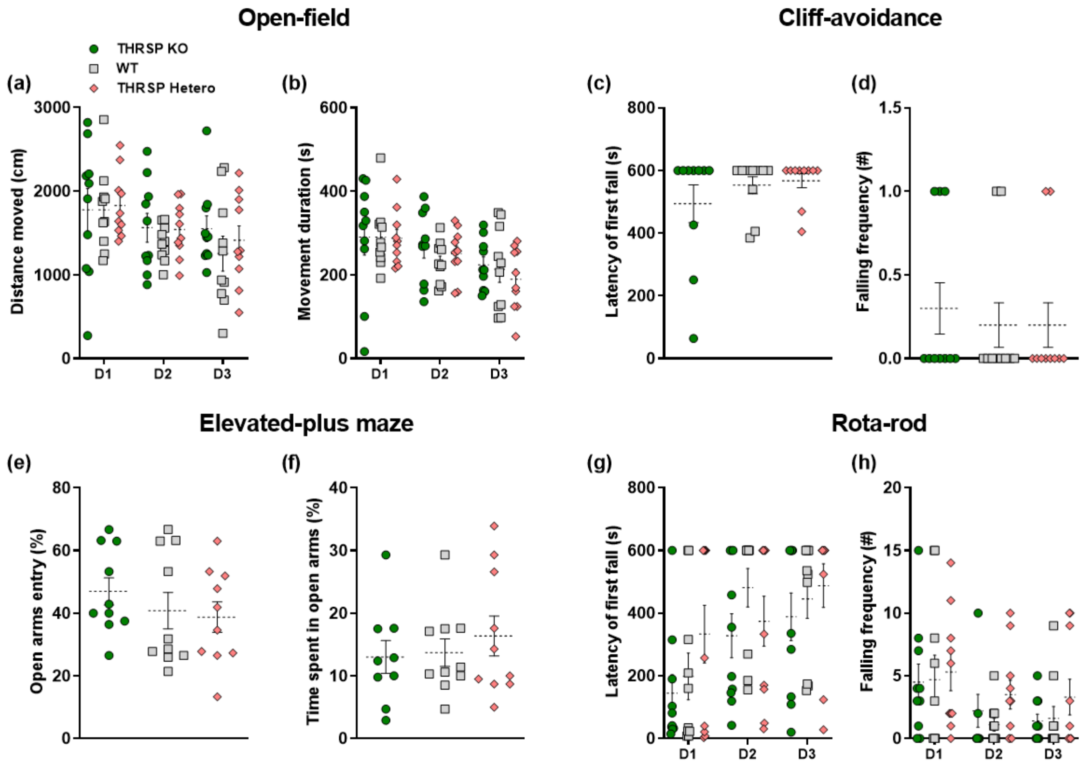

Supplementary Figure 2. Effects of THRSP gene deletion (knockout; KO) on the hyperactivity, impulsivity, and other behaviors in mice. Hyperactivity and impulsivity were evaluated using the (**a**) distance moved and (**b**) movement duration during the open-field test, and the (**c**) latency of first fall and (**d**) falling frequencies in the cliff-avoidance test (*n* = 10 mice/group; **(a)** repeated measures two-way ANOVA, $F_{(2, 27)} = 0.440$, $P = 0.648$; **(b)** repeated measures two-way ANOVA, $F_{(2, 27)} = 0.273$, $P = 0.763$; **(c)** one-way ANOVA, $F_{(2, 27)} = 0.932$, $P = 0.406$; **(d)** one-way ANOVA, $F_{(2, 27)} = 0.170$, $P = 0.845$). Correspondingly, the anxiety and motor balance were evaluated using (**e**) per cent open arms entry and (**f**) per cent time spent in open arms in the elevated-plus maze, and the (**g**) latency of first fall and (**h**) falling frequencies during the rota-rod test (*n* = 10 mice/group; **(e)** one-way ANOVA, $F_{(2, 27)} = 0.719$, $P = 0.496$; **(f)** one-way ANOVA, $F_{(2, 26)} = 0.426$, $P = 0.657$; **(g)** repeated measures two-way ANOVA, $F_{(2, 27)} = 1.10$, $P = 0.346$; **(h)** repeated measures two-way ANOVA, $F_{(2, 27)} = 0.689$, $P = 0.510$). THRSP KO and Hetero mice are not hyperactive nor impulsive. Additionally, they did not show signs of anxiety and motor impairment. Values are presented as the mean ± S.E.M.

Supplementary Figure 3

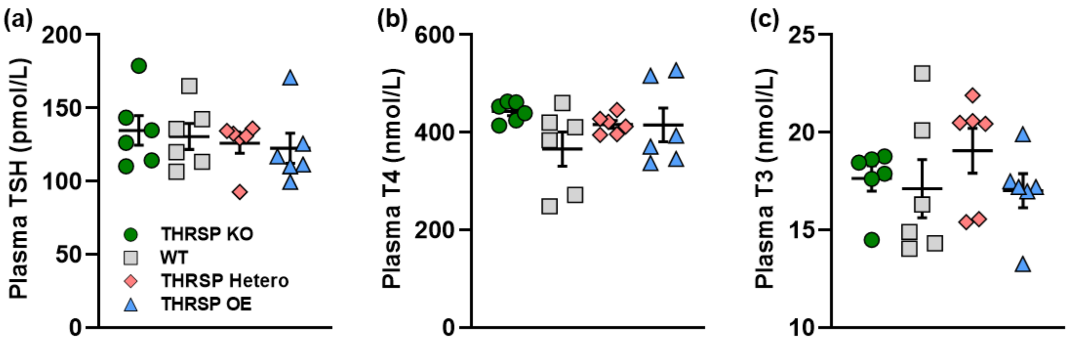

Supplementary Figure 3. The effects of overexpression and functional deletion of THRSP on plasma TSH, T4, and T3 levels in mice. Thyroid hormone functions in non-treated (naïve) mice were evaluated using enzyme-linked immunosorbent assay (ELISA). The mice were anesthetized and sacrificed for blood collection, and the plasma (a) TSH, (b) T4, and (c) T3 was assayed (n = 6 mice/group; (a) one-way ANOVA, $F_{(3, 20)} = 0.336$, $P = 0.799$; (b) one-way ANOVA, $F_{(3, 20)} = 1.61$, $P = 0.218$; (c) one-way ANOVA, $F_{(3, 20)} = 0.753$, $P = 0.534$). No changes were observed between groups. Values are presented as the mean ± S.E.M.

Supplementary Figure 4

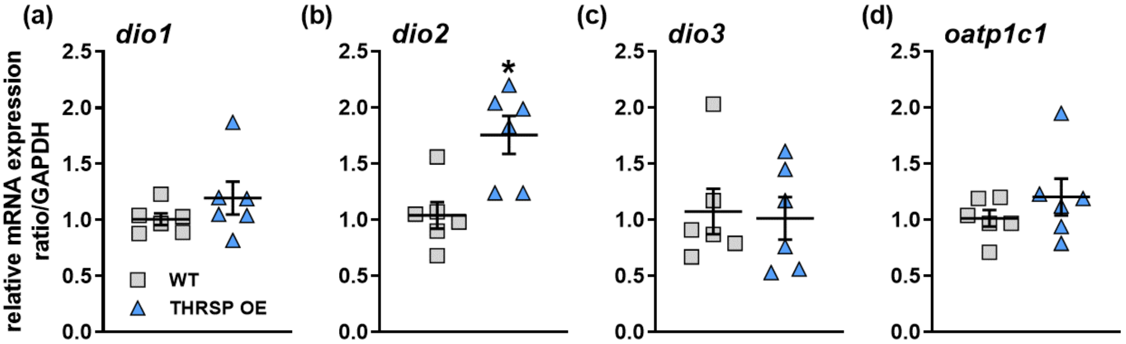

Supplementary Figure 4. Striatal expressions of iodothyronine deiodinases and other transporter for thyroid hormones in THRSP OE mice. The **(a)** deiodinase 1 [*dio1*], **(b)** deiodinase 2 [*dio2*], **(c)** deiodinase 3 [*dio2*], and **(d)** organic anion transporting polypeptide 1C1 [*oatp1c1*] mRNA levels in the mouse striatum (n = 6 mice/group; **(a)** two-tailed student t-test [t=1.70, df=5], P = 0.151; **(b)** two-tailed student t-test [t=2.73, df=5], P = 0.041; **(c)** two-tailed student t-test [t=0.180, df=5], P = 0.864; **(d)** two-tailed student t-test [t=0.808, df=5, P = 0.456]). No significant changes in the mRNA levels between THRSP OE mice and WT. Values are presented as the mean ± S.E.M.

Supplementary Figure 5

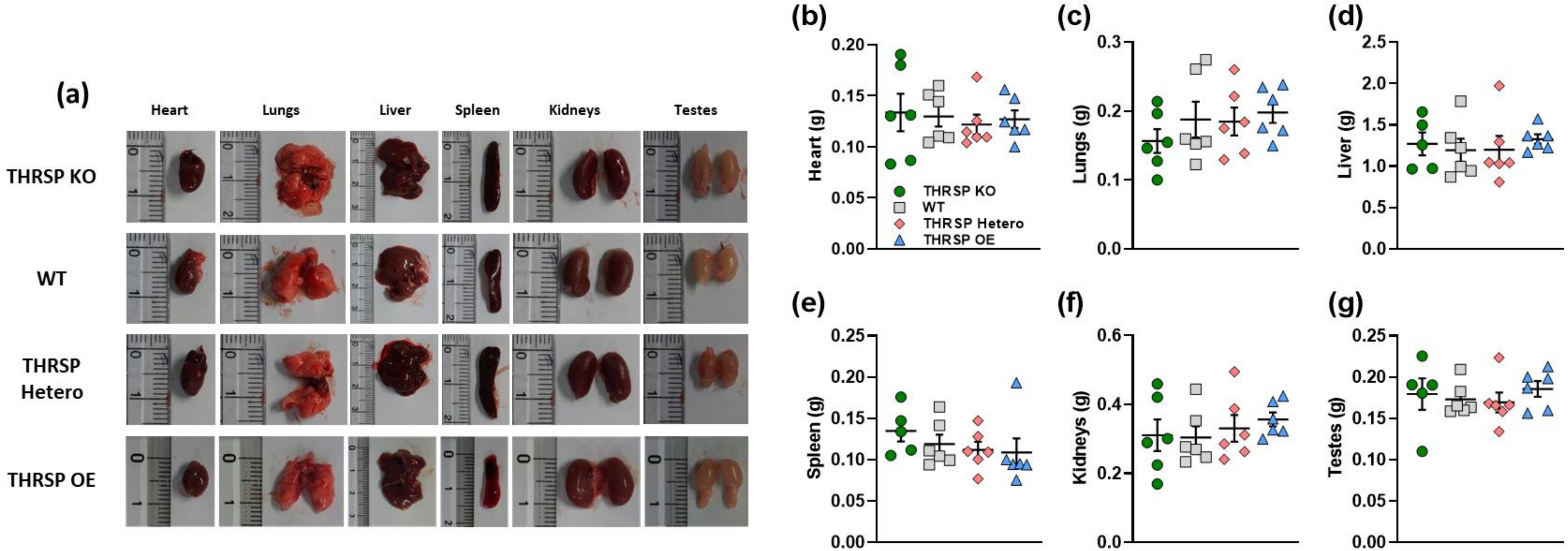

Supplementary Figure 5. Evaluation of other major organs in mice. (a) Other organ tissues were harvested including the (**b**) heart, (**c**) lungs, (**d**) liver, (**e**) spleen, (**f**) kidneys, and (**g**) testes (n = 6 mice/group; **(b)** one-way ANOVA, $F_{(3, 20)} = 0.160$, $P = 0.922$; **(c)** one-way ANOVA, $F_{(3, 20)} = 0.784$, $P = 0.517$; **(d)** one-way ANOVA, $F_{(3, 19)} = 0.244$, $P = 0.865$; **(e)** one-way ANOVA, $F_{(3, 19)} = 0.724$, $P = 0.550$; **(f)** one-way ANOVA, $F_{(3, 20)} = 0.441$, $P = 0.726$; **(g)** one-way ANOVA, $F_{(3, 19)} = 0.361$, $P = 0.782$). No gross abnormalities were observed between groups. Values are presented as the mean ± S.E.M.

Supplementary Figure 6

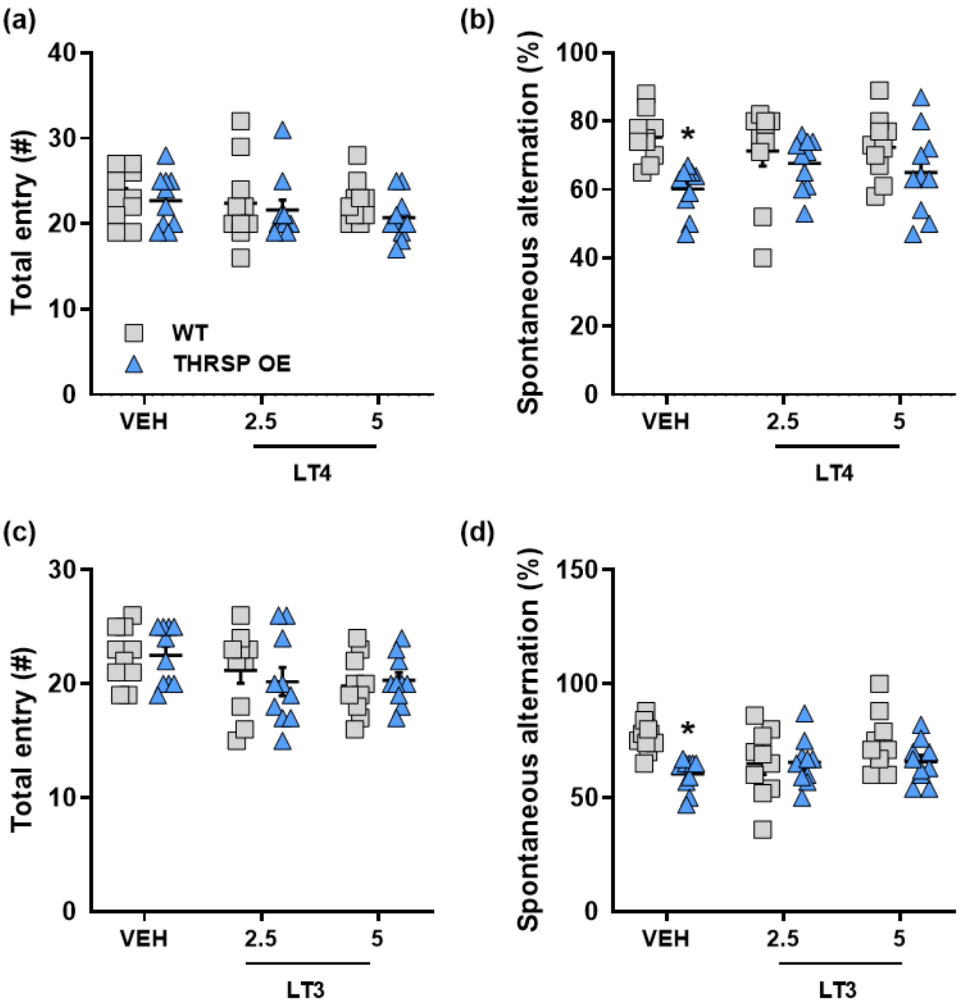

Supplementary Figure 6. Effects of low doses of LT3 and LT4 on inattention in THRSP OE mice. Separate cohorts of mice were treated with LT4 and LT3 (2.5, 5 mg kg^{-1}) for seven days. Thirty minutes after the last treatment, mice were exposed to the Y-maze test, and the (a, c) total arm entries and (b, d) spontaneous alternations during Y-maze test were scored (n = 10 mice/group; (a) two-way ANOVA, $F_{(1, 54)} = 1.48$, $P = 0.228$; (b) two-way ANOVA, $F_{(1, 54)} = 11.4$, $P = 0.001$; (c) two-way ANOVA, $F_{(1, 54)} = 0.0308$, $P = 0.861$; (d) two-way ANOVA, $F_{(1, 54)} = 8.62$, $P = 0.005$). Treatment with thyroid hormones at lower doses have no significant effects on inattention in THRSP OE mice. Values are presented as the mean ± S.E.M. *$P < 0.05$, by one-way ANOVA with Bonferroni's multiple comparison relative to WT-VEH.

Supplementary Figure 7

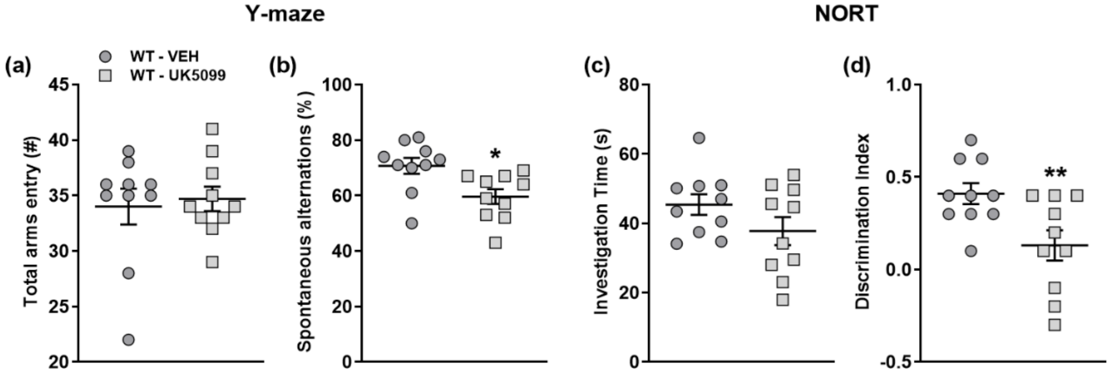

Supplementary Figure 7. Effects of MCT antagonist UK 5099 on attention in C57BL6/ (WT) mice. Separate cohorts of WT mice were treated with UK 5099 (5 mg kg−1) for seven days. Thirty minutes after the last treatment, the mice were subjected to the Y-maze test and NORT, and the (a) total arm entry, (b) spontaneous alternations, (c) investigation time, and (d) discrimination index were scored (n = 10 mice/group; (a) two-tailed student t-test [t=0.372, df=9], P = 0.718; (b) two-tailed student t-test [t=2.60, df=9], P = 0.029; (c) two-tailed student t-test [t=1.50, df=9], P = 0.167; (d) two-tailed student t-test [t=3.28, df=9], P = 0.010). Antagonism of MCT8 induces inattention in mice, indicating its role in attention. Values are presented as the mean ± S.E.M. *P < 0.05, **P < 0.01, by t-test relative to VEH-treated WT.

Untruncated western blots (Figure 1c and Supplementary figure 1a); (n=6)

B-actin (WT vs THRSP OE 1-3)

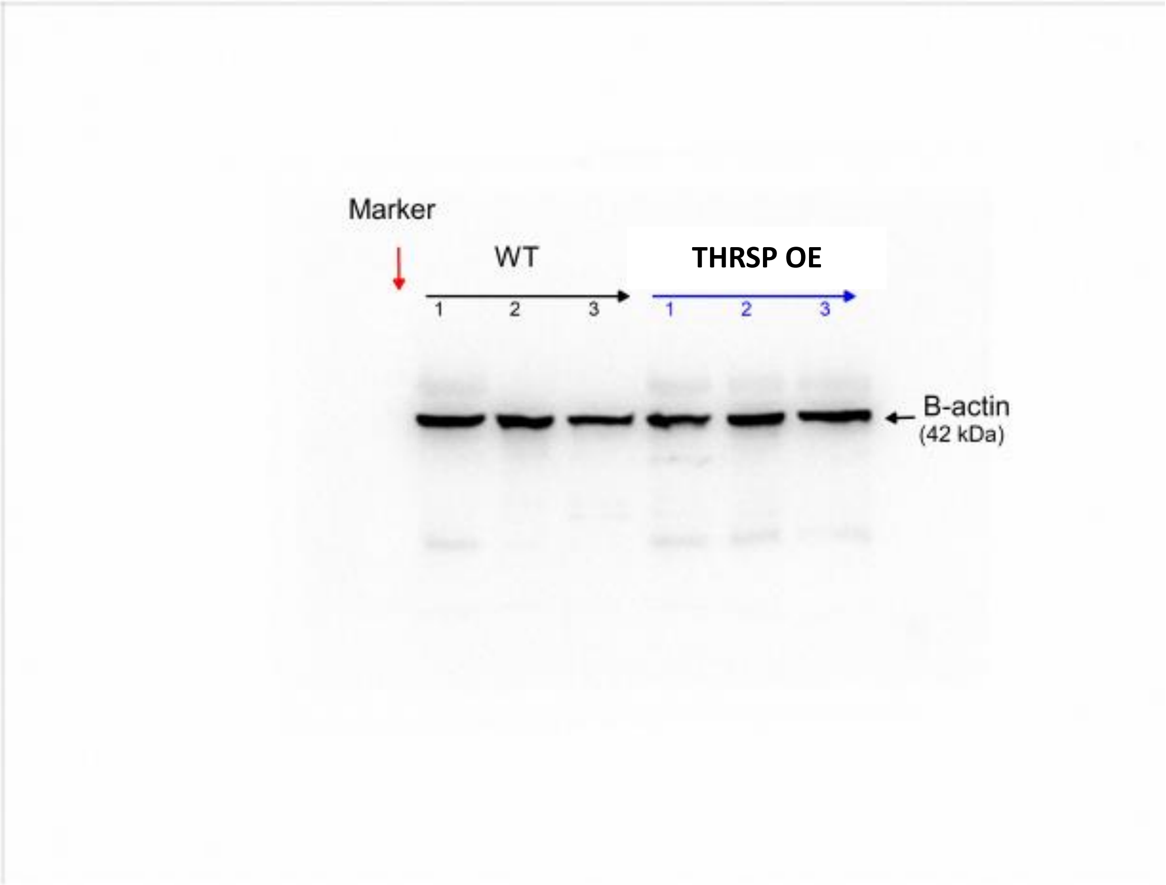

C:\Users\Admin\OneDrive\THRSP OE and KO mice\For PUBLICATION\FINAL\Western blot - calculation\WT vs THRSP OE vs HET vs KO\WT vs OE\B-actin (WT vs THRSP OE 1-3).scn

Acquisition Information

Imager	ChemiDoc MP
Exposure Time (sec)	2.644 (Auto - Intense Bands)
Flat Field	Applied (Lens)
Serial Number	731BR03542
Software Version	5.2.1
Application	Chemi
Excitation Source	No Illumination
Emission Filter	No Filter
Binning	3x3

Note: Blots were used to compare THRSP protein expression levels in THRSP OE / THRSP KO (figure 1c) and THRSP Hetero (Supplementary figure 1) versus WT.

B-actin (WT vs THRSP OE - 4-6)

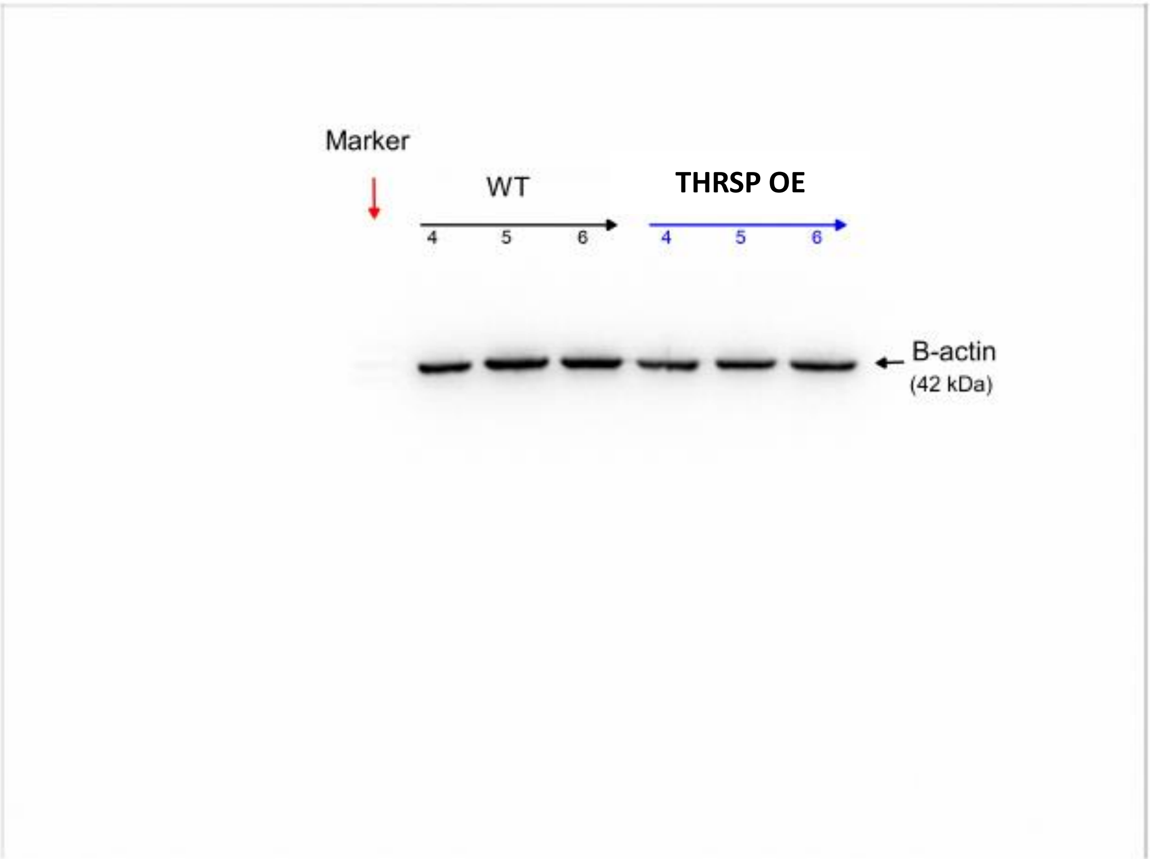

C:\Users\Admin\OneDrive\THRSP OE and KO mice\For PUBLICATION\FINAL\Western blot - calculation\WT vs THRSP OE vs HET vs KO\WT vs OE\B-actin (WT vs THRSP OE 4-6).scn

---

Acquisition Information

Imager	ChemiDoc MP
Exposure Time (sec)	1.093 (Auto - Intense Bands)
Flat Field	Applied (Lens)
Serial Number	731BR03542
Software Version	5.2.1
Application	Chemi
Excitation Source	No Illumination
Emission Filter	No Filter
Binning	3x3

B-actin (WT vs THRSP HET vs KO 1-3)

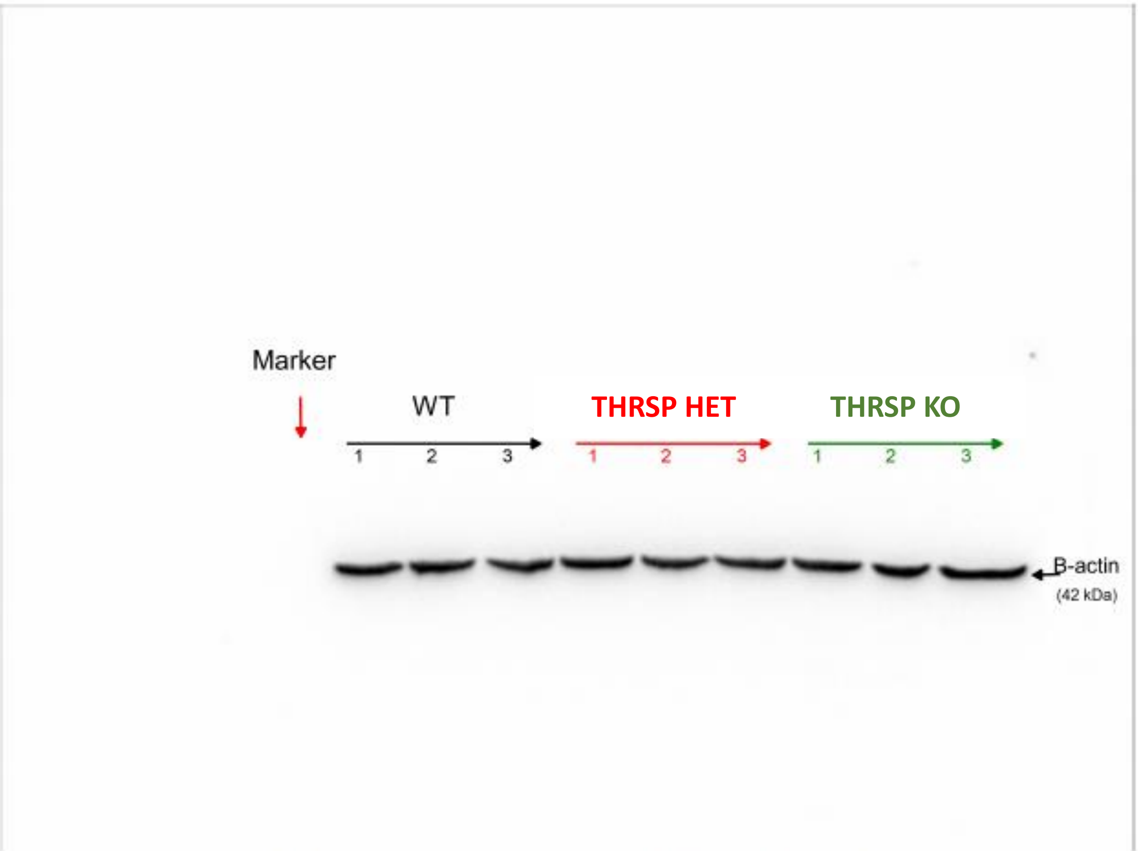

C:\Users\Admin\OneDrive\THRSP OE and KO mice\For PUBLICATION\FINAL\Western blot - calculation\WT vs THRSP OE vs HET vs KO\WT vs HET vs KO\B-actin (WT vs THRSP HET vs KO 1-3).scn

---

Acquisition Information

Imager	ChemiDoc MP
Exposure Time (sec)	1.646 (Auto - Intense Bands)
Flat Field	Applied (Lens)
Serial Number	731BR03542
Software Version	5.2.1
Application	Chemi
Excitation Source	No Illumination
Emission Filter	No Filter
Binning	3x3

B-actin (WT vs THRSP HET vs KO - 4-6)

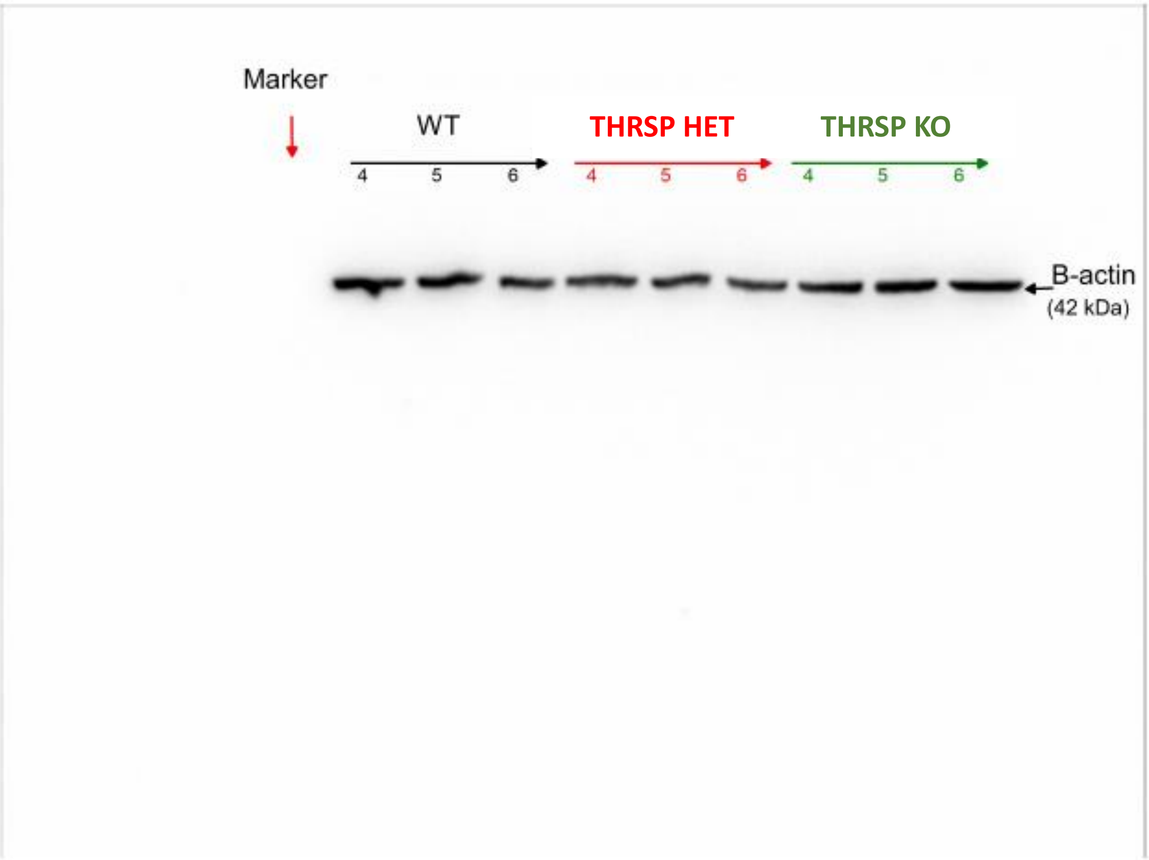

C:\Users\Admin\OneDrive\THRSP OE and KO mice\For PUBLICATION\FINAL\Western blot - calculation\WT vs THRSP OE vs HET vs KO\WT vs HET vs KO\B-actin (WT vs THRSP HET vs KO 4-6).scn

---

Acquisition Information

Imager	ChemiDoc MP
Exposure Time (sec)	1.209 (Auto - Intense Bands)
Flat Field	Applied (Lens)
Serial Number	731BR03542
Software Version	5.2.1
Application	Chemi
Excitation Source	No Illumination
Emission Filter	No Filter
Binning	3x3

Thrsp (WT vs THRSP OE 1-3)

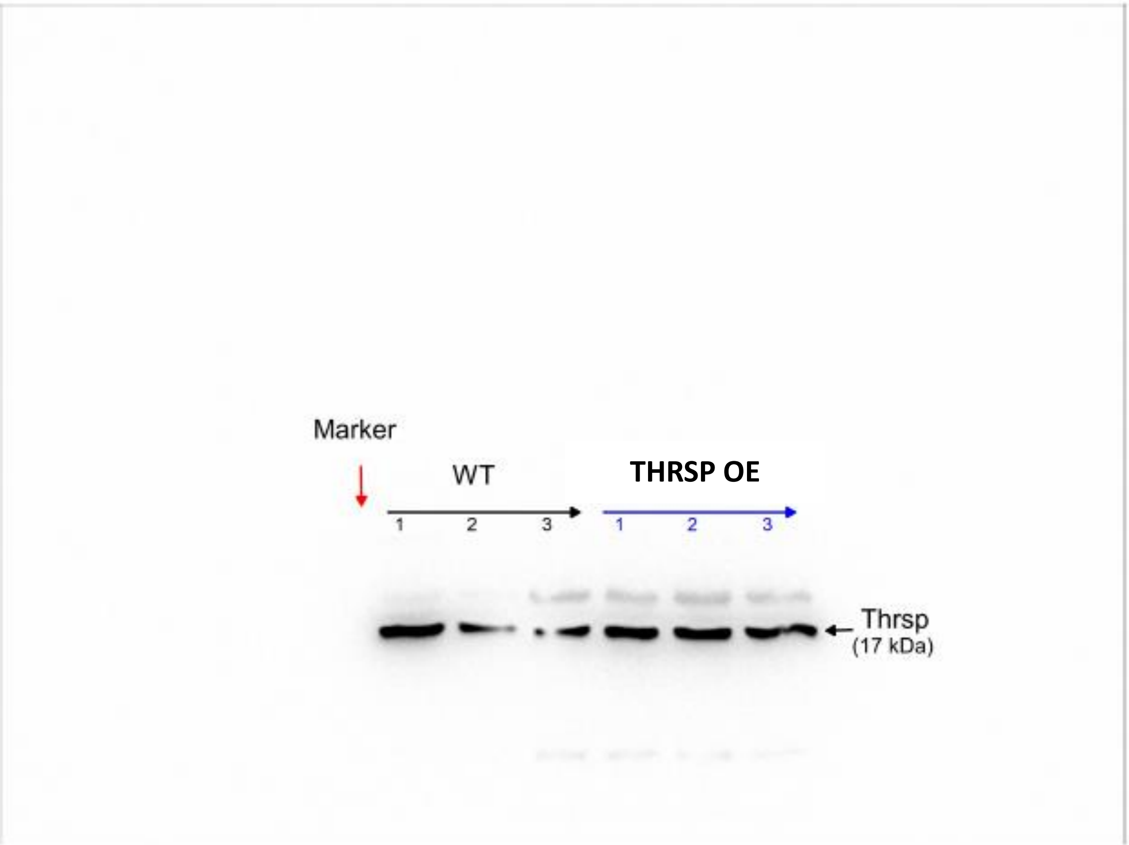

C:\Users\Admin\OneDrive\THRSP OE and KO mice\For PUBLICATION\FINAL\Western blot - calculation\WT vs THRSP OE vs HET vs KO\WT vs OE\Thrsp (WT vs THRSP OE 1-3).scn

---

Acquisition Information

Imager	ChemiDoc MP
Exposure Time (sec)	3.420 (Auto - Intense Bands)
Flat Field	Applied (Lens)
Serial Number	731BR03542
Software Version	5.2.1
Application	Chemi
Excitation Source	No Illumination
Emission Filter	No Filter
Binning	3x3

THRSP (WT vs THRSP OE - 4-6)

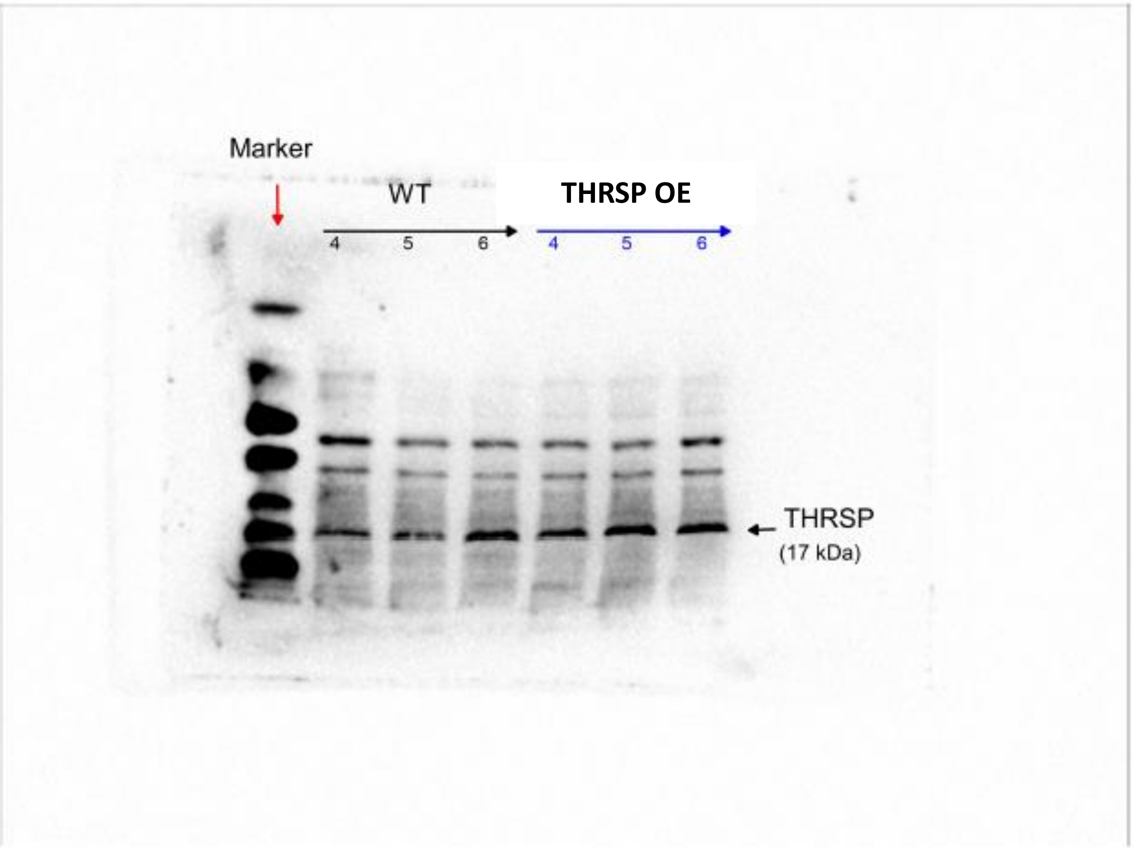

C:\Users\Admin\OneDrive\THRSP OE and KO mice\For PUBLICATION\FINAL\Western blot - calculation\WT vs THRSP OE vs HET vs KO\WT vs OE\Thrsp (WT vs THRSP OE 4-6).scn

---

Acquisition Information

Imager	ChemiDoc MP
Exposure Time (sec)	0.750 (Auto - Intense Bands)
Flat Field	Applied (Lens)
Serial Number	731BR03542
Software Version	5.2.1
Application	Chemi
Excitation Source	No Illumination
Emission Filter	No Filter
Binning	3x3

Thrsp (WT vs THRSP HET vs KO 1-3)

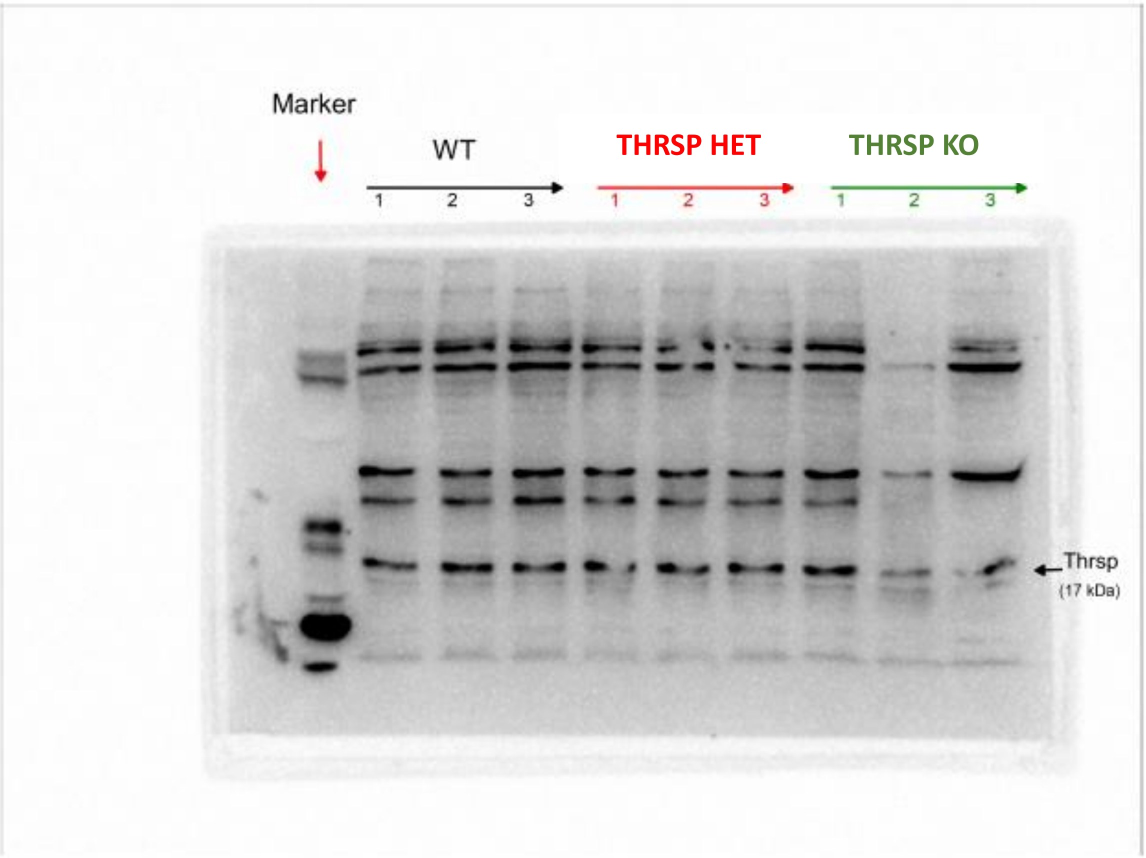

C:\Users\Admin\OneDrive\THRSP OE and KO mice\For PUBLICATION\FINAL\Western blot - calculation\WT vs THRSP OE vs HET vs KO\WT vs HET vs KO\Thrsp (WT vs THRSP HET vs KO 1-3).scn

---

Acquisition Information

Imager	ChemiDoc MP
Exposure Time (sec)	3.790 (Auto - Intense Bands)
Flat Field	Applied (Lens)
Serial Number	731BR03542
Software Version	5.2.1
Application	Chemi
Excitation Source	No Illumination
Emission Filter	No Filter
Binning	3x3

THRSP (WT vs THRSP HET vs KO - 4-6)

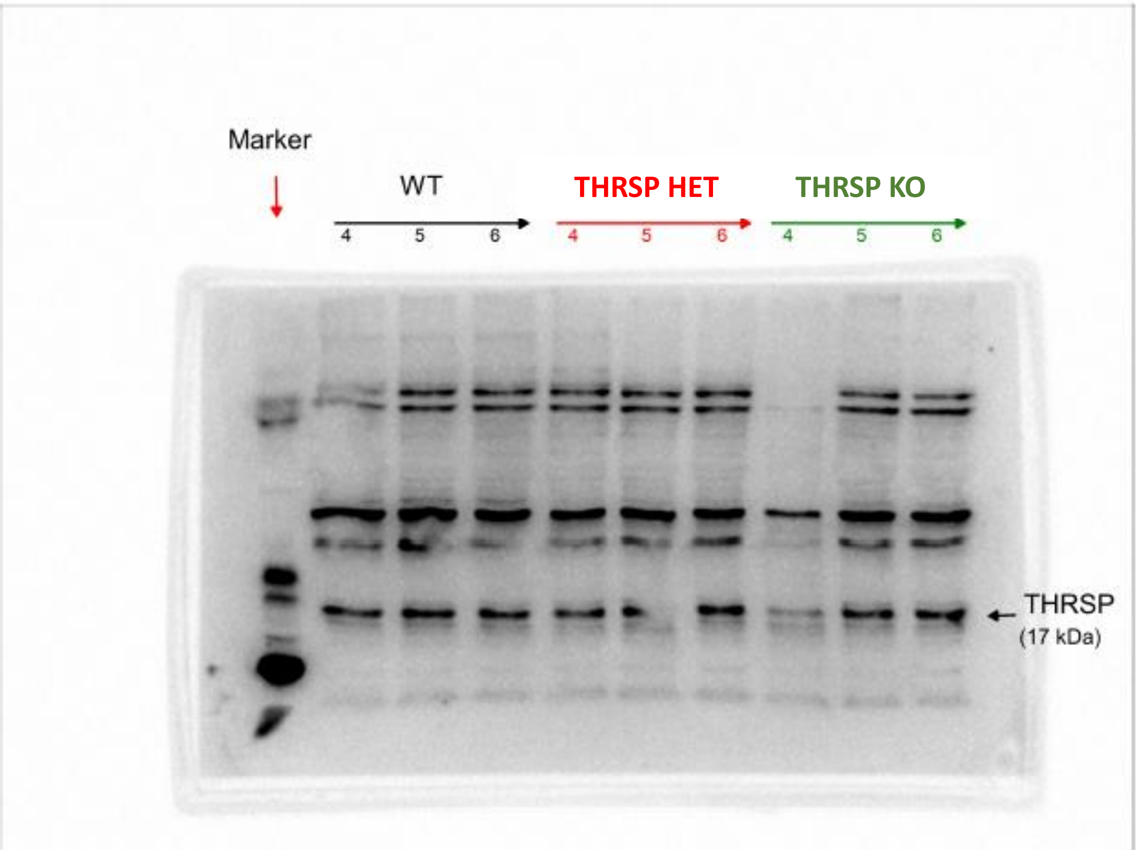

C:\Users\Admin\OneDrive\THRSP OE and KO mice\For PUBLICATION\FINAL\Western blot - calculation\WT vs THRSP OE vs HET vs KO\WT vs HET vs KO\Thrsp (WT vs THRSP HET vs KO 4-6).scn

---

Acquisition Information

Imager	ChemiDoc MP
Exposure Time (sec)	3.811 (Auto - Intense Bands)
Flat Field	Applied (Lens)
Serial Number	731BR03542
Software Version	5.2.1
Application	Chemi
Excitation Source	No Illumination
Emission Filter	No Filter
Binning	3x3

Untruncated western blots (Figure 10a); (n=6)

B-actin (WT vs THRSP OE - VEH 1-3)

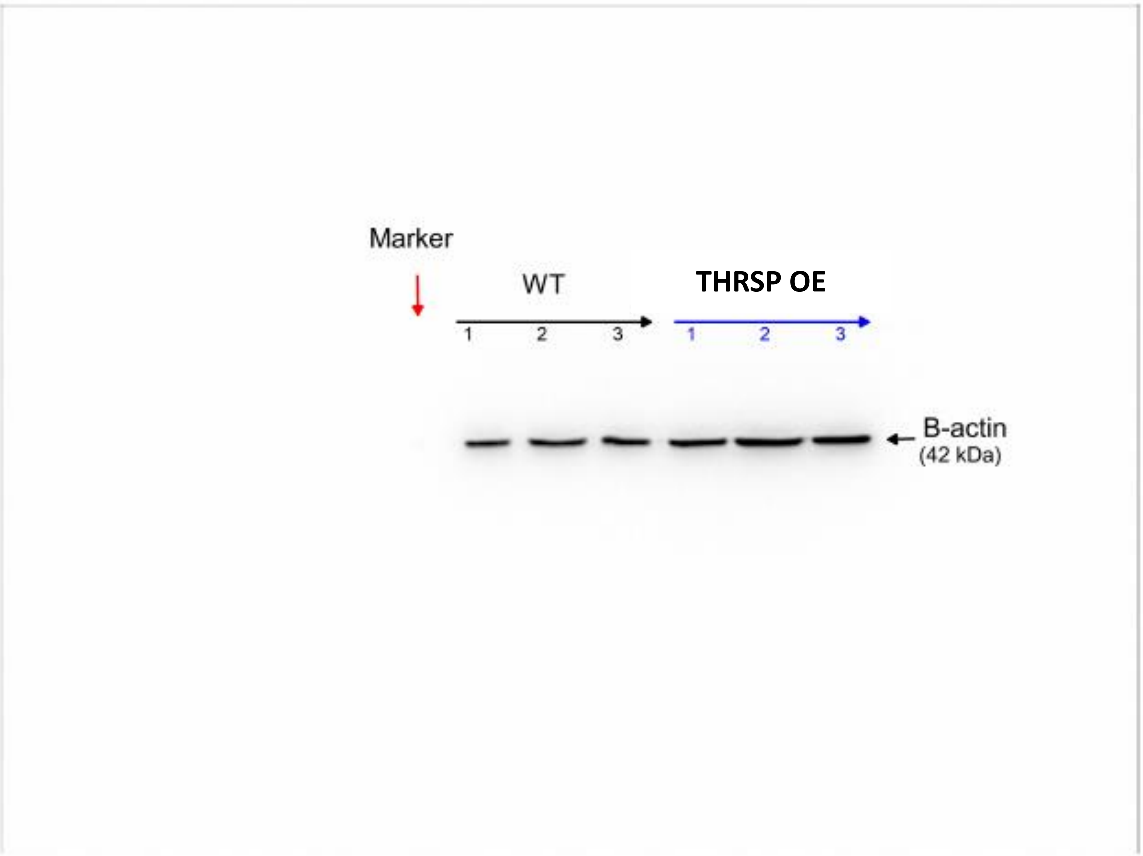

C:\Users\Admin\OneDrive\THRSP OE and KO mice\For PUBLICATION\Western blot\FINAL - western blot\VEH\B-actin (WT vs THRSP OE - VEH 1-3).scn

Acquisition Information

Imager	ChemiDoc MP
Exposure Time (sec)	2.874 (Auto - Intense Bands)
Flat Field	Applied (Lens)
Serial Number	731BR03542
Software Version	5.2.1
Application	Chemi
Excitation Source	No Illumination
Emission Filter	No Filter
Binning	3x3

B-actin (WT vs THRSP OE - VEH - 4-6)

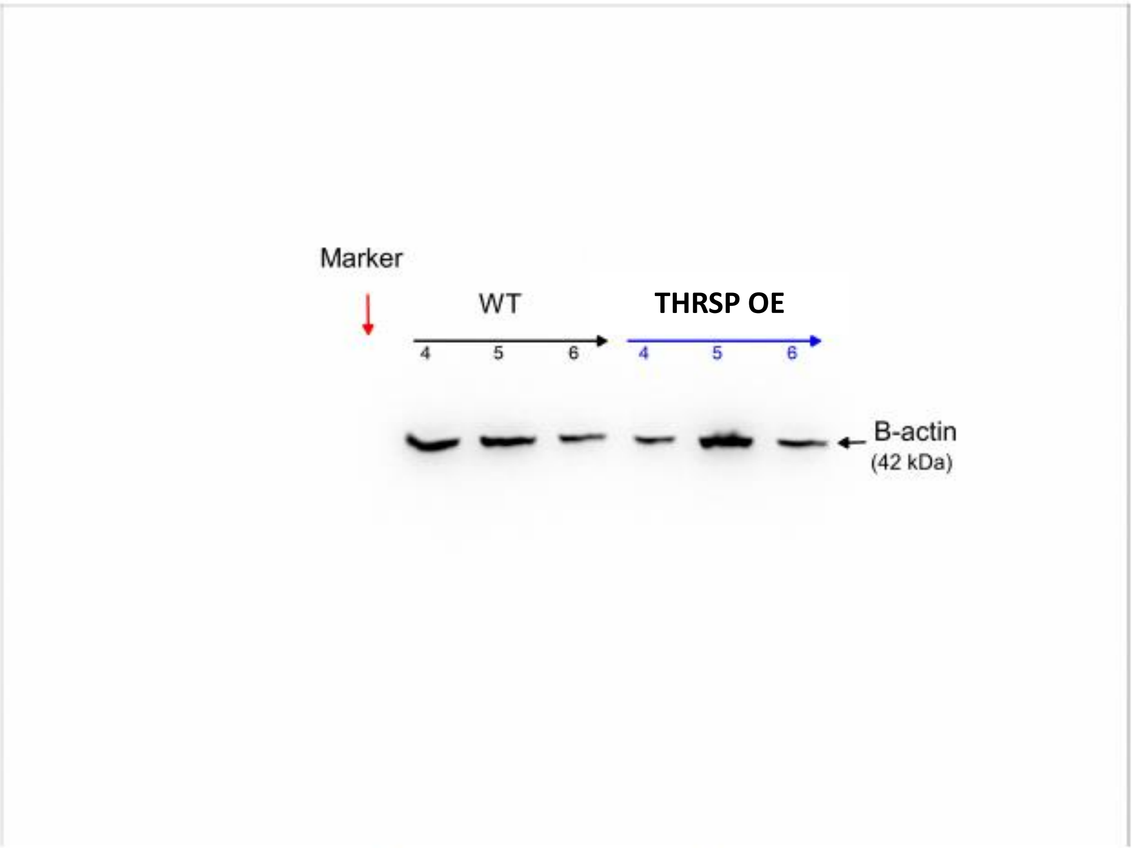

C:\Users\Admin\OneDrive\THRSP OE and KO mice\For PUBLICATION\Western blot\FINAL - western blot\VEH\B-actin (WT vs THRSP OE - VEH 4-6).scn

---

Acquisition Information

Imager	ChemiDoc MP
Exposure Time (sec)	5.990 (Auto - Intense Bands)
Flat Field	Applied (Lens)
Serial Number	731BR03542
Software Version	5.2.1
Application	Chemi
Excitation Source	No Illumination
Emission Filter	No Filter
Binning	3x3

MCT8 (WT vs THRSP OE - VEH 1-3)

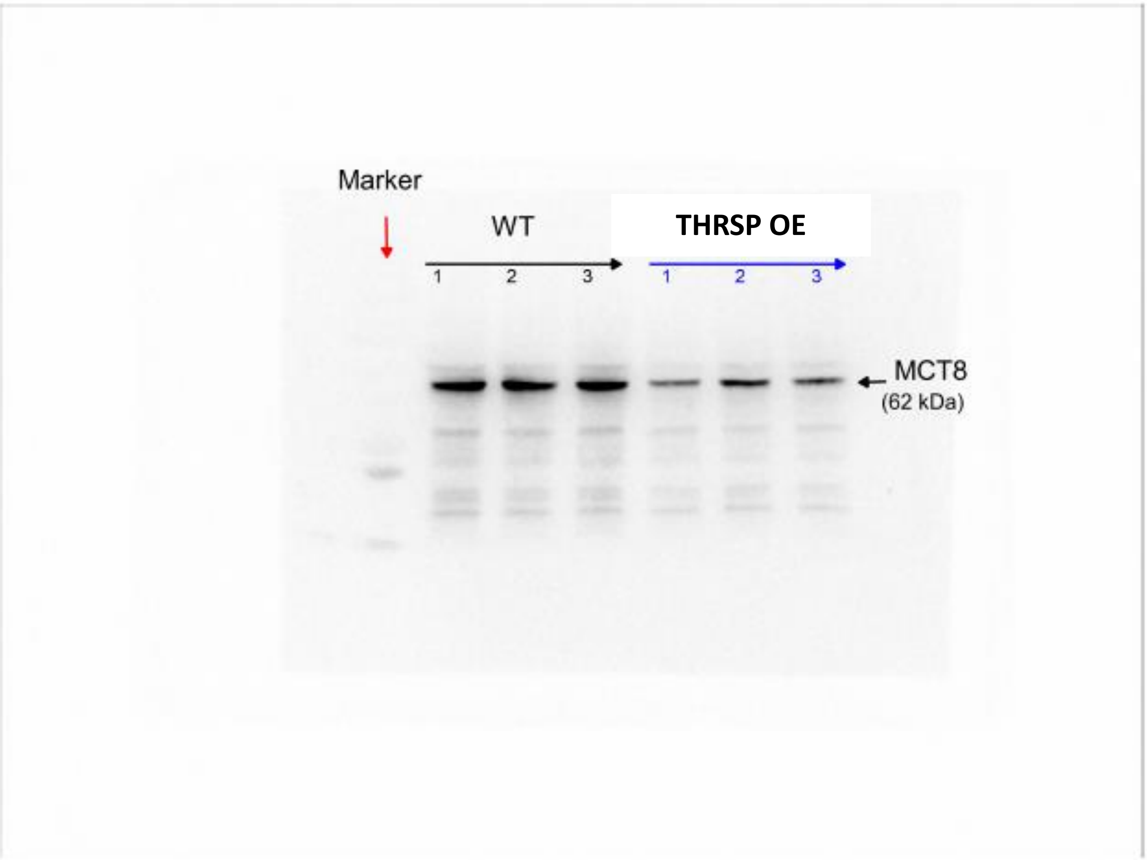

C:\Users\Admin\OneDrive\THRSP OE and KO mice\For PUBLICATION\Western blot\FINAL - western blot\VEH\MCT8 (WT vs THRSP OE - VEH 1-3).scn

---

Acquisition Information

Imager	ChemiDoc MP
Exposure Time (sec)	4.828 (Auto - Intense Bands)
Flat Field	Applied (Lens)
Serial Number	731BR03542
Software Version	5.2.1
Application	Chemi
Excitation Source	No Illumination
Emission Filter	No Filter
Binning	3x3

MCT8 (WT vs THRSP OE - VEH - 4-6)

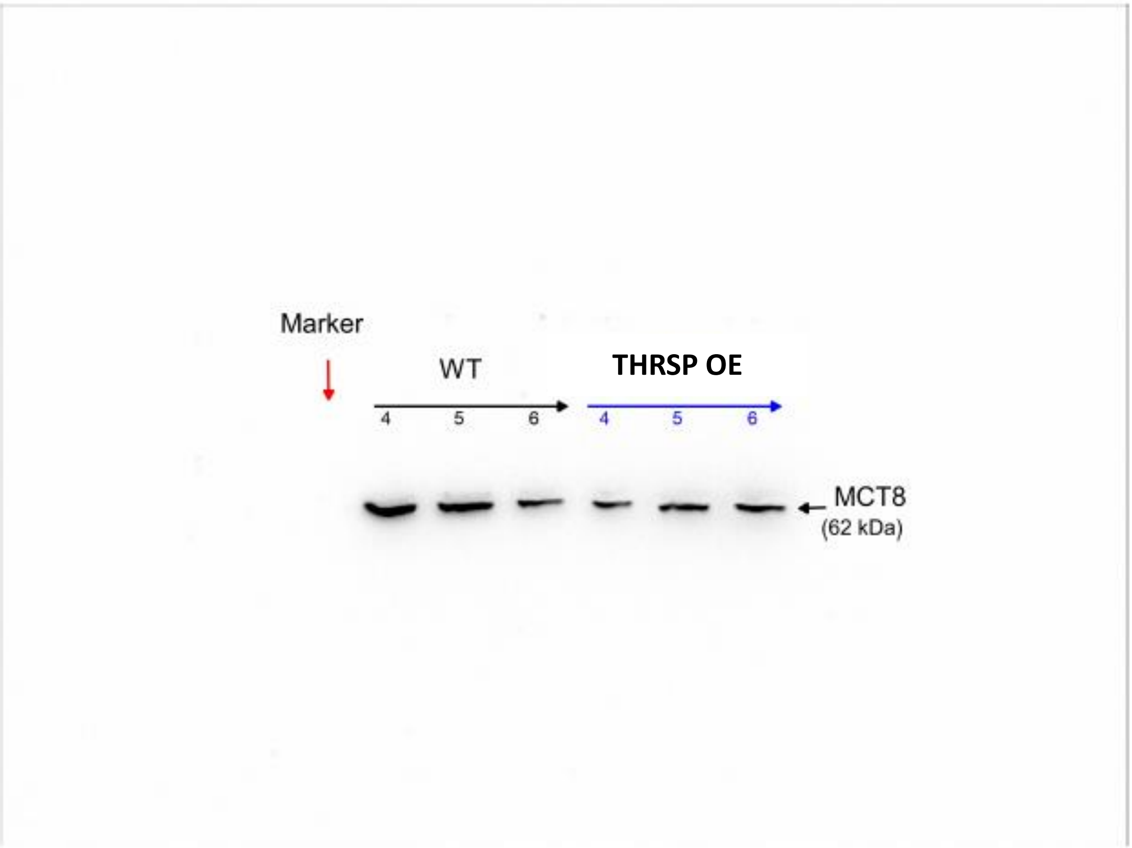

C:\Users\Admin\OneDrive\THRSP OE and KO mice\For PUBLICATION\Western blot\FINAL - western blot\VEH\MCT8 (WT vs THRSP OE - VEH 4-6).scn

---

Acquisition Information

Imager	ChemiDoc MP
Exposure Time (sec)	5.166 (Auto - Intense Bands)
Flat Field	Applied (Lens)
Serial Number	731BR03542
Software Version	5.2.1
Application	Chemi
Excitation Source	No Illumination
Emission Filter	No Filter
Binning	3x3

TRa (WT vs THRSP OE - VEH - 1-3)

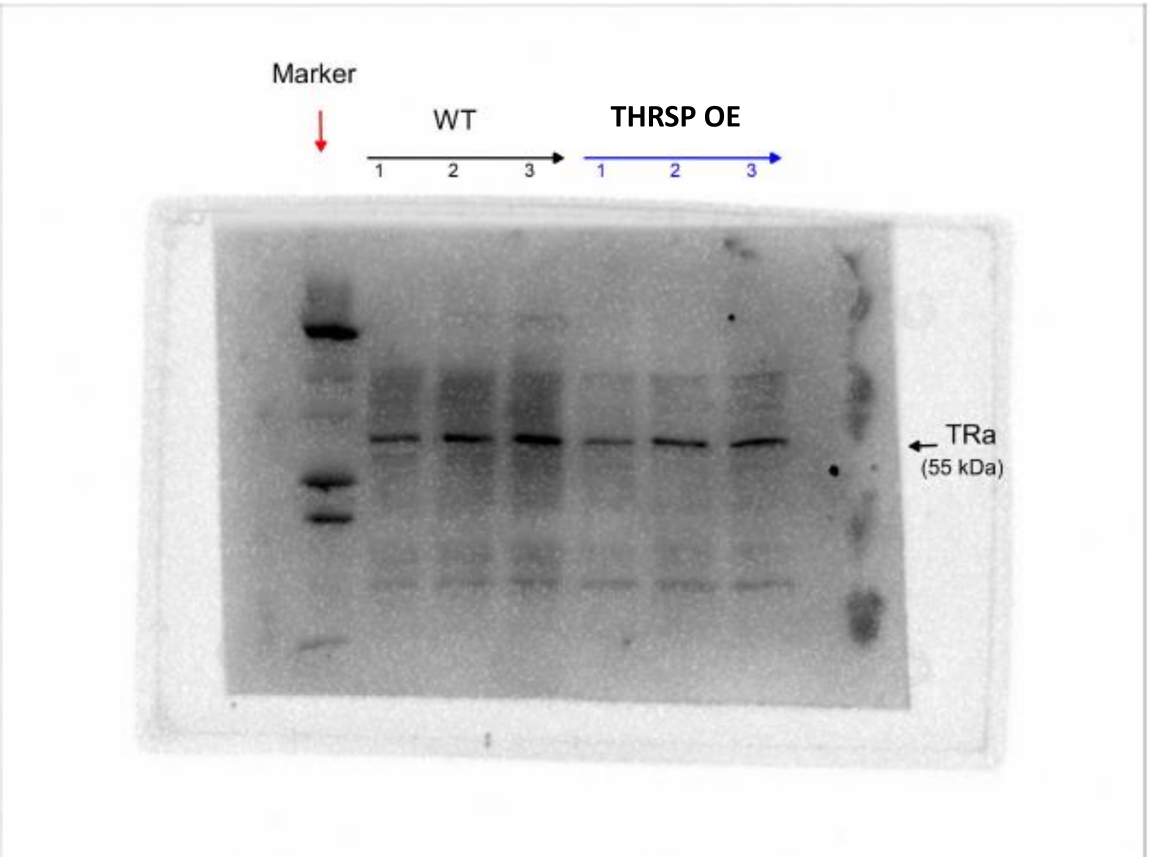

C:\Users\Admin\OneDrive\THRSP OE and KO mice\For PUBLICATION\Western blot\FINAL - western blot\VEH\TRa (WT vs THRSP OE - VEH 1-3).scn

---

Acquisition Information

Imager	ChemiDoc MP
Exposure Time (sec)	3.836 (Auto - Intense Bands)
Flat Field	Applied (Lens)
Serial Number	731BR03542
Software Version	5.2.1
Application	Chemi
Excitation Source	No Illumination
Emission Filter	No Filter
Binning	3x3

TRa (WT vs THRSP OE - VEH - 4-6)

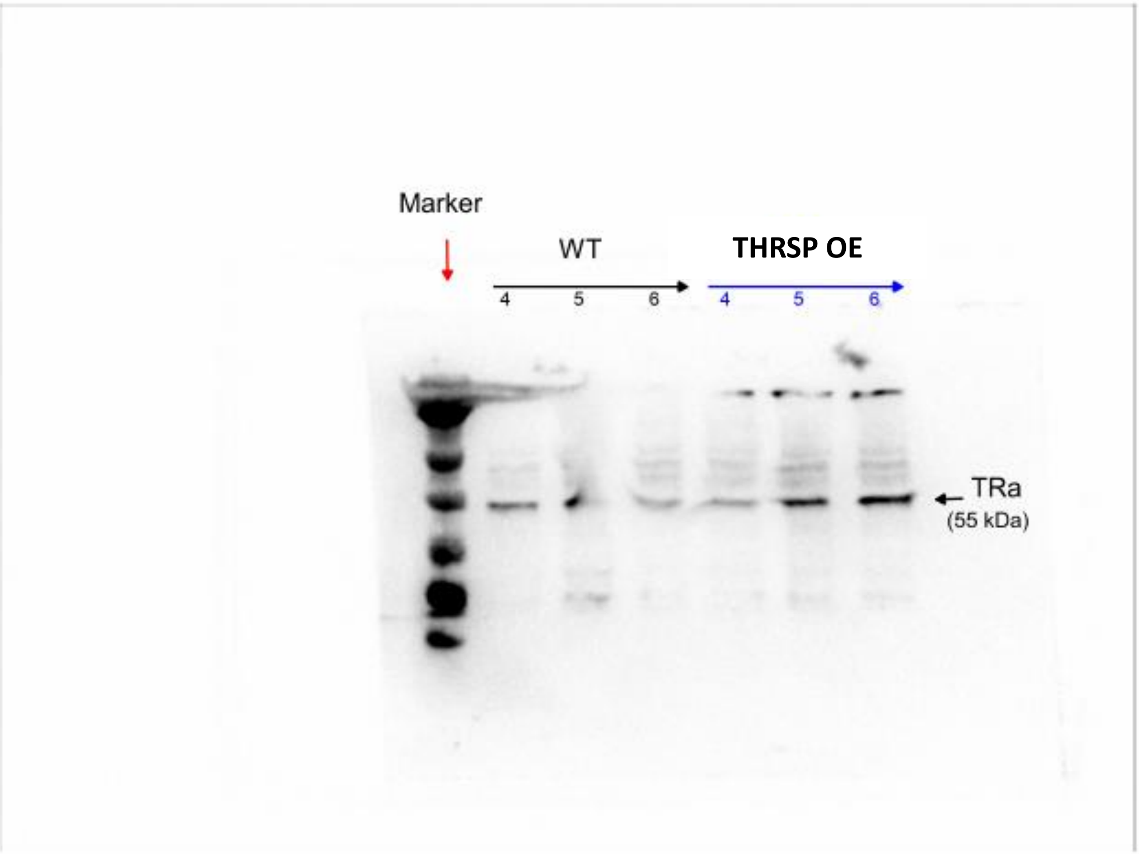

C:\Users\Admin\OneDrive\THRSP OE and KO mice\For PUBLICATION\Western blot\FINAL - western blot\VEH\TRa (WT vs THRSP OE - VEH 4-6).scn

---

Acquisition Information

Imager	ChemiDoc MP
Exposure Time (sec)	2.737 (Auto - Intense Bands)
Flat Field	Applied (Lens)
Serial Number	731BR03542
Software Version	5.2.1
Application	Chemi
Excitation Source	No Illumination
Emission Filter	No Filter
Binning	3x3

TRb (WT vs THRSP OE - VEH - 1-3)

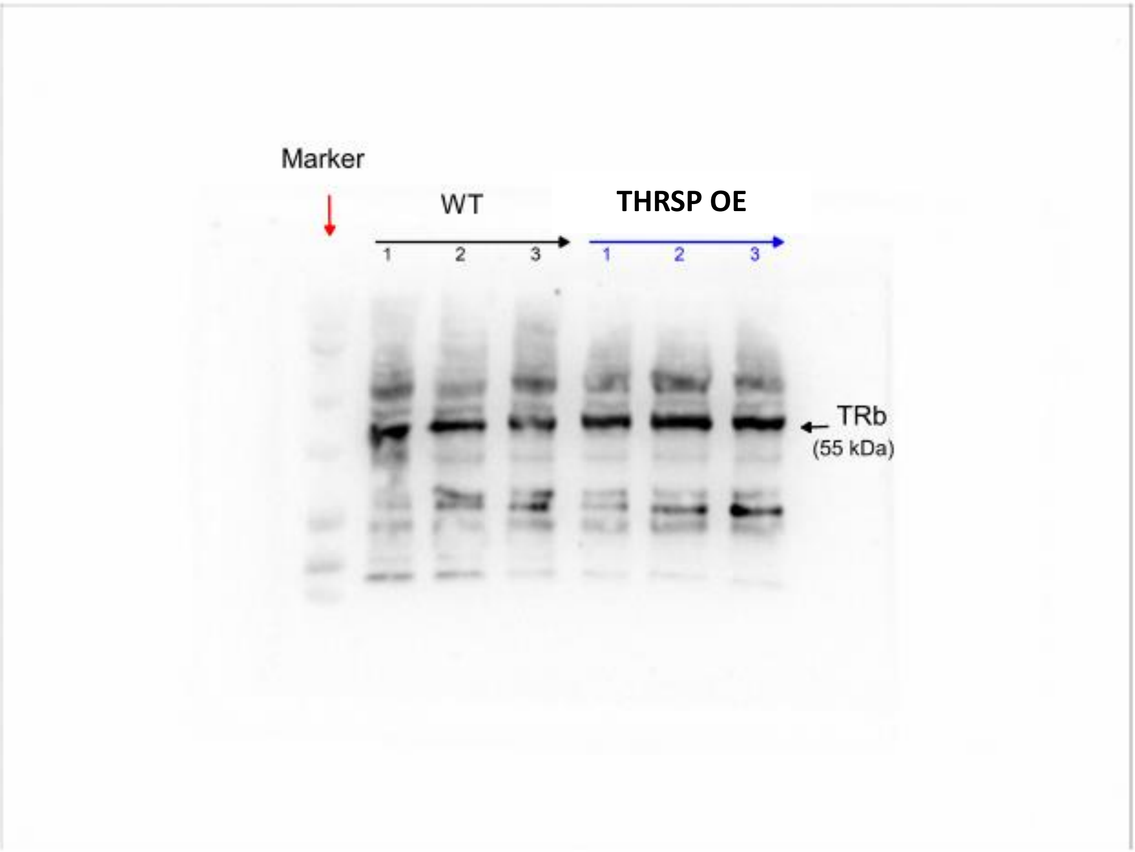

C:\Users\Admin\OneDrive\THRSP OE and KO mice\For PUBLICATION\Western blot\FINAL - western blot\VEH\TRb (WT vs THRSP OE - VEH 1-3).scn

---

Acquisition Information

Imager	ChemiDoc MP
Exposure Time (sec)	20.554 (Auto - Intense Bands)
Flat Field	Applied (Lens)
Serial Number	731BR03542
Software Version	5.2.1
Application	Chemi
Excitation Source	No Illumination
Emission Filter	No Filter
Binning	3x3

TRb (WT vs THRSP OE - VEH - 4-6)

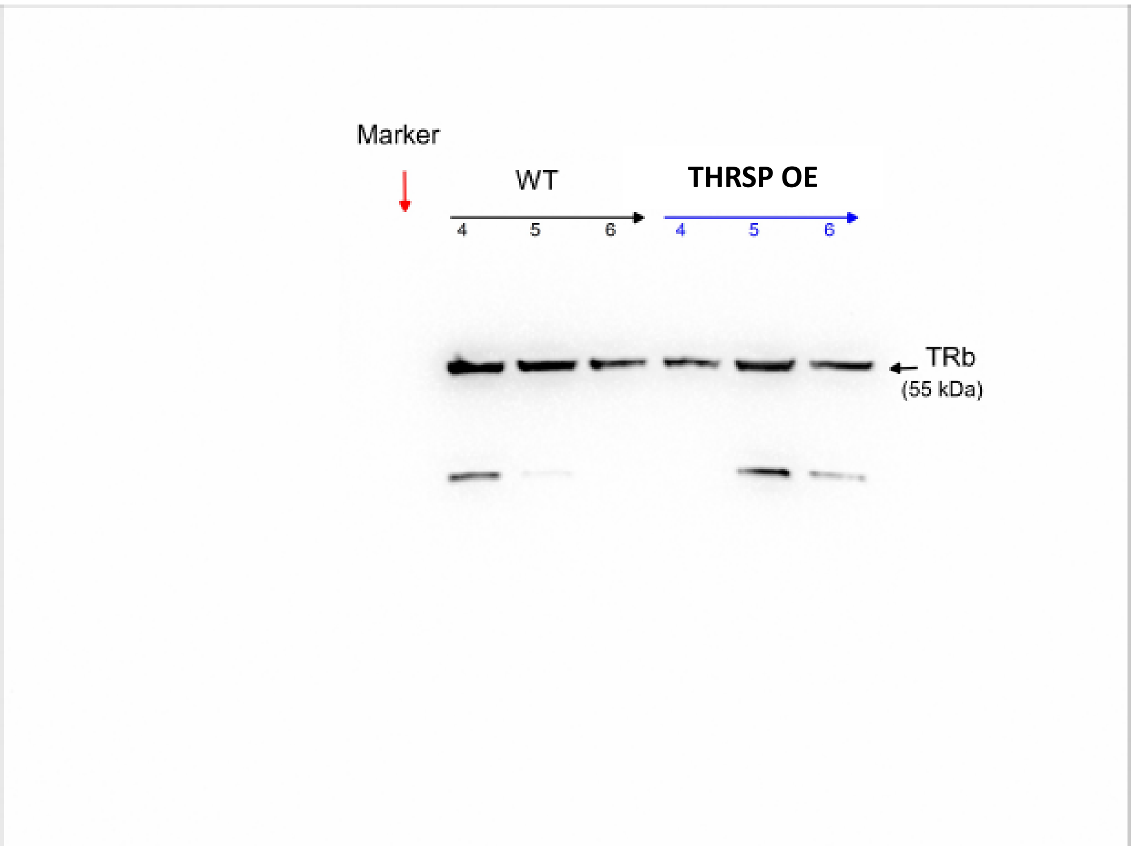

C:\Users\Admin\OneDrive\THRSP OE and KO mice\For PUBLICATION\Western blot\FINAL - western blot\VEH\TRb (WT vs THRSP OE - VEH 4-6).scn

---

Acquisition Information

Imager	ChemiDoc MP
Exposure Time (sec)	10.950 (Auto - Intense Bands)
Flat Field	Applied (Lens)
Serial Number	731BR03542
Software Version	5.2.1
Application	Chemi
Excitation Source	No Illumination
Emission Filter	No Filter
Binning	3x3

B-actin (WT vs THRSP OE T3 - 1-3)

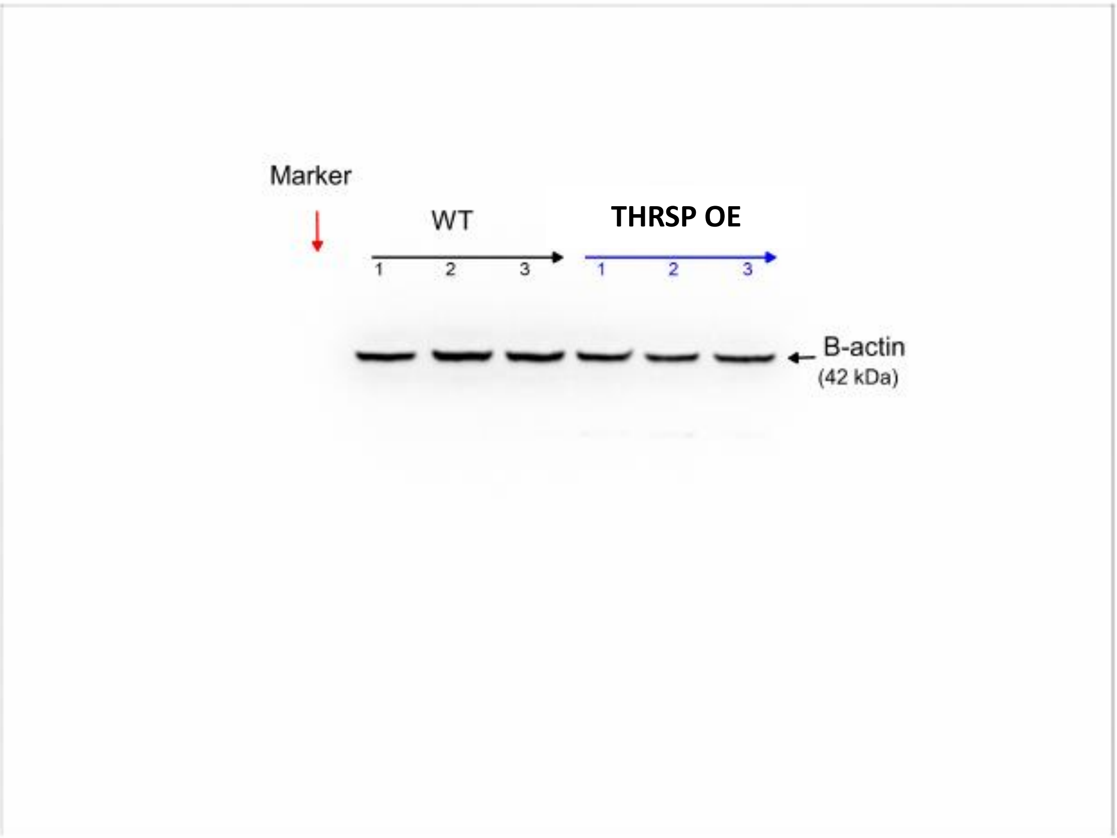

C:\Users\Admin\OneDrive\THRSP OE and KO mice\For PUBLICATION\Western blot\FINAL - western blot\T3\B-actin (WT vs THRSP OE T3 - 1-3).scn

Acquisition Information

Imager	ChemiDoc MP
Exposure Time (sec)	2.194 (Auto - Intense Bands)
Flat Field	Applied (Lens)
Serial Number	731BR03542
Software Version	5.2.1
Application	Chemi
Excitation Source	No Illumination
Emission Filter	No Filter
Binning	3x3

B-actin (WT vs THRSP OE T3 - 4-6)

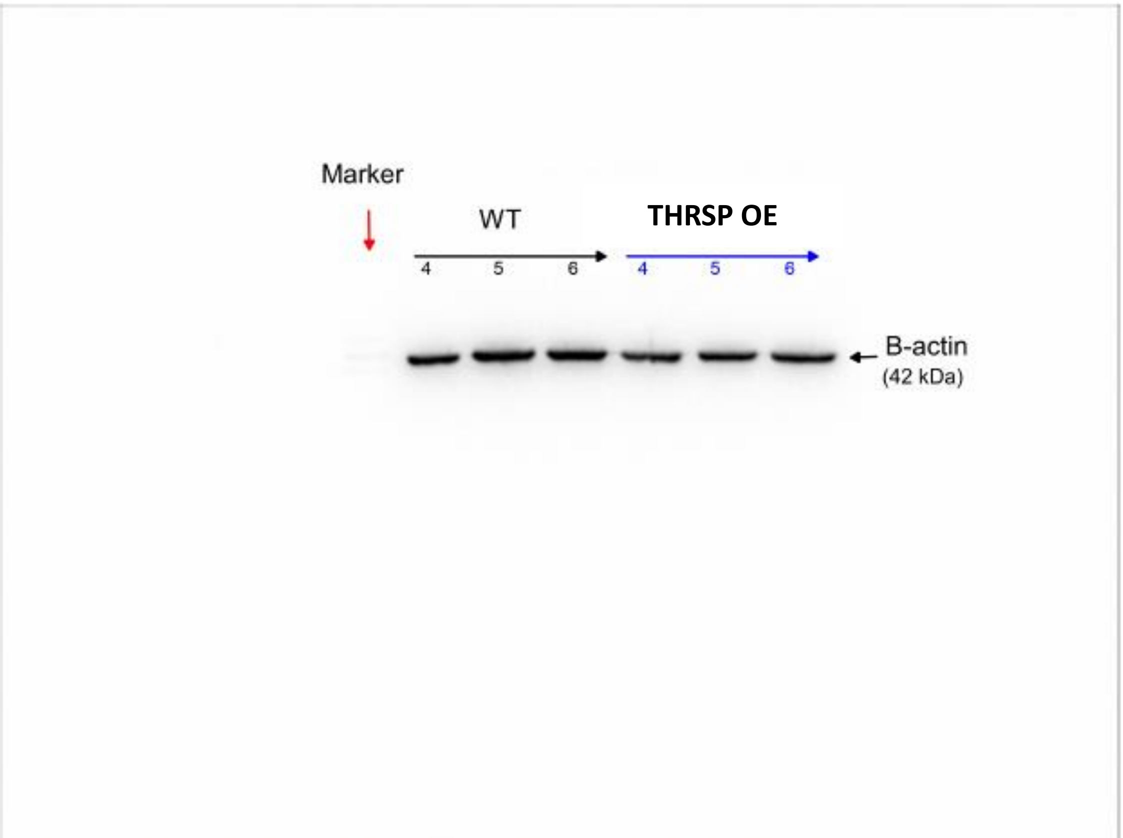

C:\Users\Admin\OneDrive\THRSP OE and KO mice\For PUBLICATION\Western blot\FINAL - western blot\T3\B-actin (WT vs THRSP OE T3 - 4-6).scn

Acquisition Information

Imager	ChemiDoc MP
Exposure Time (sec)	1.093 (Auto - Intense Bands)
Flat Field	Applied (Lens)
Serial Number	731BR03542
Software Version	5.2.1
Application	Chemi
Excitation Source	No Illumination
Emission Filter	No Filter
Binning	3x3

MCT8 (WT vs THRSP OE - T3 - 1-3)

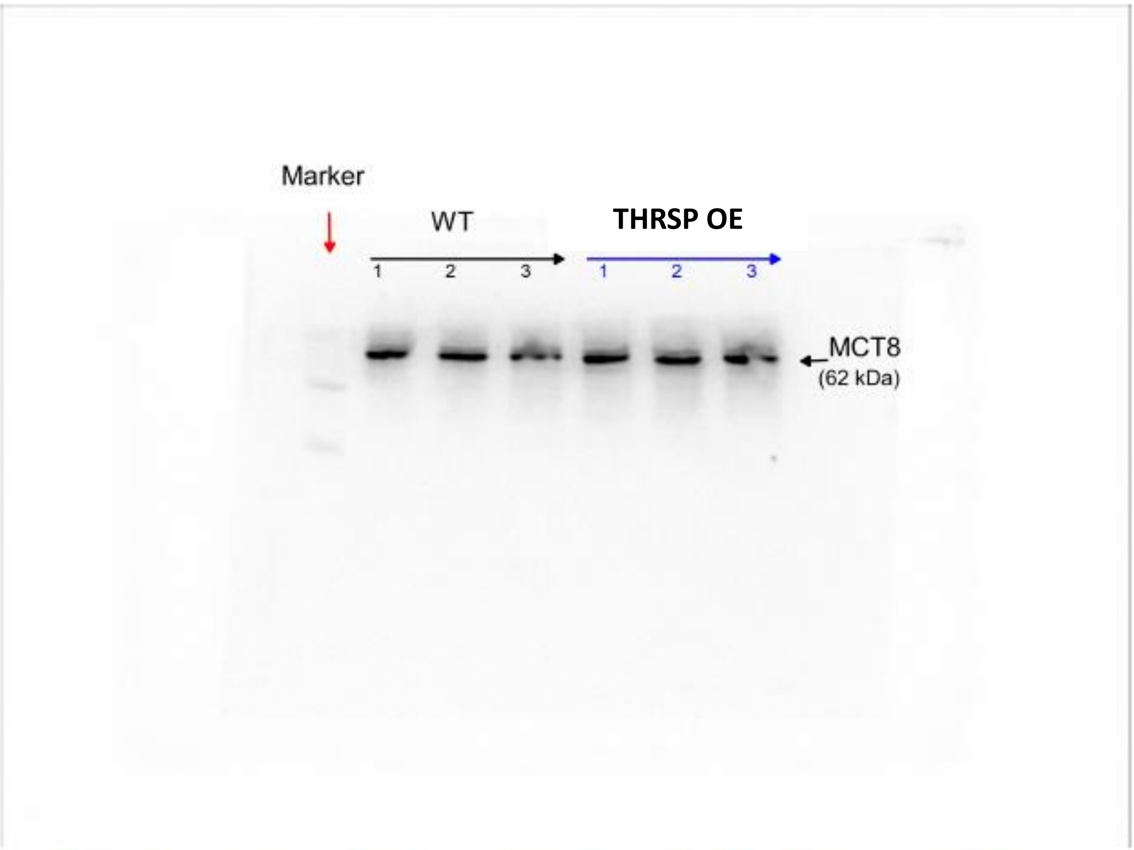

C:\Users\Admin\OneDrive\THRSP OE and KO mice\For PUBLICATION\Western blot\FINAL - western blot\T3\MCT8 (WT vs THRSP OE - T3 - 1-3).scn

---

Acquisition Information

Imager	ChemiDoc MP
Exposure Time (sec)	3.693 (Auto - Intense Bands)
Flat Field	Applied (Lens)
Serial Number	731BR03542
Software Version	5.2.1
Application	Chemi
Excitation Source	No Illumination
Emission Filter	No Filter
Binning	3x3

MCT8 (WT vs THRSP OE - T3 - 4-6)

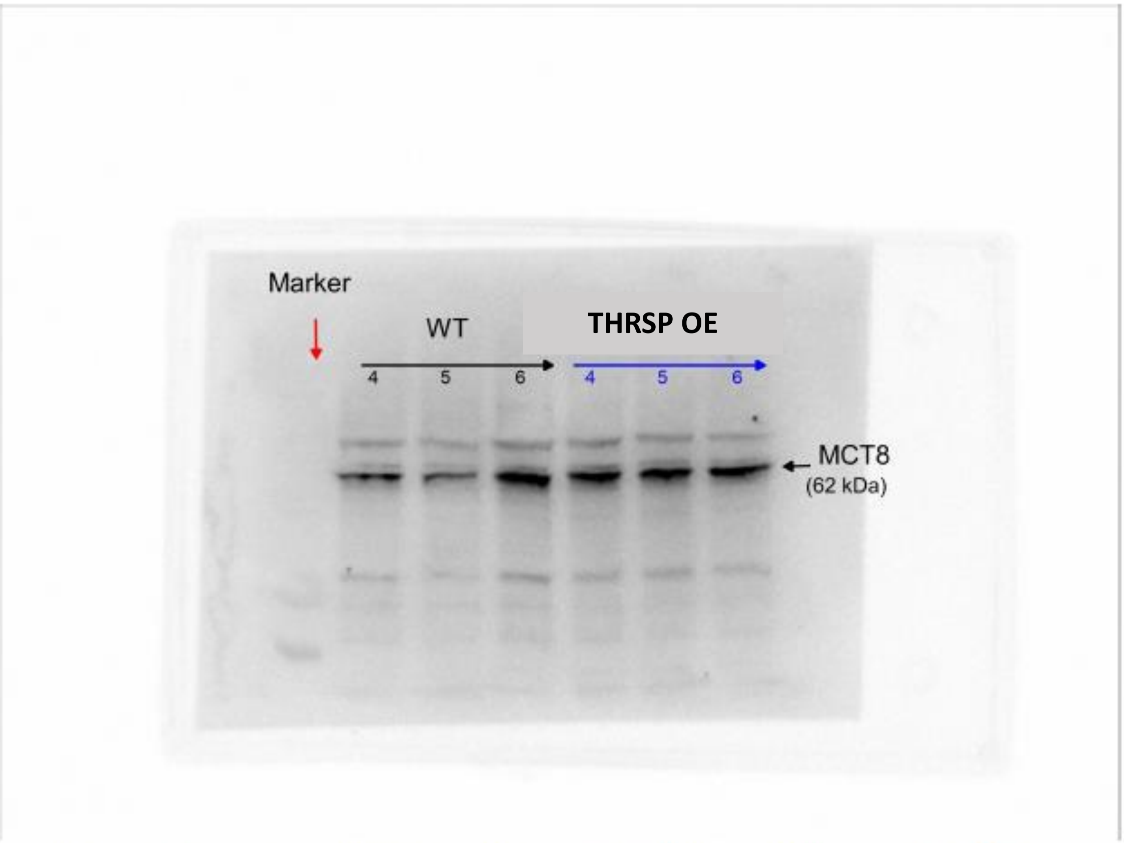

C:\Users\Admin\OneDrive\THRSP OE and KO mice\For PUBLICATION\Western blot\FINAL - western blot\T3\MCT8 (WT vs THRSP OE - T3 - 4-6).scn

Acquisition Information

Imager	ChemiDoc MP
Exposure Time (sec)	9.453 (Auto - Intense Bands)
Flat Field	Applied (Lens)
Serial Number	731BR03542
Software Version	5.2.1
Application	Chemi
Excitation Source	No Illumination
Emission Filter	No Filter
Binning	3x3

TRa (WT vs THRSP OE - T3 - 1-3)

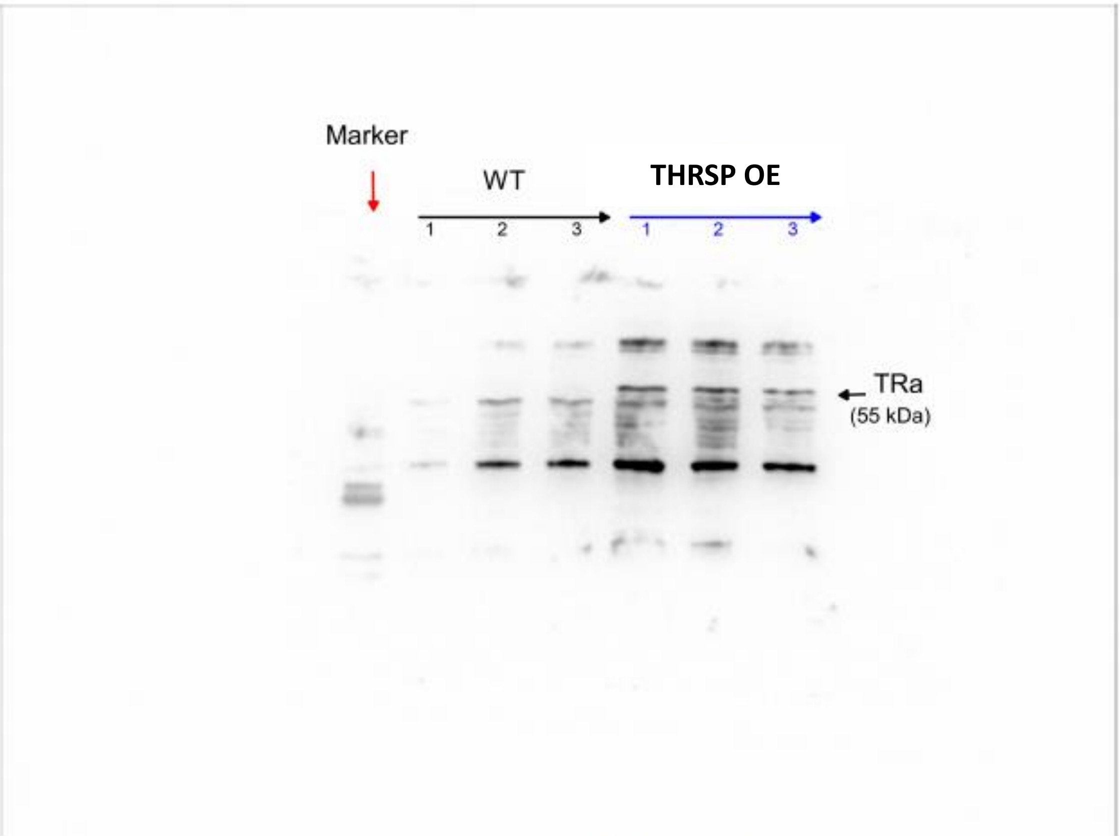

C:\Users\Admin\OneDrive\THRSP OE and KO mice\For PUBLICATION\Western blot\FINAL - western blot\T3\TRa (WT vs THRSP OE - T3 - 1-3).scn

---

Acquisition Information

Imager	ChemiDoc MP
Exposure Time (sec)	6.398 (Auto - Intense Bands)
Flat Field	Applied (Lens)
Serial Number	731BR03542
Software Version	5.2.1
Application	Chemi
Excitation Source	No Illumination
Emission Filter	No Filter
Binning	3x3

TRa (WT vs THRSP OE - T3 - 4-6)

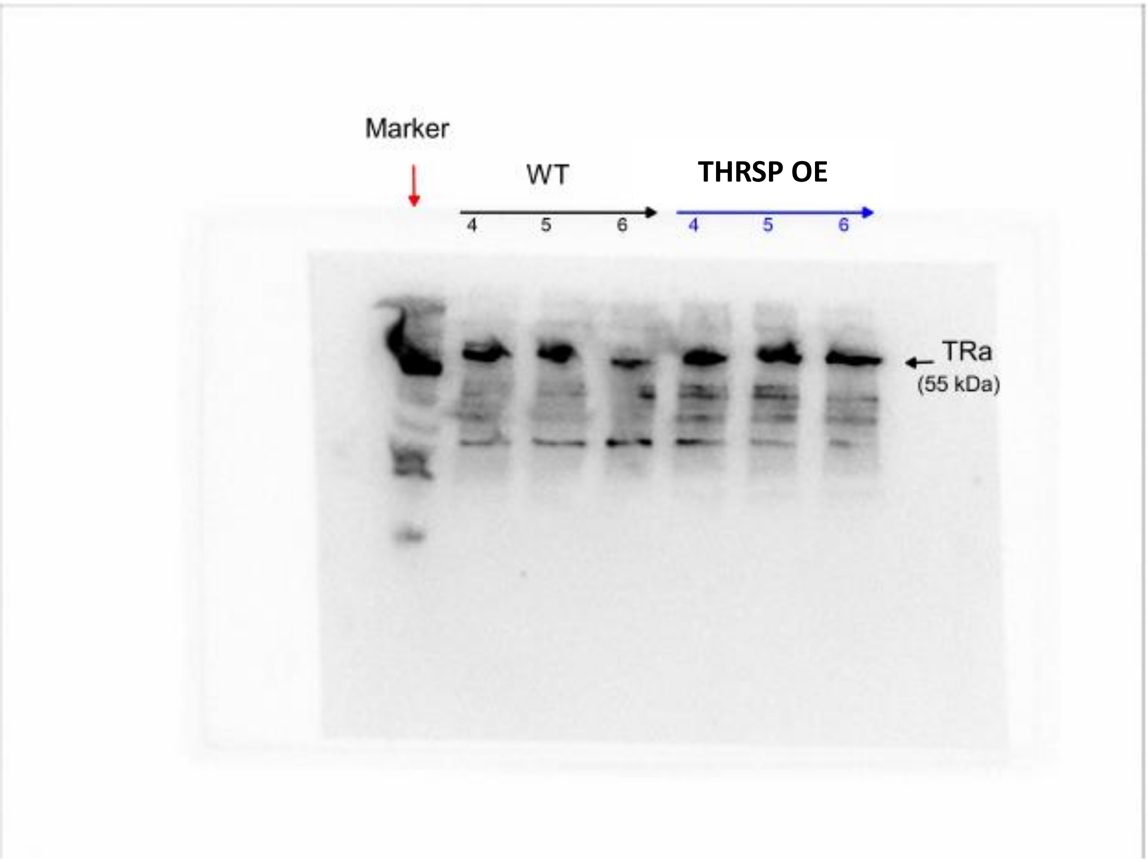

C:\Users\Admin\OneDrive\THRSP OE and KO mice\For PUBLICATION\Western blot\FINAL - western blot\T3\TRa (WT vs THRSP OE - T3 - 4-6).scn

---

Acquisition Information

Imager	ChemiDoc MP
Exposure Time (sec)	3.145 (Auto - Intense Bands)
Flat Field	Applied (Lens)
Serial Number	731BR03542
Software Version	5.2.1
Application	Chemi
Excitation Source	No Illumination
Emission Filter	No Filter
Binning	3x3

TRb (WT vs THRSP OE - T3 - 1-3)

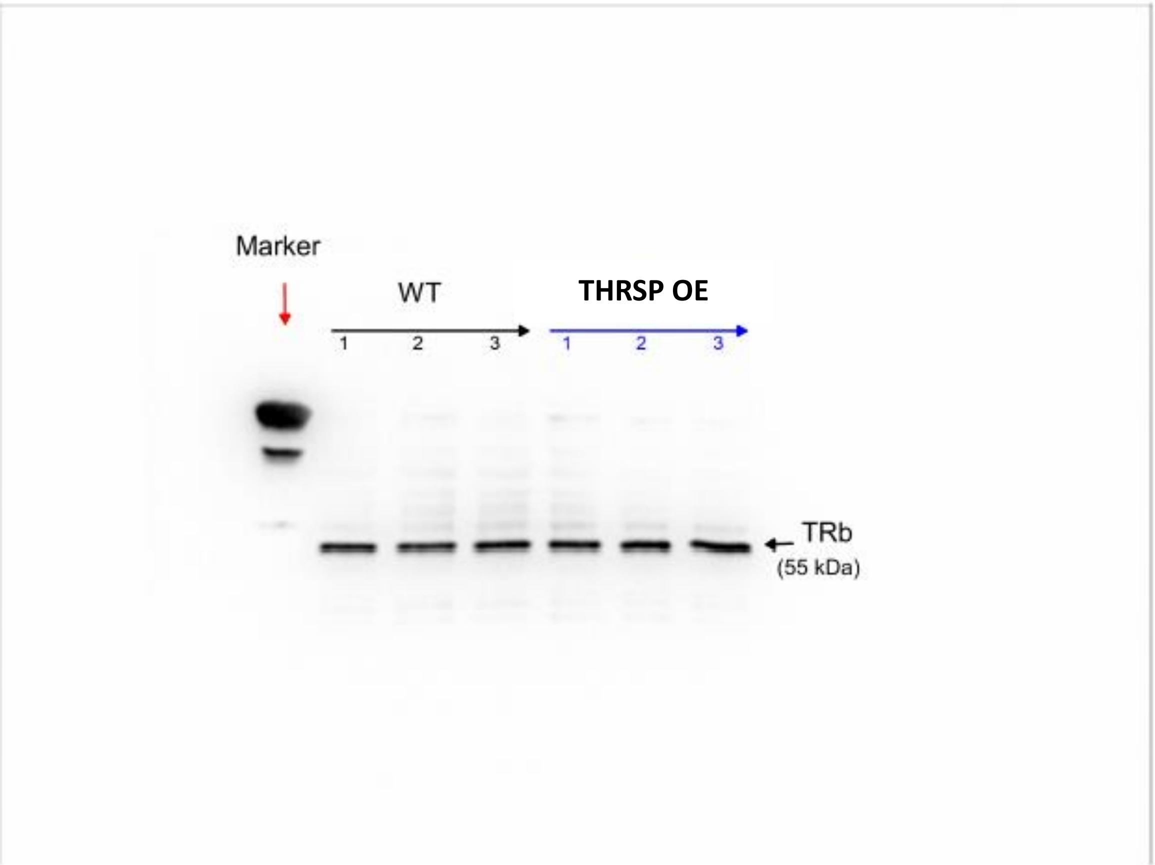

C:\Users\Admin\OneDrive\THRSP OE and KO mice\For PUBLICATION\Western blot\FINAL - western blot\T3\TRb (WT vs THRSP OE - T3 - 1-3).scn

Acquisition Information

Imager	ChemiDoc MP
Exposure Time (sec)	3.330 (Auto - Intense Bands)
Flat Field	Applied (Lens)
Serial Number	731BR03542
Software Version	5.2.1
Application	Chemi
Excitation Source	No Illumination
Emission Filter	No Filter
Binning	3x3

TRb (WT vs THRSP OE - T3 - 4-6)

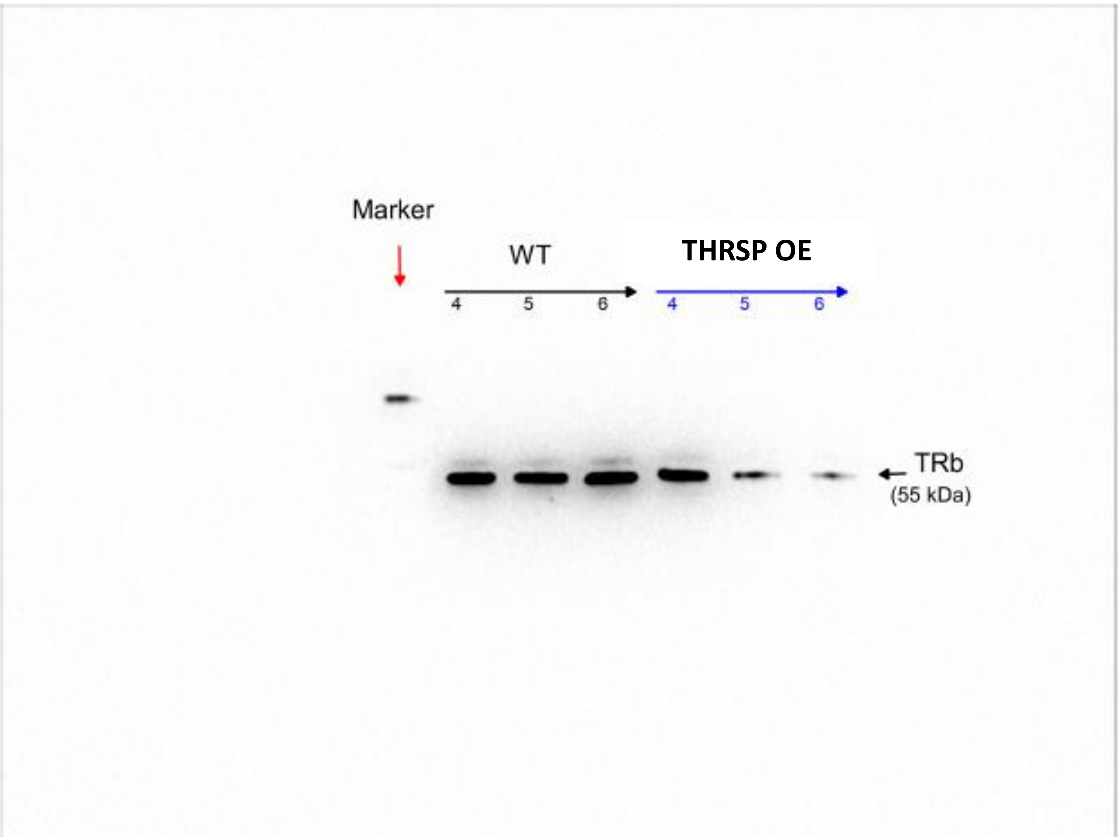

C:\Users\Admin\OneDrive\THRSP OE and KO mice\For PUBLICATION\Western blot\FINAL - western blot\T3\TRb (WT vs THRSP OE - T3 - 4-6).scn

---

Acquisition Information

Imager	ChemiDoc MP
Exposure Time (sec)	3.078 (Auto - Intense Bands)
Flat Field	Applied (Lens)
Serial Number	731BR03542
Software Version	5.2.1
Application	Chemi
Excitation Source	No Illumination
Emission Filter	No Filter
Binning	3x3

B-actin (WT vs THRSP OE - T4 - 1-3)

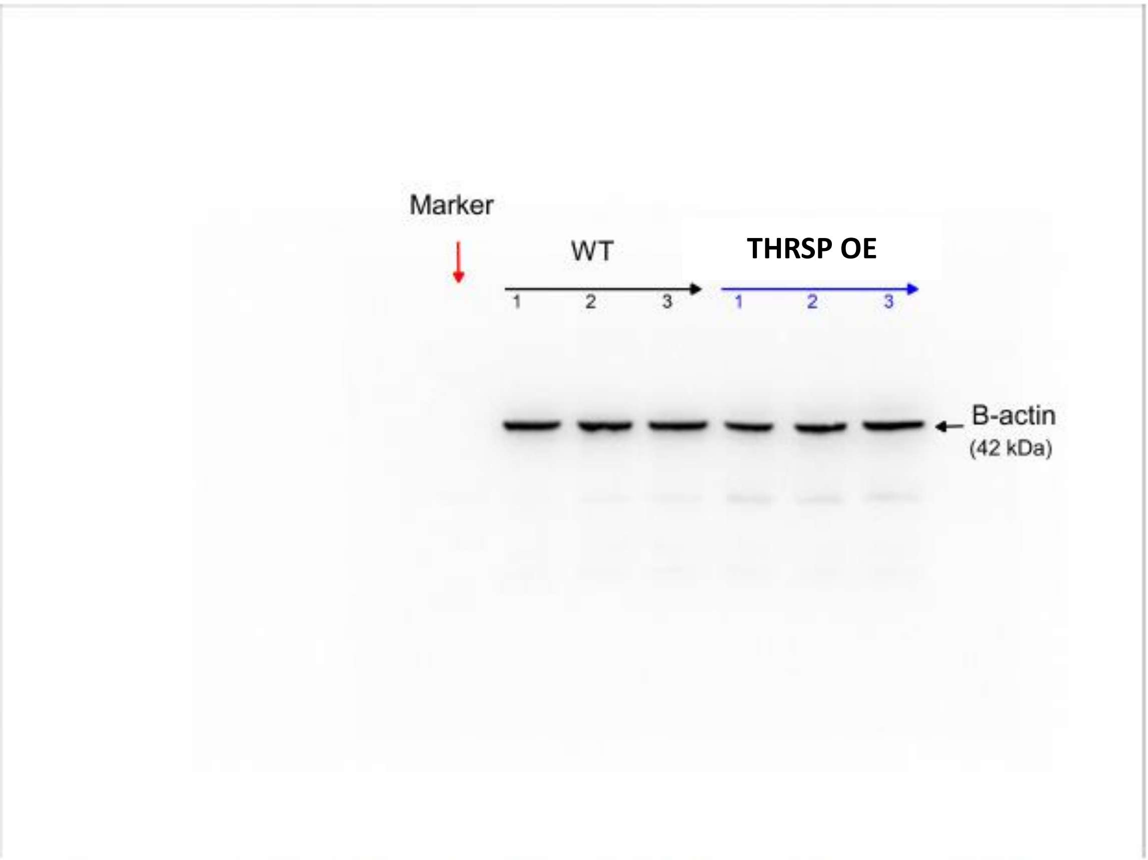

C:\Users\Admin\OneDrive\THRSP OE and KO mice\For PUBLICATION\Western blot\FINAL - western blot\T4\B-actin (WT vs THRSP OE - T4 - 1-3).scn

---

Acquisition Information

Imager	ChemiDoc MP
Exposure Time (sec)	2.494 (Auto - Intense Bands)
Flat Field	Applied (Lens)
Serial Number	731BR03542
Software Version	5.2.1
Application	Chemi
Excitation Source	No Illumination
Emission Filter	No Filter
Binning	3x3

B-actin (WT vs THRSP OE - T4 - 4-6)

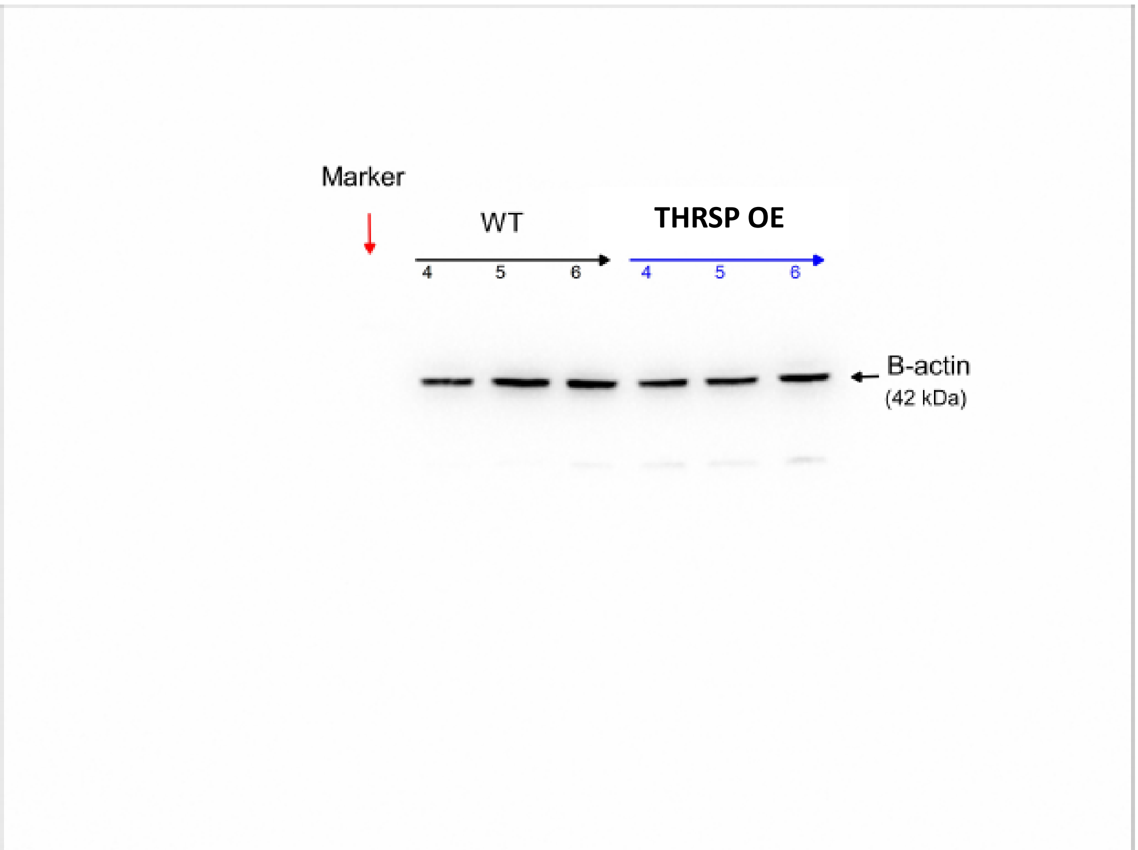

C:\Users\Admin\OneDrive\THRSP OE and KO mice\For PUBLICATION\Western blot\FINAL - western blot\T4\B-actin (WT vs THRSP OE - T4 - 4-6).scn

---

Acquisition Information

Imager	ChemiDoc MP
Exposure Time (sec)	3.652 (Auto - Intense Bands)
Flat Field	Applied (Lens)
Serial Number	731BR03542
Software Version	5.2.1
Application	Chemi
Excitation Source	No Illumination
Emission Filter	No Filter
Binning	3x3

MCT8 (WT vs THRSP OE - T4 - 1-3)

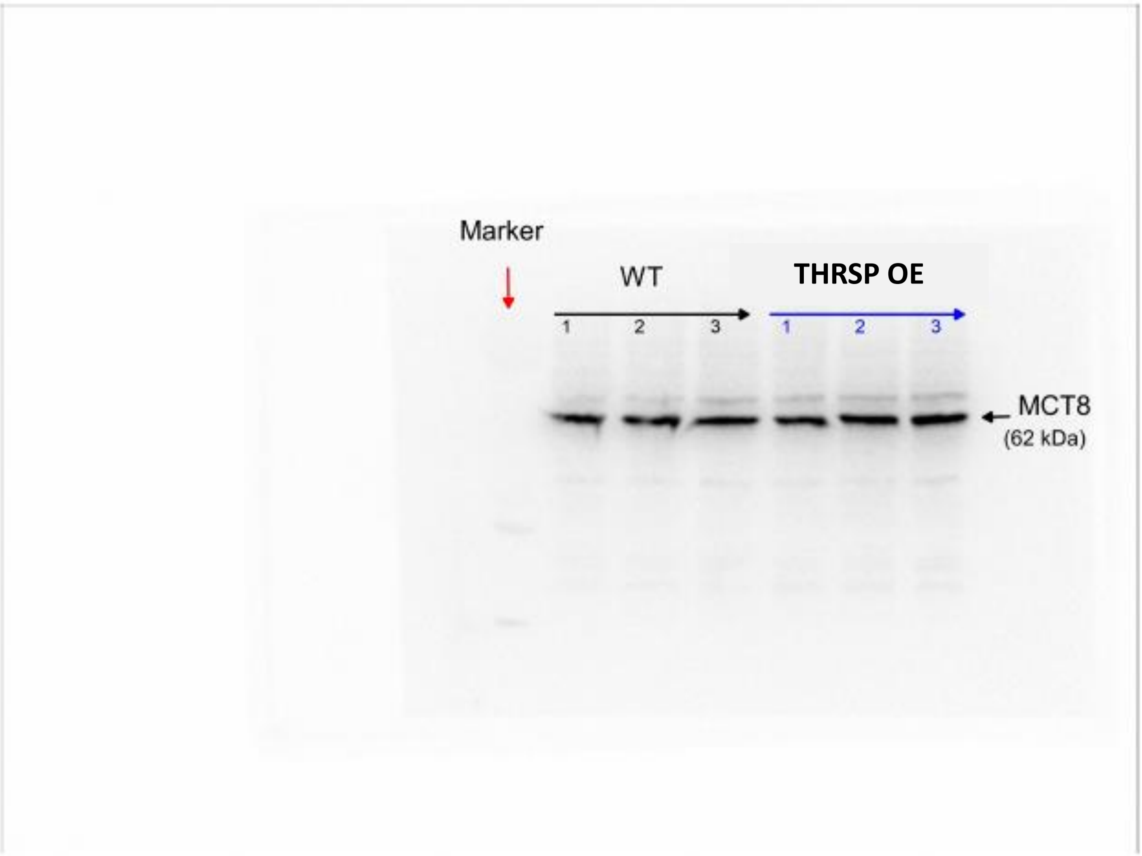

C:\Users\Admin\OneDrive\THRSP OE and KO mice\For PUBLICATION\Western blot\FINAL - western blot\T4\MCT8 (WT vs THRSP OE - T4 - 1-3).scn

---

Acquisition Information

Imager	ChemiDoc MP
Exposure Time (sec)	5.527 (Auto - Intense Bands)
Flat Field	Applied (Lens)
Serial Number	731BR03542
Software Version	5.2.1
Application	Chemi
Excitation Source	No Illumination
Emission Filter	No Filter
Binning	3x3

MCT8 (WT vs THRSP OE - T4 - 4-6)

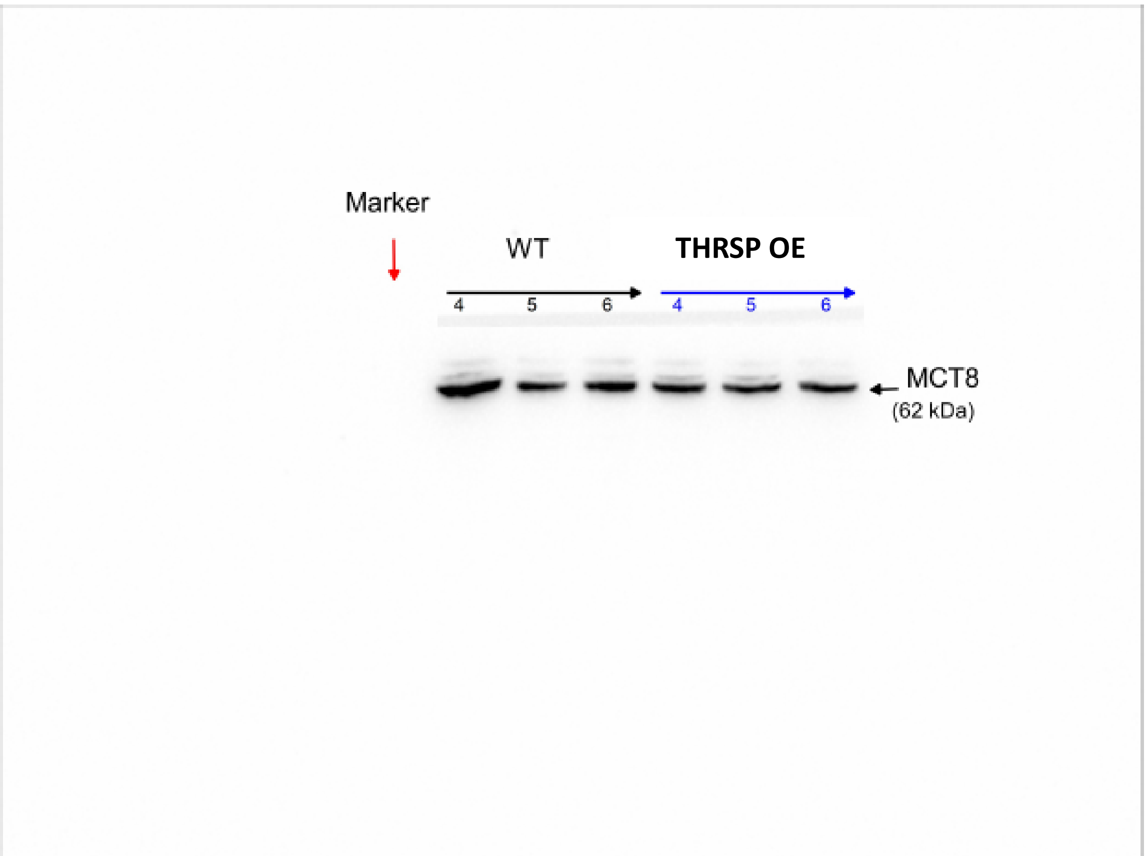

C:\Users\Admin\OneDrive\THRSP OE and KO mice\For PUBLICATION\Western blot\FINAL - western blot\T4\MCT8 (WT vs THRSP OE - T4 - 4-6).scn

---

Acquisition Information

Imager	ChemiDoc MP
Exposure Time (sec)	2.363 (Auto - Intense Bands)
Flat Field	Applied (Lens)
Serial Number	731BR03542
Software Version	5.2.1
Application	Chemi
Excitation Source	No Illumination
Emission Filter	No Filter
Binning	3x3

TRa (WT vs THRSP OE - T4 - 1-3)

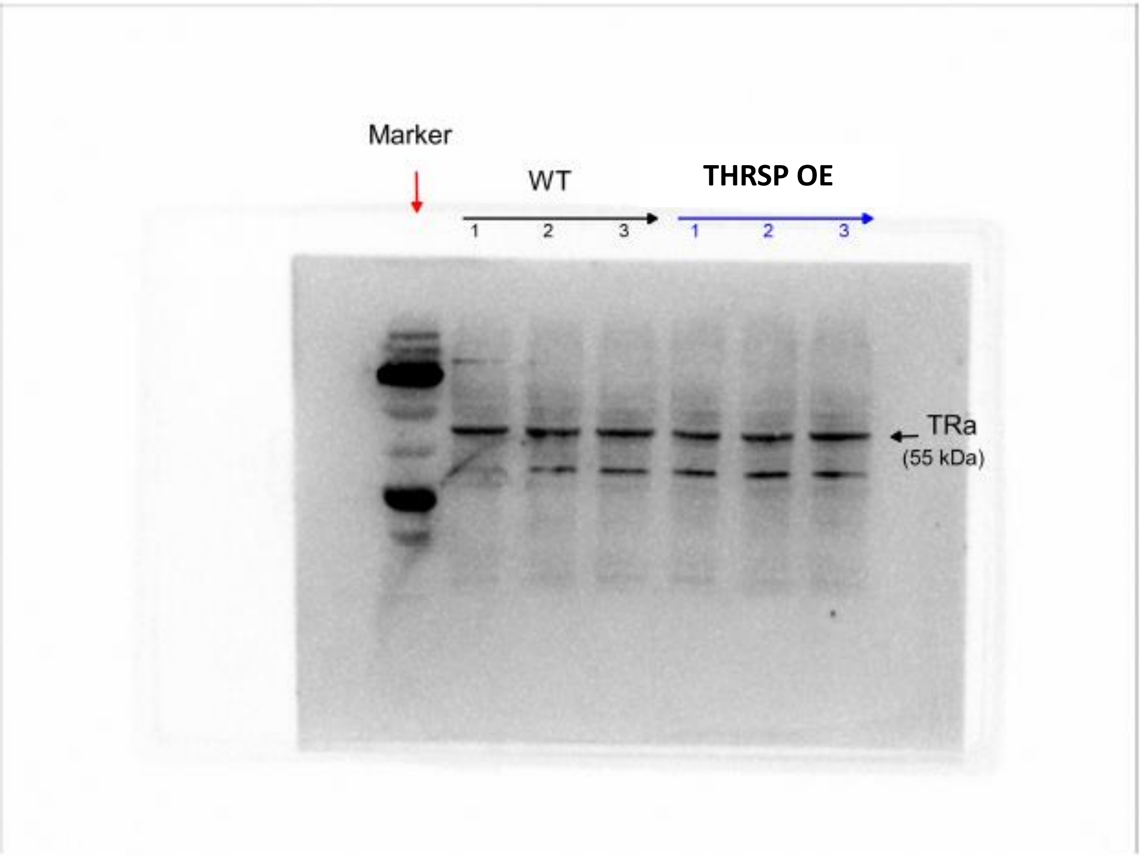

C:\Users\Admin\OneDrive\THRSP OE and KO mice\For PUBLICATION\Western blot\FINAL - western blot\T4\TRa (WT vs THRSP OE - T4 - 1-3).scn

---

Acquisition Information

Imager	ChemiDoc MP
Exposure Time (sec)	6.386 (Auto - Intense Bands)
Flat Field	Applied (Lens)
Serial Number	731BR03542
Software Version	5.2.1
Application	Chemi
Excitation Source	No Illumination
Emission Filter	No Filter
Binning	3x3

TRa (WT vs THRSP OE - T4 - 4-6)

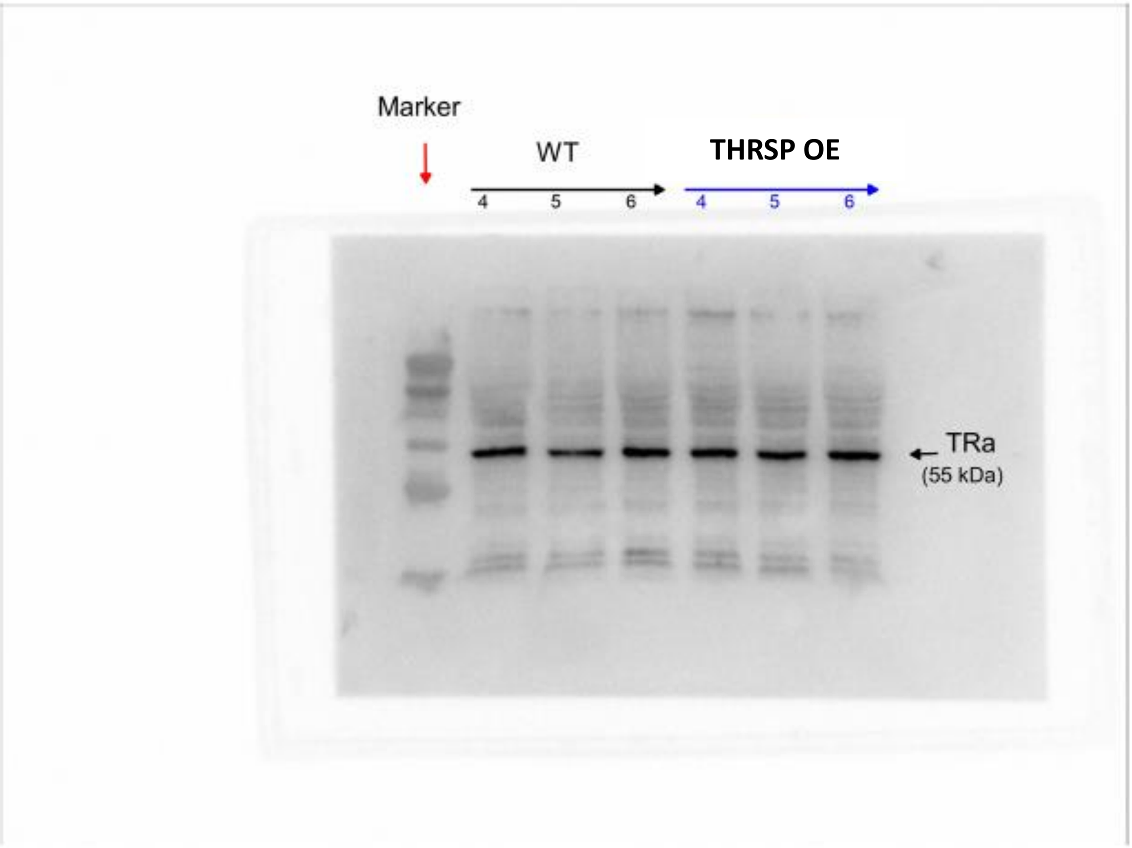

C:\Users\Admin\OneDrive\THRSP OE and KO mice\For PUBLICATION\Western blot\FINAL - western blot\T4\TRa (WT vs THRSP OE - T4 - 4-6).scn

---

Acquisition Information

Imager	ChemiDoc MP
Exposure Time (sec)	2.568 (Auto - Intense Bands)
Flat Field	Applied (Lens)
Serial Number	731BR03542
Software Version	5.2.1
Application	Chemi
Excitation Source	No Illumination
Emission Filter	No Filter
Binning	3x3

TRb (WT vs THRSP OE - T4 - 1-3)

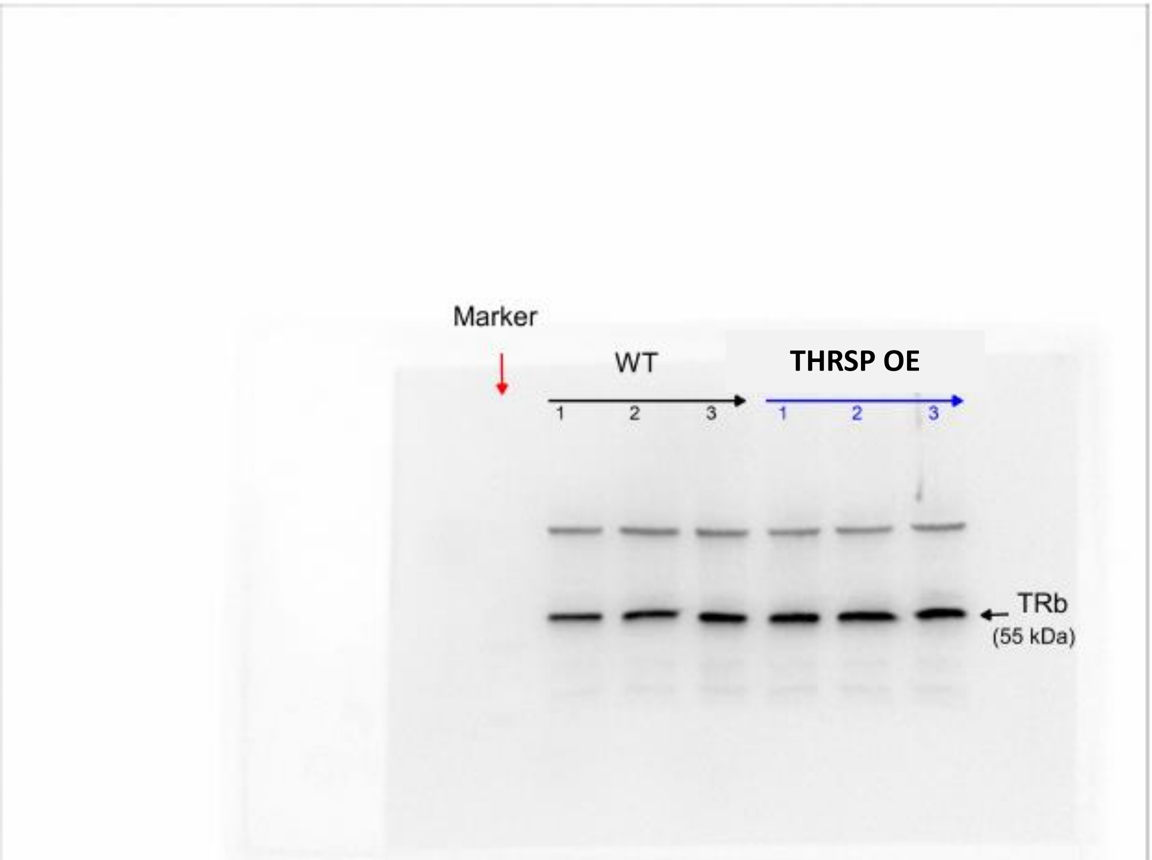

C:\Users\Admin\OneDrive\THRSP OE and KO mice\For PUBLICATION\Western blot\FINAL - western blot\T4\TRb (WT vs THRSP OE - T4 - 1-3).scn

Acquisition Information

Imager	ChemiDoc MP
Exposure Time (sec)	5.526 (Auto - Intense Bands)
Flat Field	Applied (Lens)
Serial Number	731BR03542
Software Version	5.2.1
Application	Chemi
Excitation Source	No Illumination
Emission Filter	No Filter
Binning	3x3

TRb (WT vs THRSP OE - T4 - 4-6)

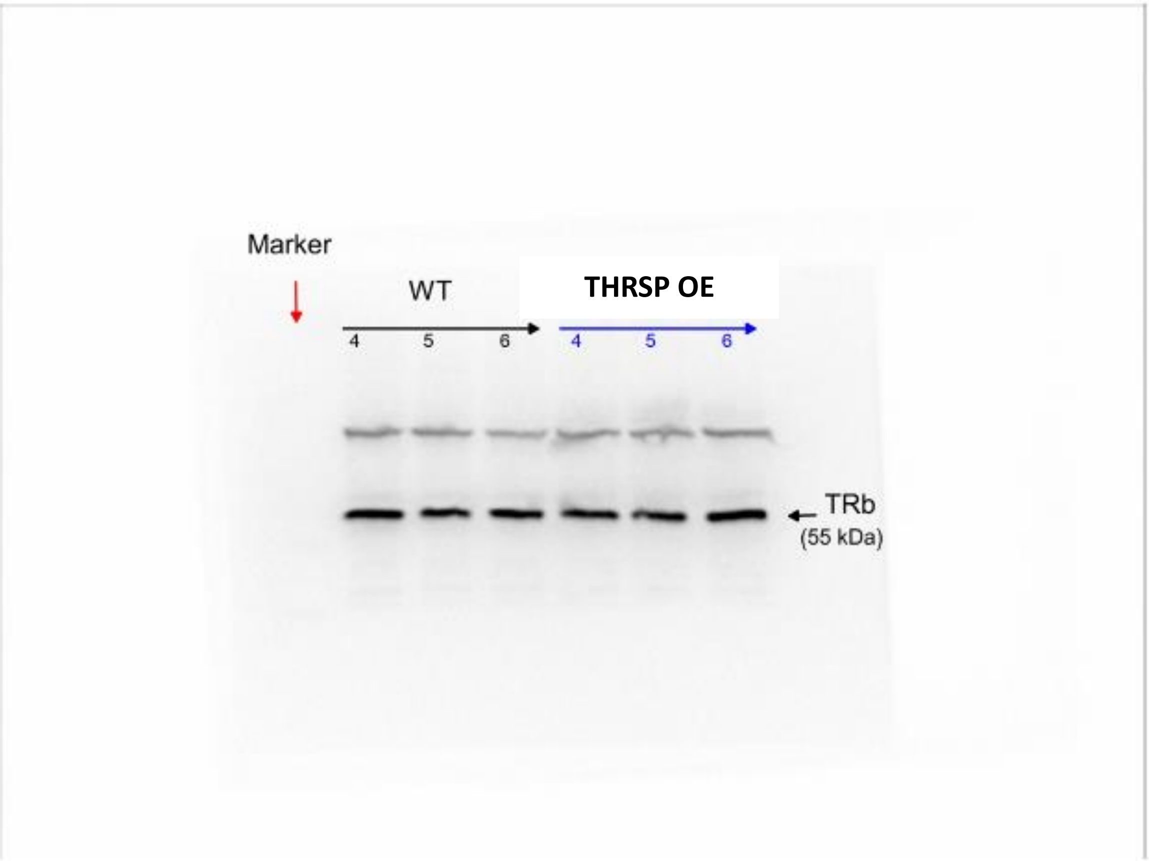

C:\Users\Admin\OneDrive\THRSP OE and KO mice\For PUBLICATION\Western blot\FINAL - western blot\T4\TRb (WT vs THRSP OE - T4 - 4-6).scn

---

Acquisition Information

Imager	ChemiDoc MP
Exposure Time (sec)	4.306 (Auto - Intense Bands)
Flat Field	Applied (Lens)
Serial Number	731BR03542
Software Version	5.2.1
Application	Chemi
Excitation Source	No Illumination
Emission Filter	No Filter
Binning	3x3
