# Supplementary material for: Low striatal T3 is implicated in inattention and memory impairment in an ADHD mouse model overexpressing thyroid hormone-responsive protein
Source: Commun Biol. 2021 Sep 20;4:1101. doi: 10.1038/s42003-021-02633-w (PMC8452653; doi:10.1038/s42003-021-02633-w)
Supplement: Supplementary file 3 — Description of Additional Supplementary Files [file 42003_2021_2633_MOESM3_ESM.pdf]

Description of Additional Supplementary Files

File name: Supplementary Data 1.

Description: Contains the statistical analysis for each figure presented and the raw datasets used for analysis.
