# Supplementary material for: Low striatal T3 is implicated in inattention and memory impairment in an ADHD mouse model overexpressing thyroid hormone-responsive protein
Source: Commun Biol. 2021 Sep 20;4:1101. doi: 10.1038/s42003-021-02633-w (PMC8452653; doi:10.1038/s42003-021-02633-w)
Supplement: Supplementary file 5 — Reporting summary [file 42003_2021_2633_MOESM5_ESM.pdf]

nature research

Corresponding author(s): Dr. Jae Hoon Cheong; Dr Hee Jin Kim

Last updated by author(s): Aug 10, 2021

Reporting Summary

Nature Research wishes to improve the reproducibility of the work that we publish. This form provides structure for consistency and transparency in reporting. For further information on Nature Research policies, see our Editorial Policies and the Editorial Policy Checklist.

Statistics

For all statistical analyses, confirm that the following items are present in the figure legend, table legend, main text, or Methods section.

n/a	Confirmed	
☐	☒	The exact sample size (n) for each experimental group/condition, given as a discrete number and unit of measurement
☐	☒	A statement on whether measurements were taken from distinct samples or whether the same sample was measured repeatedly
☐	☒	The statistical test(s) used AND whether they are one- or two-sided<br>*Only common tests should be described solely by name; describe more complex techniques in the Methods section.*
☐	☒	A description of all covariates tested
☐	☒	A description of any assumptions or corrections, such as tests of normality and adjustment for multiple comparisons
☐	☒	A full description of the statistical parameters including central tendency (e.g. means) or other basic estimates (e.g. regression coefficient) AND variation (e.g. standard deviation) or associated estimates of uncertainty (e.g. confidence intervals)
☐	☒	For null hypothesis testing, the test statistic (e.g. F, t, r) with confidence intervals, effect sizes, degrees of freedom and P value noted<br>*Give P values as exact values whenever suitable.*
☐	☒	For Bayesian analysis, information on the choice of priors and Markov chain Monte Carlo settings
☐	☒	For hierarchical and complex designs, identification of the appropriate level for tests and full reporting of outcomes
☐	☒	Estimates of effect sizes (e.g. Cohen's d, Pearson's r), indicating how they were calculated

Our web collection on statistics for biologists contains articles on many of the points above.

Software and code

Policy information about availability of computer code

Data collection	Ethovison XT
Data analysis	Graphpad Prism v9

For manuscripts utilizing custom algorithms or software that are central to the research but not yet described in published literature, software must be made available to editors and reviewers. We strongly encourage code deposition in a community repository (e.g. GitHub). See the Nature Research guidelines for submitting code & software for further information.

Data

Policy information about availability of data

All manuscripts must include a data availability statement. This statement should provide the following information, where applicable:

- Accession codes, unique identifiers, or web links for publicly available datasets
- A list of figures that have associated raw data
- A description of any restrictions on data availability

The authors declare that the data supporting the findings of this study are available within the paper and its supplementary information files.

Field-specific reporting

Please select the one below that is the best fit for your research. If you are not sure, read the appropriate sections before making your selection.

☒ Life sciences ☐ Behavioural & social sciences ☐ Ecological, evolutionary & environmental sciences

For a reference copy of the document with all sections, see nature.com/documents/nr-reporting-summary-flat.pdf

Life sciences study design

All studies must disclose on these points even when the disclosure is negative.

Sample size	For graphical purposes, data are presented as the mean ± standard error (SEM), and all statistical analyses were conducted on raw data tested for normal (Gaussian) distribution using D'Agostino-Pearson omnibus. In all available figures, the animal numbers and recorded data points were clearly indicated. Results were analyzed using either a t-test, one-way, or two-way analysis of variance (ANOVA) with or without repeated measures (RM), followed by Dunnett's multiple comparisons test. A level of probability of $P \leq 0.05$ was defined as the threshold for statistical significance.
Data exclusions	No data were excluded from the analysis.
Replication	Preliminary experiments were conducted and all attempts at replication (3-4x) were successful.
Randomization	Samples allocation were randomly selected.
Blinding	All investigators were blinded to group allocation during data collection and/or analysis.

Reporting for specific materials, systems and methods

We require information from authors about some types of materials, experimental systems and methods used in many studies. Here, indicate whether each material, system or method listed is relevant to your study. If you are not sure if a list item applies to your research, read the appropriate section before selecting a response.

Materials & experimental systems

n/a	Involved in the study	
☐	☒	Antibodies
☒	☐	Eukaryotic cell lines
☒	☐	Palaeontology and archaeology
☐	☒	Animals and other organisms
☒	☐	Human research participants
☒	☐	Clinical data
☒	☐	Dual use research of concern

Methods

n/a	Involved in the study	
☒	☐	ChIP-seq
☒	☐	Flow cytometry
☒	☐	MRI-based neuroimaging

Antibodies

Antibodies used	1. anti-THRSP (1/1000)<br>2. anti-MCT8 (1/1000)<br>3. anti-TRα (1/250)<br>4. anti-TRβ (1/1000)<br>5. anti-β-actin (1/5000)
Validation	1. rabbit polyclonal anti-THRSP (Thermo Fisher Scientific Cat# PA5-77177, RRID:AB_2720904)<br>2. rabbit polyclonal anti-MCT8 (Fitzgerald Cat#70R-50398)<br>3. rabbit polyclonal anti-TRα (Bioss Cat# bs-6221R, RRID:AB_11072581)<br>4. mouse monoclonal anti-TRβ (Thermo Fisher Scientific Cat# MA1-216, RRID:AB_2287303)<br>5. mouse monoclonal anti-β-actin (Sigma-Aldrich Cat# A5441, RRID:AB_476744)

Animals and other organisms

Policy information about studies involving animals; ARRIVE guidelines recommended for reporting animal research

Laboratory animals	1. C57BL/6 (used as a background strain for all transgenic animals).<br>2. C57BL/6 male (used as control).<br>2. THRSP-overexpressing (THRSP OE) male mice.

	3. THRSP Hetero (heterozygous) male mice.<br>4. THRSP KO (homozygous) male mice.
Wild animals	The study did not involve wild animals.
Field-collected samples	The study did not involve samples collected in the field.
Ethics oversight	1. Principles of Laboratory Animal Care (NIH Publication No. 85-23, revised 1985).<br>2. Animal Ethics Review Board of Sahmyook University, South Korea (SYUIACUC2020-010).

Note that full information on the approval of the study protocol must also be provided in the manuscript.
