# Supplementary material for: Low striatal T3 is implicated in inattention and memory impairment in an ADHD mouse model overexpressing thyroid hormone-responsive protein
Source: Commun Biol. 2021 Sep 20;4:1101. doi: 10.1038/s42003-021-02633-w (PMC8452653; doi:10.1038/s42003-021-02633-w)
Supplement: Supplementary file 1 — Peer Review File [file 42003_2021_2633_MOESM1_ESM.pdf]

nature portfolio

Peer Review File

Low striatal T3 is implicated in inattention and memory impairment in an ADHD model overexpressing thyroid hormone-responsive protein.

Open Access This file is licensed under a Creative Commons Attribution 4.0 International License, which permits use, sharing, adaptation, distribution and reproduction in any medium or format, as long as you give appropriate credit to the original author(s) and the source, provide a link to the Creative Commons license, and indicate if changes were made. In the cases where the authors are anonymous, such as is the case for the reports of anonymous peer reviewers, author attribution should be to 'Anonymous Referee' followed by a clear attribution to the source work. The images or other third party material in this file are included in the article's Creative Commons license, unless indicated otherwise in a credit line to the material. If material is not included in the article's Creative Commons license and your intended use is not permitted by statutory regulation or exceeds the permitted use, you will need to obtain permission directly from the copyright holder. To view a copy of this license, visit http://creativecommons.org/licenses/by/4.0/.

Reviewers' comments:

Reviewer #1 (Remarks to the Author):

According to the manuscript, the present study showed that the involvement of upregulated THRSP in ADHD and indicates that THRSP OE mice can serve as a potential animal model for ADHD-PI. But there are some points should be revised which have not be described fully. These concerns have been listed below which may contribute to improve the manuscript.

Specific comments to authors:
1.How did you choose the dose for each group? The injection way was not detailed enough. Please specify it.
2.What is the basis for sequential selection of behavioral experiments?
3.The number of replicates is not well described.
4. The results of beta actin were inconsistent of figure 1 and beta actin was inconsistent in VEH of figure 10.
5. Please reorganize the Discussion section to make it more focused and logical. Is there a closer relationship between the results? If there is evidence, please add it.

Reviewer #2 (Remarks to the Author):

This is a well-written manuscript describing interesting novel findings about the role of thyroid hormone in cognitive function in mice using a THRSP- overexpression mouse model. The authors suggest that this mouse model may be a pre-clinical model of predominantly inattentive type of ADHD.

1. A major concern is that the novel object recognition test is used here as a test of attention, but it is not. A modification of the novel object recognition test called the object-based attention test has been used as a measure of object-based attention in rodent models (doi: 10.1016/j.bbr.2011.01.039, PMID: 21277334; doi: 10.1016/j.bbr.2012.10.058, PMID: 23142610; doi: 10.1371/journal.pone.0198064. PubMed PMID: 29795664; PMCID: PMC5967717; doi: 10.1016/j.ijdevneu.2017.01.014. PubMed PMID: 28179105). The validity of the object-based attention test has been established in prenatal nicotine exposure mouse models of ADHD because the mouse model also shows attention deficit in the 5-choice serial reaction time test, considered by many as the standard test for attention in rodents. The novel object recognition test is test of recognition memory. This is a major concern, as it impacts the conclusions drawn by the authors.

2. The Y-maze is a test of spatial working memory and the Barnes maze (as used here) is a test of spatial learning and reference memory. The authors did not make these distinctions.

3. The authors did not clarify if overexpression of THRSP has construct validity for ADHD. In other words, is there evidence that THSRP overexpression is associated with ADHD?

3. Abstract: State that only male mice were used.

4. It is customary to place visual cues on the walls of the room where the Barnes maze test is performed. These visual cues offer spatial reference. Were such cues used?

5. Lines 236-237: The meaning of the sentence "This indicates a transgenerational transmission of inattentive behavior in a mouse model of ADHD" is not clear. What is meant by transgenerational transmission here?

6. Line 258. What is "random probability"? Is it a reference to the strategy adopted by the mice as they navigated the Barnes maze - serial versus random? Please clarify.

7. The reference to "ADHD features" in a mouse model is inaccurate. Mice do not have ADHD, although some of the behaviors exhibited by the mice may be consistent with the symptoms of ADHD.

8. Administration of the drugs: The drug administration schedule should be stated in the Methods section. The effects of the drugs on performance in the Y-maze and NOR test appears to be an acute effect of a single administration (one assumes) whereas in the Barnes maze, the mice received the drug for 7 days. The effects on biochemical measurements were examined following a 7-day drug treatment period. How can the effects of the drugs on Y-maze and NOR be explained by the biochemical data? These points need to be clarified.

9. The statistical analyses are not described well. For example, in the legend to Figure 6, F values are stated for the two-way ANOVA without stating the factor to which the F values may apply. In Figure 6c the drug LT3 appears to have improved performance in the wild type mice as well as in the OE mice. But, statistical comparison appears to have been performed only for the OE mice. These types of details are critical and should be described for all statistical analyses (not just for Figure 6).

Response to Referees

We would like to thank the referees for their time in reviewing our manuscript and for their insightful critiques and suggestions. We have carefully revised our manuscript in accordance with the comments provided. All necessary information that were added in the revision are highlighted in yellow. We hope that we have satisfactorily answered all the queries raised.

Reviewer #1:

According to the manuscript, the present study showed that the involvement of upregulated THRSP in ADHD and indicates that THRSP OE mice can serve as a potential animal model for ADHD-PI. But there are some points should be revised which have not be described fully. These concerns have been listed below which may contribute to improve the manuscript.

Comment 1: How did you choose the dose for each group? The injection way was not detailed enough. Please specify it.

Response: The dose used in the study was based on previously published data (PMID: 20236931) and followed the drug safety assessment strategies (PMID: 31220983) by establishing a safe starting dose level, maximal tolerable dose, and exposure to or frequency of drug treatment. These are provided in the "Drugs" and subsections of the "Behavioral tests" under Materials and Methods.

Comment 2: What is the basis for sequential selection of behavioral experiments?

Response: The sequence of behavioral experiments were patterned from our previous published study (PMID: 30138648) where we exposed the THRSP OE mice and WT mice to a battery of behavioral tests that measure the core symptoms of ADHD (i.e., inattention, hyperactivity, impulsivity) and other possible comorbid behavioral disorders (i.e., anxiety, motor balance impairment), which were conducted from a "least-stressful" to a "more-stressful" fashion.

Comment 3: The number of replicates is not well described.

Response: All experiments were replicated at least three times before reaching a conclusion (either a positive or a negative result). This statement is now included in the revision.

Comment 4: The results of beta actin were inconsistent of figure 1 and beta actin was inconsistent in VEH of figure 10.

Response: We have provided a correct compilation of untruncated western blots which can be found in the supplementary information.

Comment 5: Please reorganize the Discussion section to make it more focused and logical. Is there a closer relationship between the results? If there is evidence, please add it.

Response: A revision has been provided in the 'Discussion' and 'Conclusion' section.

Reviewer #2:

This is a well-written manuscript describing interesting novel findings about the role of thyroid hormone in cognitive function in mice using a THRSP- overexpression mouse model. The authors suggest that this mouse model may be a pre-clinical model of predominantly inattentive type of ADHD.

Comment 1: A major concern is that the novel object recognition test is used here as a test of attention, but it is not. A modification of the novel object recognition test called the object-based attention test has been used as a measure of object-based attention in rodent models (doi: 10.1016/j.bbr.2011.01.039, PMID: 21277334; doi: 10.1016/j.bbr.2012.10.058, PMID: 23142610; doi: 10.1371/journal.pone.0198064. PubMed PMID: 29795664; PMCID: PMC5967717; doi: 10.1016/j.ijdevneu.2017.01.014. PubMed PMID: 28179105). The validity of the object-based attention test has been established in prenatal nicotine exposure mouse models of ADHD because the mouse model also shows attention deficit in the 5-choice serial reaction time test, considered by many as the standard test for attention in rodents. The novel object recognition test is test of recognition memory. This is a major concern, as it impacts the conclusions drawn by the authors.

Response: Inarguably, the object recognition test was originally developed as a tool to measure memory (PMID: 3228475), however, more recently, studies have been utilizing the novel-object recognition test to measure attention as well, particularly those used in modeling ADHD-like behaviors in rodents (e.g., PMID: 25151620, PMID: 30138648). It is because during “novelty” or when something new to the environment is present, attention, as well as exploration is involved. This allows subjects to examine the objects present either closely or distally, depending on the risks. Moreover, if something familiar is present, it also requires attention and reevaluation from the subjects (PMID: 22160349). In our results, we found that THRSP OE mice had lower investigation time which is observed during the familiarization phase of the novel-object recognition test. A decreased time spent investigating the objects during the familiarization phase has been suggested as an initial display of inattentive behavior or inattention (PMID: 25151620). Thus, the decreased discrimination index of THRSP OE mice might also be a result of their inattention to the objects in the familiarization phase. This information has been included in the discussions section.

Comment 2: The Y-maze is a test of spatial working memory and the Barnes maze (as used here) is a test of spatial learning and reference memory. The authors did not make these distinctions.

Response: Like NORT, Y-maze test has also been developed to measure memory, however, its use in modeling inattention has already been performed in several ADHD-directed studies (e.g., PMID: 25151620, PMID: 26048425, PMID: 30125623, PMID: 27996970). In fact, the Spontaneous Alternation behaviors is regarded as an index of attention. Thus, this behavioral test was conducted in the previous (PMID: 30138648) and the present study.

Comment 3: The authors did not clarify if overexpression of THRSP has construct validity for ADHD. In other words, is there evidence that THSRP overexpression is associated with ADHD?

Response: Previously, we have identified that the inattentive THRSP OE mice have altered dopaminergic-related genes (PMID: 30138648) (i.e., tyrosine hydroxylase, dopamine transporter, d1 receptors, d2 receptors) which were normalized by methylphenidate treatment which suggests not just face and predictive validity but construct validity as well. Information is included in the 'conclusion' section.

Comment 4: Abstract: State that only male mice were used.

Response: These were added in the revised form.

Comment 5: It is customary to place visual cues on the walls of the room where the Barnes maze test is performed. These visual cues offer spatial reference. Were such cues used?

Response: Yes, visual cues were placed around the maze which acts as spatial cues for mice to help them navigate the platform.

Comment 6: Lines 236-237: The meaning of the sentence "This indicates a transgenerational transmission of inattentive behavior in a mouse model of ADHD" is not clear. What is meant by transgenerational transmission here?

Response: This indicates that the "behavioral phenotype" of inattention previously observed in THRSP OE mice is still manifested in newer generations. A revision has been made in the discussion section.

Comment 7: Line 258. What is "random probability"? Is it a reference to the strategy adopted by the mice as they navigated the Barnes maze - serial versus random? Please clarify.

Response: We apologize for this. Yes, this refers to the navigational strategy used by THRSP OE mice which were in random. A revision has been made in the discussion section.

Comment 8: The reference to "ADHD features" in a mouse model is inaccurate. Mice do not have ADHD, although some of the behaviors exhibited by the mice may be consistent with the symptoms of ADHD.

Response: This term was replaced with "ADHD-like behaviors".

Comment 9: Administration of the drugs: The drug administration schedule should be stated in the Methods section. The effects of the drugs on performance in the Y-maze and NOR test

appears to be an acute effect of a single administration (one assumes) whereas in the Barnes maze, the mice received the drug for 7 days. The effects on biochemical measurements were examined following a 7-day drug treatment period. How can the effects of the drugs on Y-maze and NOR be explained by the biochemical data? These points need to be clarified.

Response: We have added the drug administration schedule in the 'Materials and Methods' section whereas, the following points regarding the acute vs long-term effects manifested in the behavioral and biochemical levels were added in the 'Discussion' section.

Comment 10: The statistical analyses are not described well. For example, in the legend to Figure 6, F values are stated for the two-way ANOVA without stating the factor to which the F values may apply. In Figure 6c the drug LT3 appears to have improved performance in the wild type mice as well as in the OE mice. But, statistical comparison appears to have been performed only for the OE mice. These types of details are critical and should be described for all statistical analyses (not just for Figure 6).

Response: We have provided a detailed statistical analysis (including the genotype and treatment effects as well as the interaction of both) for all experiments which are now included as supplementary data.

Reviewers' comments:

Reviewer #1 (Remarks to the Author):

Review of: "Low striatal T3 is implicated in inattention and memory impairment in an ADHD model overexpressing thyroid hormone-responsive protein".

According to the revised manuscript, the concerns have demonstrated clearly and discussed fully. The manuscript has improved. Now we agree to consider accepting it.

Reviewer #2 (Remarks to the Author):

A major comment by this reviewer (comment #1) is not addressed by the revision. The novel object recognition (NOR) test is a test of recognition memory and not a test of attention. The authors have argued to the contrary, but the evidence provided by the authors to defend their claim is tenuous at best. Of course attention is a contributing factor to the phenotype measured by the NOR test, as it is to many phenotypes such as spontaneous locomotor activity, exploratory activity, visual function, auditory functon(to name a few). But tests that are designed to measure those behaviors are not designed to test attention. Therefore, although attention may contribute to recognition memory, the NOR test is not a test of attention. This manuscript is written with attention as a major focus. Therefore, a test that measures attention directly should be used. There are two tests of attention for rodents: 5-choice serial reaction time test and object based attention test. Both tests have their own caveats, but they are considered acceptable by the field.

Overall Response to Referees

We would like to thank the referees for their time in reviewing our manuscript and for their insightful critiques and suggestions.

Reviewer #2 (Remarks to the Author):

A major comment by this reviewer (comment #1) is not addressed by the revision. The novel object recognition (NOR) test is a test of recognition memory and not a test of attention. The authors have argued to the contrary, but the evidence provided by the authors to defend their claim is tenuous at best. Of course attention is a contributing factor to the phenotype measured by the NOR test, as it is to many phenotypes such as spontaneous locomotor activity, exploratory activity, visual function, auditory function (to name a few). But tests that are designed to measure those behaviors are not designed to test attention. Therefore, although attention may contribute to recognition memory, the NOR test is not a test of attention. This manuscript is written with attention as a major focus. Therefore, a test that measures attention directly should be used. There are two tests of attention for rodents: 5-choice serial reaction time test and object-based attention test. Both tests have their own caveats, but they are considered acceptable by the field.

Response: As suggested, we have conducted the object-based attention test (OBAT) to further confirm the inattention in THRSP OE mice. The findings observed in OBAT show that the transgenic mice overexpressing THRSP have reduced recognition index corroborated by a reduced preference (index) for novel object, confirming that THRSP OE mice are indeed inattentive. These can be found in figures 2 (h, i) and 6 (e, f), and in supplementary figure1 (f, g) in the revised manuscript. Additional information was also incorporated on the methods and results section which are highlighted in yellow. We do hope that the revisions in the manuscript would satisfactorily address your concerns.

REVIEWERS' COMMENTS:

Reviewer #2 (Remarks to the Author):

The revision addresses this reviewer's concerns satisfactorily.
